# Substrate-Controlled Diversity-Oriented Synthesis of Novel Polycyclic Frameworks via [4 + 2] and [3 + 2] Annulations of Ninhydrin-Derived MBH Adducts with 3,4-Dihydroisoquinolines

**DOI:** 10.3390/molecules28196761

**Published:** 2023-09-22

**Authors:** Kaikai Wang, Wenwen Zhou, Jun Jia, Junwei Ye, Mengxin Yuan, Jie Yang, Yonghua Qi, Rongxiang Chen

**Affiliations:** 1 School of Pharmacy, Xinxiang University, Xinxiang 453000, China; wangkaikai@xxu.edu.cn (K.W.); h200011250107@163.com (W.Z.); y2559356242@126.com (J.Y.); 13333806426@163.com (M.Y.); qyh@xxu.edu.cn (Y.Q.); 2 Jilin Province Product Quality Supervision and Inspection Institute, Changchun 130012, China; beyond-868@163.com; 3 School of Chemistry & Materials Engineering, Xinxiang University, Xinxiang 453000, China

**Keywords:** substrate-controlled, ninhydrin-derived MBH adducts, 3,4-dihydroisoquinolines, cycloaddition, polycyclic frameworks, Diels-Alder reaction, [3 + 2] cycloaddition

## Abstract

Substrate-controlled diversity-oriented synthesis of polycyclic frameworks via [4 + 2] and [3 + 2] annulations between ninhydrin-derived Morita–Baylis–Hillman (MBH) adducts and 3,4-dihydroisoquinolines under similar reaction conditions have been developed. The reaction provides diversity-oriented synthesis of a series of novel and structurally complex spiro multi heterocyclic skeletons in good yields (up to 87% and 90%, respectively) with excellent diastereoselectivities (up to >25:1 *dr*). In particular, the switchable [4 + 2] and [3 + 2] annulation reactions are controlled by tuning the hydroxyl protecting group on the ninhydrin-derived MBH adduct to deliver structural diverse spiro[indene-2,2′-[1,3]oxazino[2,3-*a*]isoquinoline] and spiro[indene-2,1′-pyrrolo[2,1-*a*]isoquinoline], respectively. Furthermore, the relative configuration and chemical structure of two kinds of cycloadducts were confirmed through X-ray diffraction analysis.

## 1. Introduction

Polycyclic rings are the important frameworks as a core structure widespread throughout most classes of natural and non-natural products with a wide spectrum of pharmacological and biological activities, which makes them arouse ever-increasing attention in organic and medicinal chemistry [1,2,3,4,5,6,7]. The polycyclic compounds containing some privileged structures, such as tetrahydroisoquinoline motifs [8,9,10], tetrahydropyrrole moieties [11,12,13] or indane-1,3-dione units [14,15] and so on, especially play a key role in drug discovery and development. Owing to their usefulness and potential pharmacological activity, a wide array of synthetic methods has been developed to directly construct functional and complex polycyclic scaffolds bearing privileged structures including Diels-Alder reaction [16,17,18,19,20,21], cycloaddition reaction [22,23], cascade reaction [24,25,26,27], etc., from all kinds of reaction partners. Among them, the Morita–Baylis–Hillman (MBH) adducts and their derivatives have been extensively explored due to their multifunctional features, and applied as versatile synthons in diverse annulation reactions, mostly for the construction of polycyclic frameworks via cycloaddition with a large variety dipolarophiles [28,29,30,31,32,33]. In particular, isatins or aldehydes-derived MBH carbonates can act as valuable C1, C2, C3, or C4 synthons, which in situ generated zwitterionic allylic ylide species under Lewis base catalysis [34,35,36,37] or π-allyl metal complexes [38,39,40,41,42,43,44,45], to exhibit universal building blocks for the construction of multifunctional and potentially biologically active ring structures via various [3 + n] [46,47,48,49,50,51,52,53], [2 + n] [54,55,56,57,58], [1 + n] [59,60,61,62,63] annulation reactions. As a result, in the past few years, these annulations of employing the isatin or aldehydes-derived MBH carbonates as substrates have evolved into an extremely vibrant research area and achieved great progress and developments. In sharp contrast, the analogous ninhydrin-derived MBH adducts [46,64,65], including MBH alcohols due to its poor leaving nature, have been less explored compared with classical MBH adducts from isatins or aldehydes. Therefore, it is still of great necessity and importance to develop ninhydrin-derived MBH adducts as substrates to access diverse functional cyclic compounds bearing indane-1,3-dione as promising pharmacophores. Notably, it still is highly desirable to find suitable reaction partners for MBH alcohols [66,67,68], further expanding the chemical space of MBH adducts.

**Scheme 1:**
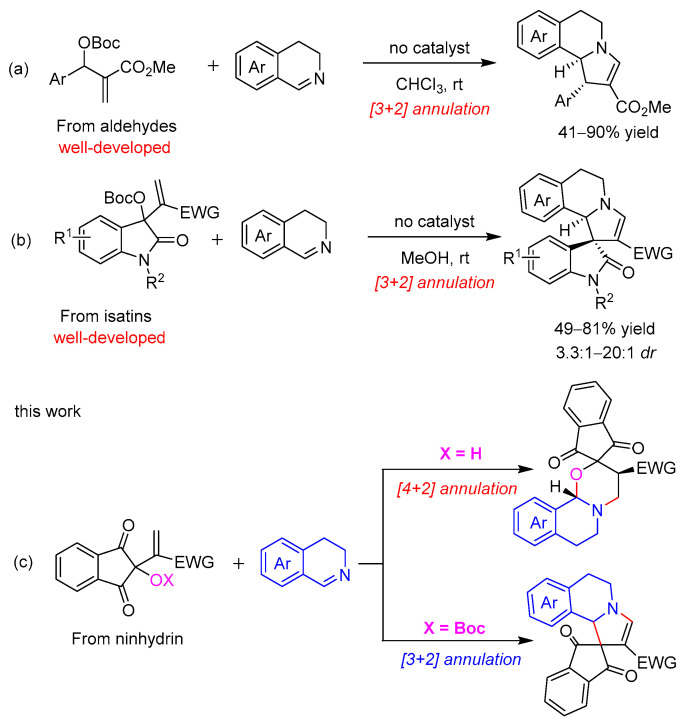
Previous reports of aldehydes or isatins-derived MBH adducts with 3,4-dihydroisoquinolines (**a**,**b**) and our protocol (**c**).

On the other hand, 3,4-dihydroisoquinolines (DHIQ) as an inactivated cyclic imine have been broadly applied as valuable synthons which could react with nucleophilic reagent to fulfill potential active tetrahydroisoquinoline derivatives in organic synthesis [69,70,71,72,73,74,75,76]. In 2017, the Cui and co-workers developed catalyst-free [3 + 2] cyclization of 3,4-dihydroisoquinolines and aldehydes-derived MBH carbonates to provide tetrahydropyrrolo[2,1-*a*]isoquinolines in good yields (Scheme 1a) [77]. It was noteworthy that no desired product was observed between aldehydes-derived MBH alcohol and 3,4-dihydroisoquinoline under standard conditions, because the hydroxyl group was not a good leaving group. Subsequently, their group realized the tetrahydroisoquinoline-fused spirooxindoles containing tetrahydroisoquinoline and spirooxindole frameworks in good yields from 3,4-dihydroisoquinolines with isatins-derived MBH carbonates under the same strategy (Scheme 1b) [78]. Along these lines, the development of efficient synthetic protocols for integrating other kinds of privileged structures into tetrahydroisoquinoline motifs may enhance their biological activities with latent medicinal value. As part of our continued efforts on cycloaddition reactions of MBH adducts as substrates [79,80,81,82], herein, we would like to report this substrate-controlled diversity-oriented synthesis via [4 + 2] and [3 + 2] annulations of ninhydrin-derived MBH adducts with 3,4-dihydroisoquinolines, efficiently furnishing the diversity-oriented synthesis of novel and structurally diverse spiro[indene-2,2′-[1,3]oxazino[2,3-*a*]isoquinoline] and spiro[indene-2,1′-pyrrolo[2,1-*a*]isoquinoline] derivatives with high molecular complexity in excellent diastereo- and regioselectivity (Scheme 1c). This strategy could offer distinct cycloadducts, especially skeletally diverse frameworks, depending on the switchable elaboration of similar starting materials. The incorporation of tetrahydroisoquinoline and indane-1,3-dione moieties and six- or five-membered heterocycles may provide promising opportunities for tuning their diverse pharmacological properties.

## 2. Results

We began our study by screening the model reaction conditions between ninhydrin-derived MBH alcohol **1a** and 3,4-dihydroisoquinoline **2a**. The results are summarized in Table 1. To our delight, the reaction could smoothly proceed via a [4 + 2] cycloaddition reaction and afford the product **3a** in 78% yield with high diastereoselectivity and regioselectivity in the presence of DABCO in CH_2_Cl_2_ at room temperature (entry 1, in Table 1). Then, in order to improve the yield, the reaction was carried out in the presence of different bases, such as TEA, DIPEA, DBU, Na_2_CO_3_, and NaOH (entries 2–6 in Table 1). The results showed that no further improvement in the chemical yield through screening a series of organic and inorganic bases. Subsequently, various solvents were evaluated for the reaction (entries 7–12, in Table 1). Based on the results, with CH_3_CN as the most suitable solvent, the annulation reaction could furnish product **3a** in 85% yield with high >25:1 *dr* (entry 9, in Table 1). The reaction did not effectively offer the product in dioxane or THF (entries 10–11, in Table 1), while only trace amounts of the desired product **3a** were observed even if prolonging the reaction time to 24 h. Other solvents including CHCl_3_, toluene, and EtOAc resulted in inferior yields (entries 6, 8, and 12, in Table 1). On the other hand, increasing the amount of DABCO, the yield of product **3a** has no obvious change (entry 13, in Table 1). Interestingly, as for the MBH adduct, when changed from MBH alcohol **1a** to MBH carbonate **4a** with **2a** in the model reaction, the reaction could switch [3 + 2] annulation, to give product **5a** in 75% yield under the same condition (entry 14, in Table 1). It shows that this switch of [4 + 2] and [3 + 2] annulation is controlled by the MBH adducts. Encouraged by the promising result, we then further optimized the [3 + 2] reaction condition between MBH carbonate **4a** with **2a**. In the absence of DABCO, the [3 + 2] annulation reaction could slightly improve the yield of product **5a** from 75% to 78% (entry 14 vs. entry 15, in Table 1). The subsequent evaluation of solvents (entries 15–20, in Table 1) demonstrated that using CH_2_Cl_2_ as a solvent could obviously improve the yield in a shorter reaction time compared with other solvents.

## 3. Discussion

**Scheme 2:**
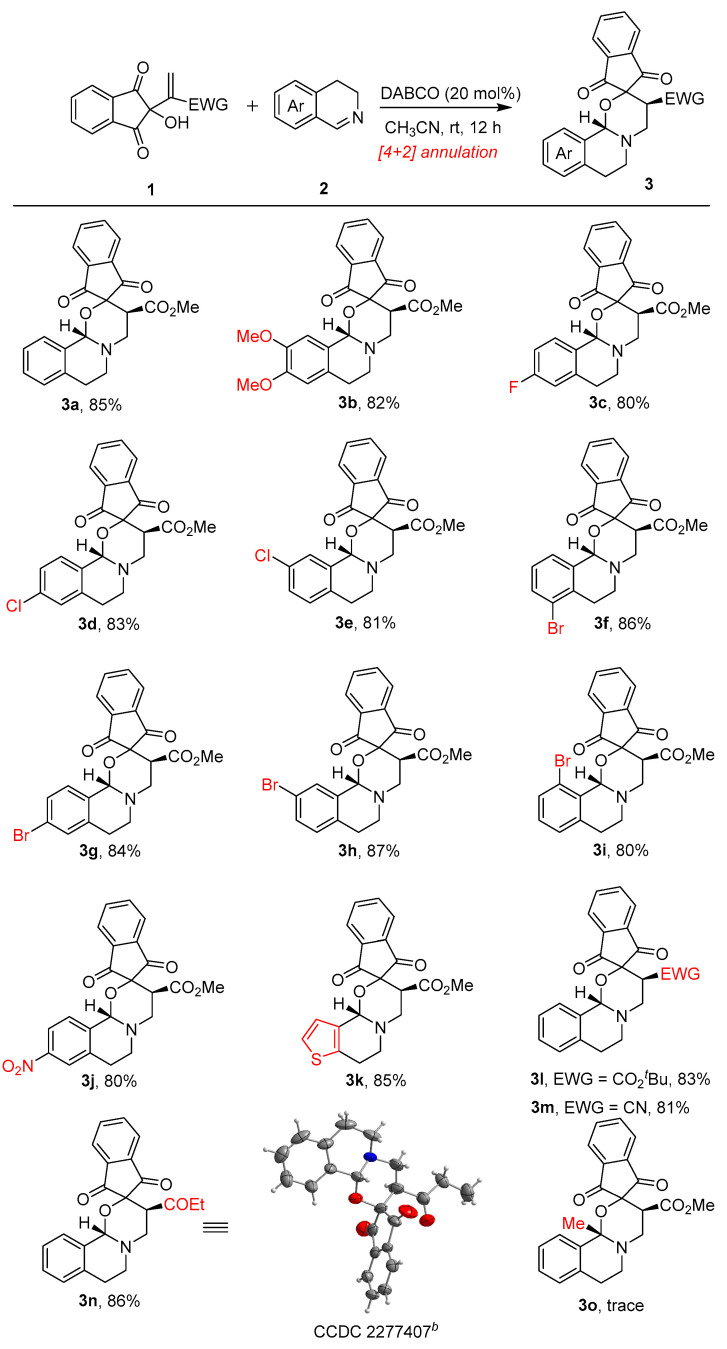
Substrate scopes for [4 + 2] annulations *^a^*. *^a^* Reactions were performed with ninhydrin-derived MBH alcohols **1** (0.11 mmol, 1.1 equiv), 3,4-dihydroisoquinolines **2** (0.1 mmol, 1.0 equiv), DABCO (20 mol%) in CH_3_CN (1.0 mL) at room temperature for 12 h. Isolated yield and *dr* > 25:1 by ^1^H NMR analysis. *^b^* The thermal ellipsoids in the ORTEP diagram (CCDC 2231641) are shown at a 50% probability level.

After establishing the optimized conditions in hand, we then examined the scope of the present [4 + 2] annulation reaction of ninhydrin-derived MBH alcohols with dihydroisoquinolines. Firstly, the effect of substituents on the phenyl ring of dihydroisoquinolines **2** was studied and various dihydroisoquinolines **2** bearing diverse substituents with ninhydrin-derived MBH alcohol **1a** were investigated in the [4 + 2] annulation reaction under optimal conditions. The results were summarized in Scheme 2. The reaction could process smoothly and efficiently transform into the corresponding cycloadducts **3a**–**3k** in 80–87% yields with an excellent diastereomeric ratio (all > 25:1 *dr*). The results clearly demonstrate that the different electronic properties or the substitution pattern variation hardly influenced the reaction efficiency and selectivity, regardless of the different positions or electronic properties of the substituents on the phenyl ring of dihydroisoquinoline moieties. In addition, the reaction could successfully provide the desired product **3k** in 85% yield when it employed heterocyclic imine. On the other hand, we extended the scope of this reaction to investigate the MBH alcohols with other functional groups, such as MBH alcohols bearing a larger steric hindrance of the *tert*-butyl ester group or cyano group or the carbonyl group. These cycloaddition reactions also occurred smoothly to afford the corresponding products **3l**–**3n** in 81–86% yields with an excellent diastereomeric ratio (all > 25:1 *dr*). It is worth noting that all the products could be obtained as a pure diastereomer in >25:1 *dr*. Furthermore, the relative configuration and chemical structure of product **3n** (CCDC 2277407) (see Supplementary Materials) [83] was undoubtedly confirmed by X-ray crystallographic analysis. Unfortunately, the reaction did not effectively provide the desired product **3o** when the cyclic imine bearing a methyl group on the α-position of 3,4-dihydroisoquinoline was applied in this [4 + 2] annulation reaction. The possible reason may be that it is difficult for the construction of quaternary carbon stereocenter when increasing steric hindrance at the α-position of 3,4-dihydroisoquinoline.

**Scheme 3:**
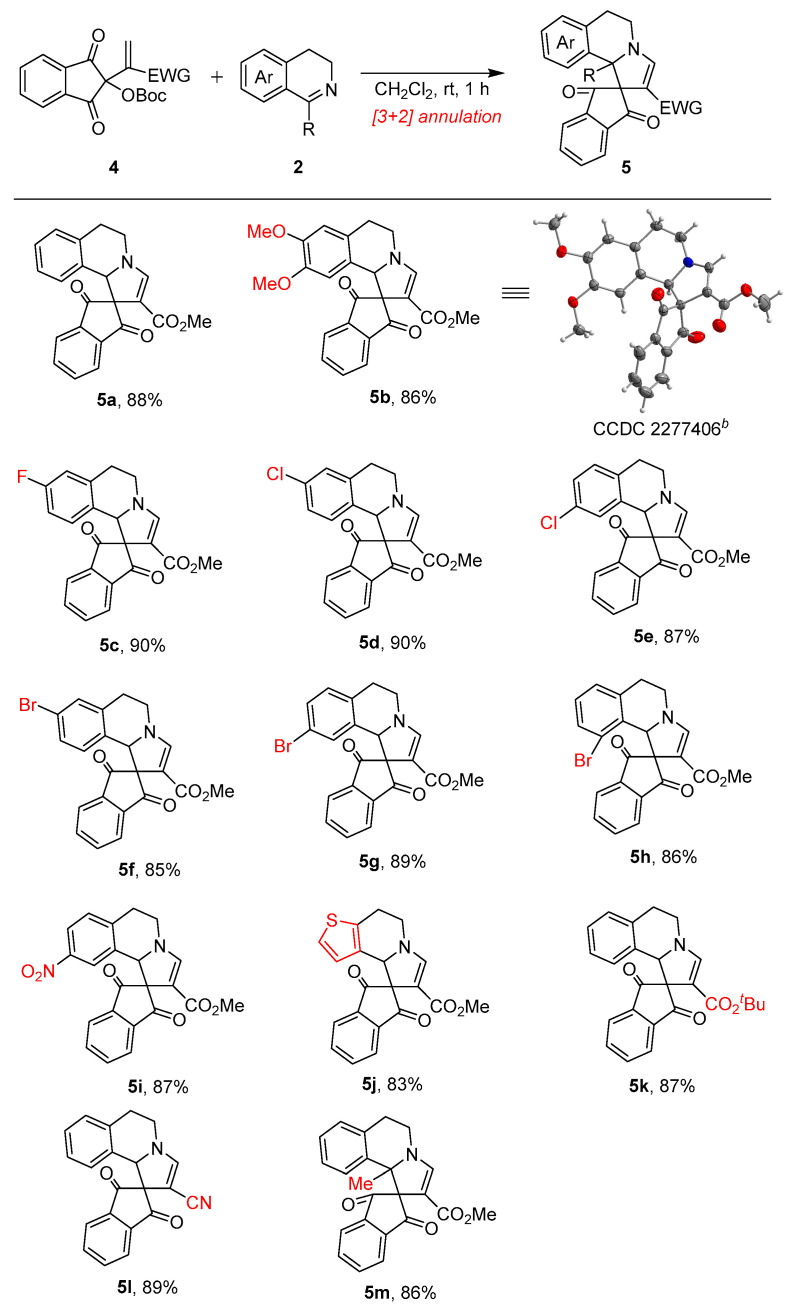
Substrate scopes for [3 + 2] annulations *^a^*. *^a^* Reactions were performed with ninhydrin-derived MBH carbonates **4** (0.11 mmol, 1.1 equiv), 3,4-dihydroisoquinolines **2** (0.1 mmol, 1.0 equiv) in CH_2_Cl_2_ (1.0 mL) at room temperature for 1 h. Isolated yield. *^b^* The thermal ellipsoids in the ORTEP diagram (CCDC 2231641) are shown at a 50% probability level.

Subsequently, the substrate scope of [3 + 2] annulations between ninhydrin-derived MBH carbonates **4** and 3,4-dihydroisoquinolines **2** was also explored. The results were shown in Scheme 3. Under the optimized condition, the [3 + 2] annulation reaction could successfully be carried out when employing 3,4-dihydroisoquinolines featuring different substituents at the C5–C8 positions of the phenyl ring and different substitution patterns, resulting in the corresponding products (**5a**–**5i**) in high yields (85–90%) with excellent regioselectivities. Moreover, the structure of product **5b** (CCDC 2277406) (see Supplementary Materials) [84] was confirmed unambiguously by single-crystal X-ray diffraction analysis. Heterocyclic derived cyclic imine with ninhydrin-derived MBH carbonate **4a** was also successfully tested in the reaction, obtaining the expected product **5j** with slightly lower yield. Furthermore, as for the EWG group on the MBH carbonates, it could be successfully changed from a methyl ester group to a larger steric hindrance of a *tert*-butyl ester group or a cyano group; the reactions were hardly affected, to give the corresponding products **5k** and **5l** in 87% and 89% yields, respectively. It is worth mentioning that the reaction could successfully be extended to 1-methyl-3,4-dihydroisoquinoline, delivering the desired product **5m** containing two vicinal tetrasubstituted carbon stereocenters in 86% yield.

**Scheme 4:**
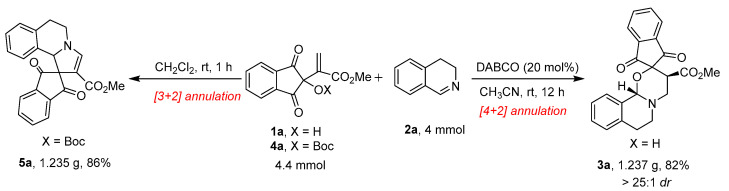
Gram-scale reaction for [4 + 2] cycloadduct **3a** and [3 + 2] cycloadduct **5a**.

Furthermore, in order to exhibit the synthetic practicability of the reaction, a gram-scale experiment of 4.4 mmol **1a** or **4a** with 4 mmol **2a** was conducted under standard conditions (Scheme 4). The larger-scale reaction could smoothly occur, yielding [4 + 2] cycloadduct **3a** and [3 + 2] cycloadduct **5a** in 82% yield with >25:1 *dr* and 86% yield, respectively, similar to the results of the small-scale reaction.

**Scheme 5:**
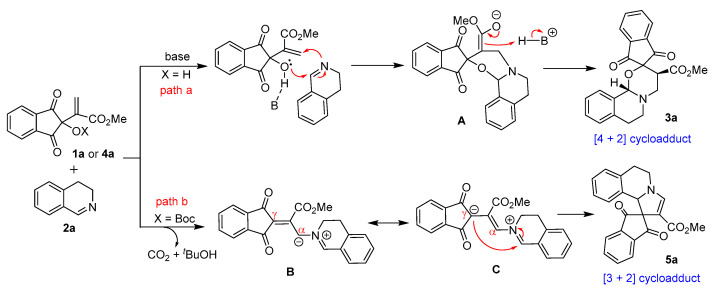
Proposed mechanism to explain the reaction process via these intermediate A–C.

Based on our experimental results and the literature reports, [34,35,36,37,46,47,48,49,50,51,52,53,66,67,68,79,80,81,82] a plausible mechanism is described to explain the reaction process in Scheme 5. Under the basic condition, MBH alcohol **1a** would be activated or deprotonated to form Michael donor with 3,4-dihydroisoquinoline, to produce the intermediate **A** via Michael addition. Subsequently, the intermediate **A** can proceed with intramolecular cyclization to provide the [4 + 2] cycloadduct **3a**. In contrast, the MBH carbonate **4a** as the substrate could be attacked by dihydroisoquinoline **2a** to generate the azomethine ylide **B** or **C** following the release of CO_2_ and *tert-*BuOH. Finally, the ylide **C** followed by an intramolecular ring-closure event through γ-site attacking imine to afford the γ-regioselective [3 + 2] cycloadduct **5a**.

## 4. Materials and Methods

NMR data were obtained for ^1^H at 400 MHz, and for ^13^C at 100 MHz, and ^19^F at 376 MHz. Chemical shifts were reported in ppm from tetramethylsilane with the solvent resonance as the internal standard in CDCl_3_ solution. ESI HRMS was recorded on a Waters SYNAPT G2. Column chromatography was performed on silica gel (200–300 mesh) eluting with ethyl acetate/petroleum ether. TLC was performed on glass-backed silica plates. UV light, I_2_, and solution of potassium permanganate were used to visualize products. All chemicals were used without purification as commercially available unless otherwise noted. Petroleum ether and ethyl acetate were distilled. THF was freshly distilled from sodium/benzophenone. Unless otherwise noted, experiments involving moisture and/or air-sensitive components were performed under a positive pressure of argon in oven-dried glassware equipped with a rubber septum inlet. Dried solvents and liquid reagents were transferred by oven-dried syringes. Ninhydrin-derived MBH adducts **1** and **4** [46,65,66,67] and 3,4-dihydroisoquinolines **2 [71,72,73,74,75,76,77,78]** were prepared according to the literature procedures.

An oven-dried 10 mL flasket charged with a mixture of ninhydrin-derived MBH alcohols **1** (0.11 mmol, 1.1 equiv), 3,4-dihydroisoquinolines **2** (0.1 mmol, 1.0 equiv), DABCO (20 mol%) in CH_3_CN (1.0 mL) was stirred at room temperature for 12 h. After completion, product **3** was obtained by flash chromatography on silica gel (petroleum ether/ethyl acetate = 3:1 to 2:1).

An oven-dried 10 mL flasket charged with a mixture of ninhydrin-derived MBH carbonates **4** (0.11 mmol, 1.1 equiv), 3,4-dihydroisoquinolines **2** (0.1 mmol, 1.0 equiv) in CH_2_Cl_2_ (1.0 mL) was stirred at room temperature for 1 h. After completion, product **5** was obtained by flash chromatography on silica gel (petroleum ether/ethyl acetate = 5:1 to 3:1).

Experimental data of products:

**3a**: Purification by flash chromatography (PE/EA = 3:1 as an eluent, R*_f_* = 0.31) gave a white solid (32.0 mg, 85% yield), mp 138–139 °C, >25:1 *dr*, determined by ^1^H NMR (400 MHz, CDCl_3_) analysis; ^1^H NMR (400 MHz, CDCl_3_) δ 8.09 (d, *J* = 7.6 Hz, 1H), 7.98 (d, *J* = 7.2 Hz, 1H), 7.91–7.82 (m, 2H), 7.18 (t, *J* = 7.2 Hz, 1H), 7.11 (d, *J* = 7.6 Hz, 1H), 7.06 (t, *J* = 7.6 Hz, 1H), 7.00 (d, *J* = 7.6 Hz, 1H), 6.00 (s, 1H), 4.31 (t, *J* = 13.2 Hz, 1H), 3.84 (dd, *J* = 12.4, 5.2 Hz, 1H), 3.71 (td, *J* = 11.6, 3.6 Hz, 1H), 3.57–3.52 (m, 4H), 3.10–3.02 (m, 1H), 2.85–2.81 (m, 2H) ppm. ^13^C{^1^H} NMR (100 MHz, CDCl_3_) δ 198.2, 194.6, 171.4, 141.7, 140.7, 136.0, 135.8, 135.0, 132.4, 128.8, 128.6, 128.5, 125.9, 124.4, 123.9, 84.3, 75.1, 52.2, 49.8, 42.3, 35.1, 29.1 ppm. HRMS (ESI) *m*/*z*: [M + H]^+^ calcd for C_22_H_20_NO_5_ 378.1336, found 378.1319.

**3b**: Purification by flash chromatography (PE/EA = 3:1 as an eluent, R*_f_* = 0.34) gave a white solid (35.8 mg, 82% yield), mp 112–113 °C, >25:1 *dr*, determined by ^1^H NMR (400 MHz, CDCl_3_) analysis; ^1^H NMR (400 MHz, CDCl_3_) δ 8.09 (d, *J* = 7.2 Hz, 1H), 8.00 (d, *J* = 7.2 Hz, 1H), 7.87 (dd, *J* = 16.4, 7.2 Hz, 2H), 6.59 (s, 1H), 6.45 (s, 1H), 5.96 (s, 1H), 4.31 (t, *J* = 13.2 Hz, 1H), 3.86–3.82 (m, 4H), 3.74–3.70 (m, 4H), 3.59–3.51 (m, 4H), 3.05–2.96 (m, 1H), 2.84–2.73 (m, 2H) ppm. ^13^C{^1^H} NMR (100 MHz, CDCl_3_) δ 198.6, 194.7, 171.4, 149.5, 147.4, 141.7, 140.8, 136.0, 135.9, 127.7, 124.5, 124.4, 123.9, 111.3, 110.8, 84.2, 75.1, 56.0, 55.9, 52.2, 49.9, 42.2, 35.2, 28.8 ppm. IR (KBr):3437, 2925, 2839, 1712, 1602, 1518, 1456, 1355, 1323, 1277, 1229, 1139, 1072, 1012, 905, 843, 780, 727, 677, 634, 564, 521 cm^−1^. HRMS (ESI) *m*/*z*: [M + H]^+^ calcd for C_24_H_24_NO_7_ 438.1547, found 438.1540.

**3c**: Purification by flash chromatography (PE/EA = 3:1 as an eluent, R*_f_* = 0.33) gave a white solid (31.6 mg, 80% yield), mp 145–146 °C, >25:1 *dr*, determined by ^1^H NMR (400 MHz, CDCl_3_) analysis; ^1^H NMR (400 MHz, CDCl_3_) δ 8.08 (d, *J* = 7.2 Hz, 1H), 7.99 (d, *J* = 7.2 Hz, 1H), 7.91–7.83 (m, 2H), 7.00–6.96 (m, 1H), 6.82–6.74 (m, 2H), 5.98 (s, 1H), 4.29 (t, *J* = 13.2 Hz, 1H), 3.82 (dd, *J* = 12.4, 5.2 Hz, 1H), 3.67 (t, *J* = 11.2 Hz, 1H), 3.59–3.52 (m, 4H), 3.09–3.00 (m, 1H), 2.82 (d, *J* = 16.4 Hz, 2H) ppm. ^13^C{^1^H} NMR (100 MHz, CDCl_3_) δ 198.2, 194.5, 171.3, 162.6 (d, *J* = 245.9 Hz), 141.7, 140.7, 137.6 (d, *J* = 8.0 Hz), 136.0, 135.9, 130.6 (d, *J* = 8.6 Hz), 128.4 (d, *J* = 2.7 Hz), 124.4, 123.9, 114.8 (d, *J* = 21.0 Hz), 113.3 (d, *J* = 21.7 Hz), 83.8, 75.1, 52.2, 49.8, 41.9, 35.1, 29.2 ppm. ^19^F{^1^H} NMR (376 MHz, CDCl_3_) δ 113.3. HRMS (ESI) *m*/*z*: [M + H]^+^ calcd for C_22_H_19_FNO_5_ 396.1242, found 396.1237.

**3d**: Purification by flash chromatography (PE/EA = 2:1 as an eluent, R*_f_* = 0.32) gave a white solid (34.1 mg, 83% yield), mp 129–130 °C, >25:1 *dr*, determined by ^1^H NMR (400 MHz, CDCl_3_) analysis; ^1^H NMR (400 MHz, CDCl_3_) δ 8.08 (d, *J* = 7.2 Hz, 1H), 7.99 (d, *J* = 7.2 Hz, 1H), 7.92–7.84 (m, 2H), 7.11 (s, 1H), 7.03 (d, *J* = 8.0 Hz, 1H), 6.94 (d, *J* = 8.4 Hz, 1H), 5.97 (s, 1H), 4.28 (t, *J* = 13.2 Hz, 1H), 3.82 (dd, *J* = 12.0, 4.4 Hz, 1H), 3.70–3.64 (m, 1H), 3.59–3.52 (m, 4H), 3.09–2.99 (m, 1H), 2.81 (d, *J* = 13.2 Hz, 2H) ppm. ^13^C{^1^H} NMR (100 MHz, CDCl_3_) δ 198.1, 194.4, 171.2, 141.7, 140.7, 137.0, 136.1, 135.9, 134.3, 130.9, 130.2, 128.3, 126.3, 124.5, 123.9, 83.7, 75.1, 52.2, 49.7, 42.0, 35.2, 29.0 ppm. HRMS (ESI) *m*/*z*: [M + H]^+^ calcd for C_22_H_19_ClNO_5_ 412.0946, found 412.0938.

**3e**: Purification by flash chromatography (PE/EA = 2:1 as an eluent, R*_f_* = 0.36) gave a white solid (33.3 mg, 81% yield), mp 131–132 °C, >25:1 *dr*, determined by ^1^H NMR (400 MHz, CDCl_3_) analysis; ^1^H NMR (400 MHz, CDCl_3_) δ 8.10 (d, *J* = 7.2 Hz, 1H), 8.00 (d, *J* = 7.2 Hz, 1H), 7.94–7.85 (m, 2H), 7.15 (d, *J* = 8.0 Hz, 1H), 7.05 (d, *J* = 8.4 Hz, 1H), 6.99 (s, 1H), 5.94 (s, 1H), 4.27 (t, *J* = 13.2 Hz, 1H), 3.82 (dd, *J* = 12.4, 4.8 Hz, 1H), 3.66 (dd, *J* = 11.6, 7.6 Hz, 1H), 3.56–3.52 (m, 4H), 3.04–2.96 (m, 1H), 2.84–2.77 (m, 2H) ppm. ^13^C{^1^H} NMR (100 MHz, CDCl_3_) δ 198.1, 194.5, 171.2, 141.7, 140.7, 136.1, 136.0, 134.1, 133.6, 131.5, 129.9, 128.8, 128.6, 124.5, 124.0, 83.7, 75.1, 52.3, 49.7, 42.3, 35.3, 28.6 ppm. HRMS (ESI) *m*/*z*: [M + H]^+^ calcd for C_22_H_19_ClNO_5_ 412.0946, found 412.0939.

**3f**: Purification by flash chromatography (PE/EA = 3:1 as an eluent, R*_f_* = 0.36) gave a white solid (39.1 mg, 86% yield), mp 158–159 °C, >25:1 *dr*, determined by ^1^H NMR (400 MHz, CDCl_3_) analysis; ^1^H NMR (400 MHz, CDCl_3_) δ 8.09 (d, *J* = 7.6 Hz, 1H), 8.00 (d, *J* = 7.2 Hz, 1H), 7.92–7.84 (m, 2H), 7.45 (d, *J* = 7.2 Hz, 1H), 6.99–6.93 (m, 2H), 5.98 (s, 1H), 4.28 (t, *J* = 13.2 Hz, 1H), 3.85 (dd, *J* = 12.4, 5.2 Hz, 1H), 3.72–3.65 (m, 1H), 3.58–3.52 (m, 4H), 3.00–2.96 (m, 1H), 2.91–2.83 (m, 2H) ppm. ^13^C{^1^H} NMR (100 MHz, CDCl_3_) δ 198.1, 194.4, 171.2, 141.7, 140.7, 136.1, 135.9, 135.0, 134.7, 132.5, 128.1, 127.3, 124.7, 124.5, 123.9, 83.9, 75.1, 52.3, 49.6, 42.1, 35.1, 30.1 ppm. HRMS (ESI) *m*/*z*: [M + H]^+^ calcd for C_22_H_19_BrNO_5_ 456.0441 (^79^Br) and 458.0421 (^81^Br), found 456.0435, 458.0409.

**3g**: Purification by flash chromatography (PE/EA = 3:1 as an eluent, R*_f_* = 0.35) gave a white solid (38.2 mg, 84% yield), mp 121–122 °C, >25:1 *dr*, determined by ^1^H NMR (400 MHz, CDCl_3_) analysis; ^1^H NMR (400 MHz, CDCl_3_) δ 8.08 (d, *J* = 7.6 Hz, 1H), 7.99 (d, *J* = 7.2 Hz, 1H), 7.92–7.84 (m, 2H), 7.28 (s, 1H), 7.19 (d, *J* = 8.0 Hz, 1H), 6.88 (d, *J* = 8.4 Hz, 1H), 5.95 (s, 1H), 4.28 (t, *J* = 13.2 Hz, 1H), 3.82 (dd, *J* = 12.8, 4.8 Hz, 1H), 3.69–3.64 (m, 1H), 3.56–3.52 (m, 4H), 3.08–2.99 (m, 1H), 2.83–2.79 (m, 2H) ppm. ^13^C{^1^H} NMR (100 MHz, CDCl_3_) δ 198.1, 194.4, 171.2, 141.7, 140.7, 137.4, 136.1, 135.9, 131.5, 131.3, 130.4, 129.2, 124.5, 123.9, 122.5, 83.8, 75.1, 52.3, 49.7, 42.0, 35.2, 28.9 ppm. HRMS (ESI) *m*/*z*: [M + H]^+^ calcd for C_22_H_19_BrNO_5_ 456.0441 (^79^Br) and 458.0421 (^81^Br), found 456.0433, 458.0411.

**3h**: Purification by flash chromatography (PE/EA = 2:1 as an eluent, R*_f_* = 0.34) gave a white solid (39.6 mg, 87% yield), mp 131–132 °C, >25:1 *dr*, determined by ^1^H NMR (400 MHz, CDCl_3_) analysis; ^1^H NMR (400 MHz, CDCl_3_) δ 8.10 (d, *J* = 6.8 Hz, 1H), 8.01 (d, *J* = 6.8 Hz, 1H), 7.92–7.87 (m, 2H), 7.30 (d, *J* = 8.0 Hz, 1H), 7.14 (s, 1H), 6.99 (d, *J* = 7.6 Hz, 1H), 5.94 (s, 1H), 4.27 (t, *J* = 13.2 Hz, 1H), 3.82 (dd, *J* = 12.0, 4.0 Hz, 1H), 3.65 (t, *J* = 10.4 Hz, 1H), 3.56–3.52 (m, 4H), 3.00–2.93 (m, 1H), 2.79 (d, *J* = 16.4 Hz, 2H) ppm. ^13^C{^1^H} NMR (100 MHz, CDCl_3_) δ 198.1, 194.5, 171.2, 141.7, 140.7, 136.2, 136.0, 134.5, 134.1, 131.62, 131.56, 130.2, 124.5, 124.0, 119.4, 83.6, 75.1, 52.3, 49.7, 42.2, 35.3, 28.7 ppm. HRMS (ESI) *m*/*z*: [M + H]^+^ calcd for C_22_H_19_BrNO_5_ 456.0441 (^79^Br) and 458.0421 (^81^Br), found 456.0437, 458.0414.

**3i**: Purification by flash chromatography (PE/EA = 3:1 as an eluent, R*_f_* = 0.37) gave a white solid (36.4 mg, 80% yield), mp 154–155 °C, >25:1 *dr*, determined by ^1^H NMR (400 MHz, CDCl_3_) analysis; ^1^H NMR (400 MHz, CDCl_3_) δ 8.07 (d, *J* = 7.2 Hz, 1H), 7.98 (d, *J* = 7.2 Hz, 1H), 7.89–7.81 (m, 2H), 7.26 (d, *J* = 5.2 Hz, 1H), 7.06–7.02 (m, 2H), 6.16 (s, 1H), 4.41 (t, *J* = 13.2 Hz, 1H), 3.87 (dd, *J* = 12.4, 4.8 Hz, 1H), 3.73 (dd, *J* = 16.0, 6.8 Hz, 1H), 3.60–3.54 (m, 4H), 3.12–3.03 (m, 1H), 2.84–2.80 (m, 2H) ppm. ^13^C{^1^H} NMR (100 MHz, CDCl_3_) δ 197.0, 194.2, 171.3, 142.2, 140.6, 137.9, 136.1, 135.6, 131.7, 130.7, 129.9, 127.8, 124.4, 123.7, 83.5, 75.2, 52.2, 49.9, 41.6, 34.3, 29.4 ppm. HRMS (ESI) *m*/*z*: [M + H]^+^ calcd for C_22_H_19_BrNO_5_ 456.0441 (^79^Br) and 458.0421 (^81^Br), found 456.0436, 458.0413.

**3j**: Purification by flash chromatography (PE/EA = 2:1 as an eluent, R*_f_* = 0.33) gave a yellow solid (33.8 mg, 80% yield), mp 159–160 °C, >25:1 *dr*, determined by ^1^H NMR (400 MHz, CDCl_3_) analysis; ^1^H NMR (400 MHz, CDCl_3_) δ 8.13 (d, *J* = 7.2 Hz, 1H), 8.06–8.00 (m, 2H), 7.95 (t, *J* = 7.2 Hz, 1H), 7.90–7.87 (m, 2H), 7.29 (d, *J* = 8.4 Hz, 1H), 6.08 (s, 1H), 4.30 (t, *J* = 13.2 Hz, 1H), 3.83 (dd, *J* = 12.4, 5.2 Hz, 1H), 3.72–3.66 (m, 1H), 3.59–3.52 (m, 4H), 3.16–3.08 (m, 1H), 2.98–2.87 (m, 2H) ppm. ^13^C{^1^H} NMR (100 MHz, CDCl_3_) δ 198.0, 194.4, 170.9, 146.3, 143.1, 141.7, 140.6, 136.3, 136.1, 133.9, 129.6, 124.5, 124.22, 124.18, 123.4, 83.4, 75.0, 52.3, 49.6, 41.8, 35.4, 29.4 ppm. HRMS (ESI) *m*/*z*: [M + H]^+^ calcd for C_22_H_19_N_2_O_7_ 423.1187, found 423.1180.

**3k**: Purification by flash chromatography (PE/EA = 3:1 as an eluent, R*_f_* = 0.34) gave a pale yellow solid (32.6 mg, 85% yield), mp 114–115 °C, >25:1 *dr*, determined by ^1^H NMR (400 MHz, CDCl_3_) analysis; ^1^H NMR (400 MHz, CDCl_3_) δ 8.07 (d, *J* = 6.8 Hz, 1H), 7.99 (d, *J* = 6.8 Hz, 1H), 7.89–7.85 (m, 2H), 6.99 (d, *J* = 4.4 Hz, 1H), 6.68 (d, *J* = 4.8 Hz, 1H), 5.97 (s, 1H), 4.30 (t, *J* = 13.2 Hz, 1H), 3.81 (dd, *J* = 12.0, 4.4 Hz, 1H), 3.73–3.69 (m, 1H), 3.59–3.51 (m, 4H), 3.04–2.97 (m, 1H), 2.90 (d, *J* = 12.4 Hz, 2H) ppm. ^13^C{^1^H} NMR (100 MHz, CDCl_3_) δ 198.1, 194.6, 171.2, 141.7, 140.7, 138.1, 136.0, 135.9, 132.6, 125.7, 124.4, 123.9, 123.2, 81.4, 74.8, 52.2, 49.6, 43.1, 35.7, 25.6 ppm. HRMS (ESI) *m*/*z*: [M + H]^+^ calcd for C_20_H_18_NO_5_S 384.0900, found 384.0893.

**3l**: Purification by flash chromatography (PE/EA = 3:1 as an eluent, R*_f_* = 0.33) gave a white solid (34.8 mg, 83% yield), mp 122–125 °C, >25:1 *dr*, determined by ^1^H NMR (400 MHz, CDCl_3_) analysis; ^1^H NMR (400 MHz, CDCl_3_) δ 8.09 (d, *J* = 7.6 Hz, 1H), 7.99 (d, *J* = 7.2 Hz, 1H), 7.91–7.82 (m, 2H), 7.17 (t, *J* = 7.2 Hz, 1H), 7.10 (d, *J* = 7.6 Hz, 1H), 7.05 (t, *J* = 7.6 Hz, 1H), 6.99 (d, *J* = 7.2 Hz, 1H), 5.93 (s, 1H), 4.24 (t, *J* = 12.8 Hz, 1H), 3.74–3.65 (m, 2H), 3.50 (dd, *J* = 14.0, 5.2 Hz, 1H), 3.09–3.01 (m, 1H), 2.84–2.81 (m, 2H), 1.08 (s, 9H) ppm. ^13^C{^1^H} NMR (100 MHz, CDCl_3_) δ 198.1, 194.4, 169.3, 141.8, 141.0, 135.9, 135.8, 135.1, 132.5, 128.8, 128.52, 128.50, 125.9, 124.5, 123.9, 84.4, 82.7, 75.4, 49.9, 42.4, 34.9, 29.1, 27.5 ppm. HRMS (ESI) *m*/*z*: [M + H]^+^ calcd for C_25_H_26_NO_5_ 420.1805, found 420.1800.

**3m**: Purification by flash chromatography (PE/EA = 3:1 as an eluent, R*_f_* = 0.34) gave a white solid (27.9 mg, 81% yield), mp 117–118 °C, >25:1 *dr*, determined by ^1^H NMR (400 MHz, CDCl_3_) analysis; ^1^H NMR (400 MHz, CDCl_3_) δ 8.14 (d, *J* = 7.6 Hz, 1H), 8.04 (d, *J* = 7.2 Hz, 1H), 7.97 (dd, *J* = 16.4, 7.2 Hz, 2H), 7.22 (t, *J* = 7.2 Hz, 1H), 7.11 (dd, *J* = 15.2, 7.6 Hz, 2H), 7.05 (d, *J* = 7.6 Hz, 1H), 6.28 (s, 1H), 4.43 (t, *J* = 13.2 Hz, 1H), 3.68 (dd, *J* = 12.0, 5.6 Hz, 2H), 3.51 (dd, *J* = 14.0, 4.4 Hz, 1H), 3.11–3.02 (m, 1H), 2.83 (d, *J* = 12.0 Hz, 2H) ppm. ^13^C{^1^H} NMR (100 MHz, CDCl_3_) δ 197.2, 192.9, 141.2, 140.4, 137.3, 137.1, 134.8, 131.8, 128.9, 128.8, 128.6, 126.1, 124.8, 124.4, 116.3, 84.3, 75.5, 50.3, 42.2, 29.0, 21.3 ppm. HRMS (ESI) *m*/*z*: [M + H]^+^ calcd for C_21_H_17_N_2_O_3_ 345.1234, found 345.1227.

**3n**: Purification by flash chromatography (PE/EA = 2:1 as an eluent, R*_f_* = 0.32) gave a white solid (32.3 mg, 86% yield), mp 127–128 °C, >25:1 *dr*, determined by ^1^H NMR (400 MHz, CDCl_3_) analysis; ^1^H NMR (400 MHz, CDCl_3_) δ 8.09 (d, *J* = 7.6 Hz, 1H), 7.94 (d, *J* = 7.6 Hz, 1H), 7.88 (t, *J* = 7.2 Hz, 1H), 7.82 (t, *J* = 7.6 Hz, 1H), 7.18 (t, *J* = 7.2 Hz, 1H), 7.11 (d, *J* = 7.2 Hz, 1H), 7.06 (t, *J* = 7.6 Hz, 1H), 7.00 (d, *J* = 7.6 Hz, 1H), 5.98 (s, 1H), 4.32 (t, *J* = 13.2 Hz, 1H), 3.89 (dd, *J* = 12.4, 4.8 Hz, 1H), 3.75 (td, *J* = 11.2, 3.6 Hz, 1H), 3.59 (dd, *J* = 13.6, 4.8 Hz, 1H), 3.12–3.04 (m, 1H), 2.88–2.84 (m, 2H), 2.54–2.36 (m, 2H), 0.93 (t, *J* = 6.8 Hz, 3H) ppm. ^13^C{^1^H} NMR (100 MHz, CDCl_3_) δ 209.9, 198.1, 195.0, 141.9, 140.4, 135.9, 135.5, 134.9, 132.5, 128.8, 128.8, 128.6, 128.5, 126.0, 124.3, 123.9, 84.1, 75.4, 50.4, 44.6, 42.6, 33.3, 29.2, 7.0 ppm. HRMS (ESI) *m*/*z*: [M + H]^+^ calcd for C_23_H_22_NO_4_ 376.1543, found 376.1537.

**5a**: Purification by flash chromatography (PE/EA = 3:1 as an eluent, R*_f_* = 0.32) gave a white solid (31.6 mg, 88% yield), mp 116–117 °C, ^1^H NMR (400 MHz, CDCl_3_) δ 8.12 (d, *J* = 7.6 Hz, 1H), 7.86 (t, *J* = 7.2 Hz, 1H), 7.79 (t, *J* = 7.6 Hz, 1H), 7.72 (d, *J* = 7.2 Hz, 1H), 7.47 (s, 1H), 7.13–7.05 (m, 2H), 6.80 (t, *J* = 7.2 Hz, 1H), 6.22 (d, *J* = 7.6 Hz, 1H), 5.67 (s, 1H), 3.77 (dd, *J* = 12.8, 4.4 Hz, 1H), 3.43–3.34 (m, 4H), 3.24–3.16 (m, 1H), 2.75 (d, *J* = 15.2 Hz, 1H) ppm. ^13^C{^1^H} NMR (100 MHz, CDCl_3_) δ 202.9, 198.9, 164.6, 151.9, 142.9, 142.1, 135.8, 135.4, 135.3, 131.4, 129.5, 127.5, 126.5, 125.1, 123.3, 123.2, 102.7, 68.1, 67.3, 50.6, 45.0, 30.2 ppm. HRMS (ESI) *m*/*z*: [M + H]^+^ calcd for C_22_H_18_NO_4_ 360.1230, found 360.1226.

**5b**: Purification by flash chromatography (PE/EA = 5:1 as an eluent, R*_f_* = 0.35) gave a white solid (36.0 mg, 86% yield), mp 125–126 °C, ^1^H NMR (400 MHz, CDCl_3_) δ 8.13 (d, *J* = 7.6 Hz, 1H), 7.85 (t, *J* = 7.2 Hz, 1H), 7.79 (t, *J* = 7.6 Hz, 1H), 7.74 (d, *J* = 7.6 Hz, 1H), 7.49 (s, 1H), 6.57 (s, 1H), 5.58 (d, *J* = 4.4 Hz, 2H), 3.80 (s, 3H), 3.75 (dd, *J* = 13.2, 5.2 Hz, 1H), 3.45 (s, 3H), 3.36 (td, *J* = 12.4, 2.8 Hz, 1H), 3.14–3.06 (m, 1H), 3.02 (s, 3H), 2.65 (d, *J* = 14.8 Hz, 1H) ppm. ^13^C{^1^H} NMR (100 MHz, CDCl_3_) δ 203.2, 198.8, 164.7, 151.9, 148.0, 147.4, 142.9, 142.2, 135.9, 135.3, 127.5, 123.4, 123.2, 122.6, 111.8, 107.8, 102.1, 68.4, 67.4, 55.7, 54.9, 50.7, 44.9, 29.6 ppm. HRMS (ESI) *m*/*z*: [M + H]^+^ calcd for C_24_H_22_NO_6_ 420.1442, found 420.1433.

**5c**: Purification by flash chromatography (PE/EA = 3:1 as an eluent, R*_f_* = 0.32) gave a white solid (33.9 mg, 90% yield), mp 109–110 °C, ^1^H NMR (400 MHz, CDCl_3_) δ 8.11 (d, *J* = 7.6 Hz, 1H), 7.87 (t, *J* = 7.2 Hz, 1H), 7.80 (t, *J* = 7.6 Hz, 1H), 7.73 (d, *J* = 7.6 Hz, 1H), 7.46 (s, 1H), 6.84 (d, *J* = 9.2 Hz, 1H), 6.52 (t, *J* = 8.4 Hz, 1H), 6.22–6.18 (m, 1H), 5.63 (s, 1H), 3.77 (dd, *J* = 12.8, 4.8 Hz, 1H), 3.43 (s, 3H), 3.35 (t, *J* = 12.4 Hz, 1H), 3.23–3.15 (m, 1H), 2.73 (d, *J* = 15.2 Hz, 1H) ppm. ^13^C{^1^H} NMR (100 MHz, CDCl_3_) δ 202.7, 198.8, 164.5, 161.5 (d, *J* = 245.9 Hz), 151.8, 142.9, 141.9, 137.9 (d, *J* = 7.6 Hz), 136.0, 135.6, 127.2 (d, *J* = 3.1 Hz), 126.7 (d, *J* = 8.4 Hz), 123.3, 123.2, 116.1 (d, *J* = 21.1 Hz), 113.8 (d, *J* = 21.9 Hz), 103.1, 67.6, 67.3, 50.7, 44.7, 30.3 ppm. ^19^F{^1^H} NMR (376 MHz, CDCl_3_) δ 114.1. HRMS (ESI) *m*/*z*: [M + H]^+^ calcd for C_22_H_17_FNO_4_ 378.1136, found 378.1130.

**5d**: Purification by flash chromatography (PE/EA = 4:1 as an eluent, R*_f_* = 0.35) gave a white solid (35.4 mg, 90% yield), mp 137–138 °C, ^1^H NMR (400 MHz, CDCl_3_) δ 8.11 (d, *J* = 7.6 Hz, 1H), 7.87 (t, *J* = 7.6 Hz, 1H), 7.81 (t, *J* = 7.6 Hz, 1H), 7.75 (d, *J* = 7.2 Hz, 1H), 7.45 (s, 1H), 7.13 (s, 1H), 6.79 (d, *J* = 8.0 Hz, 1H), 6.16 (d, *J* = 8.0 Hz, 1H), 5.62 (s, 1H), 3.77 (dd, *J* = 12.8, 4.4 Hz, 1H), 3.43 (s, 3H), 3.34 (t, *J* = 12.4 Hz, 1H), 3.22–3.14 (m, 1H), 2.73 (d, *J* = 15.6 Hz, 1H) ppm. ^13^C{^1^H} NMR (100 MHz, CDCl_3_) δ 202.5, 198.7, 164.5, 151.7, 142.9, 141.9, 137.3, 136.0, 135.6, 133.2, 130.0, 129.4, 126.8, 126.4, 123.33, 123.29, 103.2, 67.5, 67.2, 50.7, 44.7, 30.0 ppm. HRMS (ESI) *m*/*z*: [M + H]^+^ calcd for C_22_H_17_ClNO_4_ 394.0841, found 394.0834.

**5e**: Purification by flash chromatography (PE/EA = 3:1 as an eluent, R*_f_* = 0.32) gave a white solid (34.2 mg, 87% yield), mp 131−132 °C, ^1^H NMR (400 MHz, CDCl_3_) δ 8.13 (d, *J* = 7.6 Hz, 1H), 7.89 (t, *J* = 7.6 Hz, 1H), 7.83 (t, *J* = 7.2 Hz, 1H), 7.76 (d, *J* = 7.6 Hz, 1H), 7.45 (s, 1H), 7.07–7.02 (m, 2H), 6.17 (s, 1H), 5.58 (s, 1H), 3.77 (dd, *J* = 12.8, 4.8 Hz, 1H), 3.44 (s, 3H), 3.33 (t, *J* = 10.8 Hz, 1H), 3.17–3.09 (m, 1H), 2.73 (d, *J* = 15.6 Hz, 1H) ppm. ^13^C{^1^H} NMR (100 MHz, CDCl_3_) δ 202.4, 198.6, 164.5, 151.8, 142.8, 142.0, 136.1, 135.7, 133.8, 133.2, 132.2, 130.7, 127.7, 125.2, 123.4, 123.3, 103.1, 67.6, 67.3, 50.7, 44.8, 29.6 ppm. HRMS (ESI) *m*/*z*: [M + H]^+^ calcd for C_22_H_17_ClNO_4_ 394.0841, found 394.0841.

**5f**: Purification by flash chromatography (PE/EA = 4:1 as an eluent, R*_f_* = 0.33) gave a white solid (37.1 mg, 85% yield), mp 128–129 °C, ^1^H NMR (400 MHz, CDCl_3_) δ 8.11 (d, *J* = 7.6 Hz, 1H), 7.87 (t, *J* = 7.2 Hz, 1H), 7.81 (t, *J* = 7.2 Hz, 1H), 7.75 (d, *J* = 7.2 Hz, 1H), 7.45 (s, 1H), 7.29 (s, 1H), 6.94 (d, *J* = 8.0 Hz, 1H), 6.10 (d, *J* = 8.0 Hz, 1H), 5.60 (s, 1H), 3.77 (dd, *J* = 12.8, 4.0 Hz, 1H), 3.43 (s, 3H), 3.34 (t, *J* = 12.4 Hz, 1H), 3.18 (t, *J* = 10.8 Hz, 1H), 2.72 (d, *J* = 15.2 Hz, 1H) ppm. ^13^C{^1^H} NMR (100 MHz, CDCl_3_) δ 202.5, 198.7, 164.5, 151.7, 142.9, 141.9, 137.6, 136.0, 135.6, 132.4, 130.5, 129.7, 126.6, 123.33, 123.32, 121.3, 103.2, 67.6, 67.1, 50.7, 44.7, 29.9 ppm. HRMS (ESI) *m*/*z*: [M + H]^+^ calcd for C_22_H_17_BrNO_4_ 438.0335 (^79^Br) and 440.0315 (^81^Br), found 438.0327, 440.0305.

**5g**: Purification by flash chromatography (PE/EA = 5:1 as an eluent, R*_f_* = 0.36) gave a white solid (38.9 mg, 89% yield), mp 133–134 °C, ^1^H NMR (400 MHz, CDCl_3_) δ 8.13 (d, *J* = 7.2 Hz, 1H), 7.89 (t, *J* = 7.2 Hz, 1H), 7.83 (t, *J* = 7.2 Hz, 1H), 7.76 (d, *J* = 7.2 Hz, 1H), 7.45 (s, 1H), 7.18 (d, *J* = 8.4 Hz, 1H), 6.99 (d, *J* = 8.0 Hz, 1H), 6.31 (s, 1H), 5.57 (s, 1H), 3.77 (dd, *J* = 12.8, 4.8 Hz, 1H), 3.44 (s, 3H), 3.36–3.29 (m, 1H), 3.13–3.05 (m, 1H), 2.71 (d, *J* = 15.6 Hz, 1H) ppm. ^13^C{^1^H} NMR (100 MHz, CDCl_3_) δ 202.4, 198.6, 164.5, 151.8, 142.7, 142.0, 136.1, 135.7, 134.3, 133.6, 131.0, 130.5, 128.3, 123.4, 123.3, 119.9, 103.1, 67.5, 67.4, 50.7, 44.7, 29.6 ppm. HRMS (ESI) *m*/*z*: [M + H]^+^ calcd for C_22_H_17_BrNO_4_ 438.0335 (^79^Br) and 440.0315 (^81^Br), found 438.0330, 440.0307.

**5h**: Purification by flash chromatography (PE/EA = 3:1 as an eluent, R*_f_* = 0.32) gave a white solid (37.6 mg, 86% yield), mp 130–131 °C, ^1^H NMR (400 MHz, CDCl_3_) δ 8.01 (d, *J* = 7.6 Hz, 1H), 7.80 (t, *J* = 7.2 Hz, 1H), 7.75 (t, *J* = 7.2 Hz, 1H), 7.71 (d, *J* = 7.2 Hz, 1H), 7.39 (s, 1H), 7.18 (d, *J* = 8.0 Hz, 1H), 7.12 (d, *J* = 7.2 Hz, 1H), 7.01 (t, *J* = 8.0 Hz, 1H), 5.90 (s, 1H), 3.74 (d, *J* = 12.0 Hz, 1H), 3.41 (s, 3H), 3.21 (t, *J* = 14.4 Hz, 2H), 2.71 (d, *J* = 13.6 Hz, 1H) ppm. ^13^C{^1^H} NMR (100 MHz, CDCl_3_) δ 202.4, 197.9, 164.1, 153.2, 143.6, 140.9, 140.4, 135.3, 132.0, 131.4, 128.7, 128.5, 122.9, 122.8, 122.7, 106.7, 68.6, 67.5, 50.7, 46.2, 30.9 ppm. HRMS (ESI) *m*/*z*: [M + H]^+^ calcd for C_22_H_17_BrNO_4_ 438.0335 (^79^Br) and 440.0315 (^81^Br), found 438.0330, 440.0308.

**5i**: Purification by flash chromatography (PE/EA = 4:1 as an eluent, R*_f_* = 0.34) gave a white solid (35.1 mg, 87% yield), mp 124–125 °C, ^1^H NMR (400 MHz, CDCl_3_) δ 8.18 (d, *J* = 7.6 Hz, 1H), 7.92 (t, *J* = 8.0 Hz, 2H), 7.82 (t, *J* = 7.2 Hz, 1H), 7.70 (d, *J* = 7.6 Hz, 1H), 7.47 (s, 1H), 7.31 (d, *J* = 8.4 Hz, 1H), 7.08 (s, 1H), 5.67 (s, 1H), 3.84 (dd, *J* = 12.8, 4.8 Hz, 1H), 3.45 (s, 3H), 3.39 (t, *J* = 12.8 Hz, 1H), 3.29–3.21 (m, 1H), 2.90 (d, *J* = 15.6 Hz, 1H) ppm. ^13^C{^1^H} NMR (100 MHz, CDCl_3_) δ 201.9, 198.6, 164.3, 151.6, 146.2, 143.0, 142.7, 141.8, 136.4, 136.1, 133.3, 130.6, 123.7, 123.3, 122.3, 120.3, 103.8, 67.6, 67.3, 50.8, 44.3, 30.3 ppm. HRMS (ESI) *m*/*z*: [M + H]^+^ calcd for C_22_H_17_N_2_O_6_ 405.1081, found 405.1077.

**5j**: Purification by flash chromatography (PE/EA = 5:1 as an eluent, R*_f_* = 0.33) gave a white solid (30.3 mg, 83% yield), mp 132–133 °C, ^1^H NMR (400 MHz, CDCl_3_) δ 8.10 (d, *J* = 6.8 Hz, 1H), 7.87–7.84 (m, 3H), 7.51 (s, 1H), 6.89 (d, *J* = 5.2 Hz, 1H), 5.86 (d, *J* = 5.2 Hz, 1H), 5.49 (s, 1H), 3.84 (dd, *J* = 12.8, 5.2 Hz, 1H), 3.44–3.37 (m, 4H), 3.15–3.07 (m, 1H), 2.87 (d, *J* = 15.6 Hz, 1H) ppm. ^13^C{^1^H} NMR (100 MHz, CDCl_3_) δ 202.5, 198.5, 164.7, 151.6, 142.4, 141.9, 136.0, 135.5, 135.3, 129.7, 123.9, 123.32, 123.25, 102.1, 67.0, 65.9, 50.6, 44.8, 25.7 ppm. HRMS (ESI) *m*/*z*: [M + H]^+^ calcd for C_20_H_16_NO_4_S 366.0795, found 366.0792.

**5k**: Purification by flash chromatography (PE/EA = 4:1 as an eluent, R*_f_* = 0.34) gave a white solid (34.9 mg, 87% yield), mp 119–120 °C, ^1^H NMR (400 MHz, CDCl_3_) δ 8.11 (d, *J* = 7.6 Hz, 1H), 7.84 (t, *J* = 7.2 Hz, 1H), 7.78 (t, *J* = 7.6 Hz, 1H), 7.72 (d, *J* = 7.6 Hz, 1H), 7.46 (s, 1H), 7.11 (d, *J* = 7.6 Hz, 1H), 7.05 (t, *J* = 7.2 Hz, 1H), 6.78 (t, *J* = 7.6 Hz, 1H), 6.20 (d, *J* = 7.6 Hz, 1H), 5.65 (s, 1H), 3.76 (dd, *J* = 12.4, 4.9 Hz, 1H), 3.33 (td, *J* = 12.4, 2.4 Hz, 1H), 3.22–3.14 (m, 1H), 2.73 (d, *J* = 15.2 Hz, 1H), 1.04 (s, 9H) ppm. ^13^C{^1^H} NMR (100 MHz, CDCl_3_) δ 203.2, 198.8, 163.4, 152.2, 143.0, 142.3, 135.8, 135.6, 135.3, 131.6, 129.4, 127.4, 126.3, 125.2, 123.3, 123.1, 104.6, 79.3, 68.6, 67.4, 45.0, 30.1, 27.9 ppm. HRMS (ESI) *m*/*z*: [M + H]^+^ calcd for C_25_H_24_NO_4_ 402.1700, found 402.1693.

**5l**: Purification by flash chromatography (PE/EA = 3:1 as an eluent, R*_f_* = 0.31) gave a yellow solid (29.0 mg, 89% yield), mp 126–127 °C, ^1^H NMR (400 MHz, CDCl_3_) δ 8.14 (d, *J* = 7.6 Hz, 1H), 7.92 (t, *J* = 7.2 Hz, 1H), 7.86 (t, *J* = 7.6 Hz, 1H), 7.79 (d, *J* = 7.6 Hz, 1H), 7.21 (s, 1H), 7.15–7.08 (m, 2H), 6.85 (t, *J* = 7.6 Hz, 1H), 6.26 (d, *J* = 7.6 Hz, 1H), 5.73 (s, 1H), 3.79 (dd, *J* = 12.8, 5.2 Hz, 1H), 3.42–3.35 (m, 1H), 3.25–3.17 (m, 1H), 2.78 (d, *J* = 15.2 Hz, 1H) ppm. ^13^C{^1^H} NMR (100 MHz, CDCl_3_) δ 199.8, 196.2, 153.4, 142.1, 141.9, 136.8, 136.3, 135.2, 130.8, 129.5, 127.7, 126.8, 125.0, 124.0, 123.9, 116.4, 78.4, 67.3, 66.6, 45.0, 30.0 ppm. HRMS (ESI) *m*/*z*: [M + H]^+^ calcd for C_21_H_15_N_2_O_2_ 327.1128, found 327.1122.

**5m**: Purification by flash chromatography (PE/EA = 3:1 as an eluent, R*_f_* = 0.32) gave a yellow solid (32.1 mg, 86% yield), mp 117–118 °C, ^1^H NMR (400 MHz, CDCl_3_) δ 8.10 (d, *J* = 7.6 Hz, 1H), 7.82 (t, *J* = 7.2 Hz, 1H), 7.72 (t, *J* = 7.6 Hz, 1H), 7.57 (d, *J* = 7.6 Hz, 1H), 7.46 (s, 1H), 7.09 (d, *J* = 7.6 Hz, 1H), 7.03 (t, *J* = 7.6 Hz, 1H), 6.71 (t, *J* = 7.6 Hz, 1H), 6.20 (d, *J* = 8.0 Hz, 1H), 3.70 (dd, *J* = 13.2, 5.2 Hz, 1H), 3.46–3.39 (m, 4H), 3.23–3.14 (m, 1H), 2.74 (d, *J* = 15.2 Hz, 1H), 1.74 (s, 3H) ppm. ^13^C NMR (100 MHz, CDCl_3_) δ 199.6, 199.5, 164.9, 150.5, 143.1, 142.2, 136.8, 135.5, 135.3, 134.5, 129.6, 127.2, 126.0, 125.7, 123.1, 123.0, 100.5, 72.0, 69.1, 50.6, 42.5, 30.7, 25.4 ppm. HRMS (ESI) *m*/*z*: [M + H]^+^ calcd for C_23_H_20_NO_4_ 374.1387, found 374.1379.

## 5. Conclusions and Future Perspective

In summary, we have developed a substrate-controlled diversity-oriented synthesis via [4 + 2] and [3 + 2] annulation reaction to afford regioselective synthesis of a broad range of bioinspired novel spiro[indene-2,2′-[1,3]oxazino[2,3-*a*]isoquinoline] and spiro[indene-2,1′-pyrrolo[2,1-*a*]isoquinoline] derivatives containing tetrahydroisoquinoline motif and indane-1,3-dione motif, relying on the ninhydrin-derived MBH adducts encountered. The ninhydrin-derived MBH alcohols serves as a four-atom synthon in [4 + 2] annulation with 3,4-dihydroisoquinolines to furnish the spiro[indene-2,2′-[1,3]oxazino[2,3-*a*]isoquinoline] in good yields (up to 87%) with excellent diastereoselectivity (>25:1 *dr*), while its carbonates serve as a three-atom synthon in [3 + 2] annulation with the same reaction partners under a similar reaction condition, which leads to γ-regioselective formation of spiro[indene-2,1′-pyrrolo[2,1-*a*]isoquinoline] derivatives in high yields (up to 90%). In addition, a proposed mechanism for the switchable reactions is provided. This method exhibits the synthetic efficacy to synthesize polyheterocycles with highly valuable complex structures and stereogenic complexity from easily available starting materials. The further challenging application of this method is presently under antibacterial bioactive investigation in our laboratory.

## Figures and Tables

**Table 1:** Optimization of reaction conditions *^a^*.

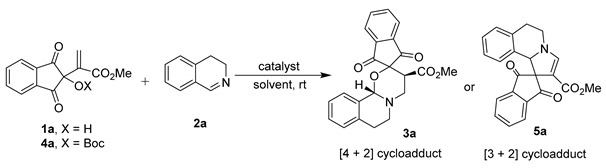					
Entry	Substrate	Catalyst	Solvent	Time (h)	Yield (%) *^b^*
1	**1a**	DABCO	CH_2_Cl_2_	12	**3a**, 78
2	**1a**	TEA	CH_2_Cl_2_	12	**3a**, 73
3	**1a**	DIPEA	CH_2_Cl_2_	12	**3a**, 69
4	**1a**	DBU	CH_2_Cl_2_	12	**3a**, trace
5	**1a**	Na_2_CO_3_	CH_2_Cl_2_	12	**3a**, trace
6	**1a**	NaOH	CH_2_Cl_2_	12	**3a**, trace
7	**1a**	DABCO	CHCl_3_	24	**3a**, 72
8	**1a**	DABCO	toluene	24	**3a**, 36
9	**1a**	DABCO	CH_3_CN	12	**3a**, 85
10	**1a**	DABCO	dioxane	24	**3a**, trace
11	**1a**	DABCO	THF	24	**3a**, trace
12	**1a**	DABCO	EtOAc	12	**3a**, 81
13 *^c^*	**1a**	DABCO	CH_3_CN	12	**3a**, 86
14	**4a**	DABCO	CH_3_CN	6	**5a**, 75
15	**4a**	/	CH_3_CN	6	**5a**, 78
16	**4a**	/	CH_2_Cl_2_	1	**5a**, 88
17	**4a**	/	CHCl_3_	1	**5a**, 85
18	**4a**	/	EtOAc	12	**5a**, 80
19	**4a**	/	Toluene	12	**5a**, 69
20	**4a**	/	THF	6	**5a**, 58

*^a^* Unless noted otherwise, reactions were performed with ninhydrin-derived MBH adduct **1a** or **4a** (0.11 mmol, 1.1 equiv) and 3,4-dihydroisoquinoline **2a** (0.1 mmol, 1.0 equiv), catalyst (20 mol%) in solvent (1.0 mL). *^b^* Yield of the isolated product and *dr* > 25:1 by ^1^H NMR analysis. *^c^* DABCO (50 mol%).

## Data Availability

Data are contained within the article or Supplementary Material.

## Supplementary Materials

The following supporting information can be downloaded at: https://www.mdpi.com/article/10.3390/molecules28196761/s1, Figure S1: Crystal data and structural refinement for **3n** and **5b**, Figure S2: NMR spectra.

## Sample Availability

Samples of the compounds are available from the authors.
