# Supplementary material for: Substrate-Controlled Diversity-Oriented Synthesis of Novel Polycyclic Frameworks via [4 + 2] and [3 + 2] Annulations of Ninhydrin-Derived MBH Adducts with 3,4-Dihydroisoquinolines
Source: Molecules. 2023 Sep 22;28(19):6761. doi: 10.3390/molecules28196761 (PMC10574269; doi:10.3390/molecules28196761)
Supplement: Supplementary file 1 [file molecules-28-06761-s001.zip › molecules-2596441-supplementary.pdf]

Substrate-Controlled Diversity-Oriented Synthesis of Novel Polycyclic Frameworks via [4 + 2] and [3 + 2] Annulations of Ninhydrin-Derived MBH Adducts with 3,4-Dihydroisoquinolines

Kaikai Wang [1], Wenwen Zhou [1], Jun Jia [2], Junwei Ye [1], Mengxin Yuan [1], Jie Yang [3,*], Yonghua Qi [1] and Rongxiang Chen [1,*]

1 School of Pharmacy, Xinxiang University, Xinxiang 453000, China; wangkaikai@xxu.edu.cn (K.W.); h200011250107@163.com (W.Z.); y2559356242@126.com (J.Y.); 13333806426@163.com (M.Y.); qyh@xxu.edu.cn (Y.Q.)
2 Jilin Province Product Quality Supervision and Inspection Institute, Changchun 130012, China; beyond-868@163.com
3 School of Chemistry & Materials Engineering, Xinxiang University, Xinxiang 453000, China
* Correspondence: yangjie19881127@126.com (J.Y.); chenrx@xxu.edu.cn (R.C.)

Supporting Information

Figure of Contents

Figure S1. Crystal data and structural refinement for 3n and 5b

Figure S2. NMR spectra

Figure S1. Crystal data and structural refinement for 3n and 5b

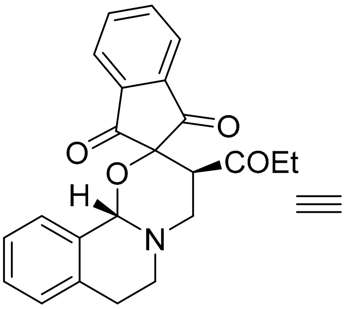

3n

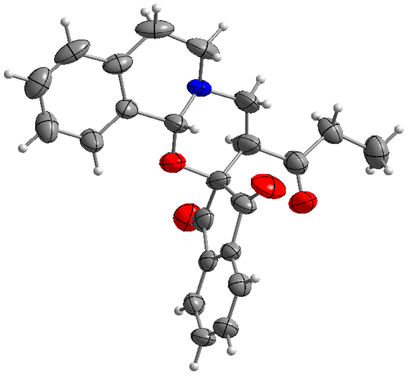

CCDC 2277407

Identification code	**3n**
Empirical formula	$C_{23}H_{21}NO_4$
Formula weight	375.41
Temperature/K	296
Crystal system	triclinic
Space group	P -1
a/Å	9.6843(12)
b/Å	9.8431(13)
c/Å	11.0404(14)
α/°	67.832(2)
β/°	83.814(2)
γ/°	78.540(2)
Volume/Å^3	954.6(2)
Z	2
ρ_{calc}g/cm^3	1.306
μ/mm^{-1}	0.090
F(000)	396
Crystal size/mm^3	0.25 × 0.23 × 0.22
Radiation	MoKα (λ = 0.71073)
2Θ range for data collection/°	2.84 to 27.59

Index ranges	$-11 \le h \le 11$, $-11 \le k \le 8$, $-13 \le l \le 9$
Reflections collected	3338
Independent reflections	2242 [R_{int} = 0.0133]
Data/restraints/parameters	3338 / 0 / 254
Goodness-of-fit on F^2	1.036
Final R indexes [I>=2σ (I)]	R_1 = 0.1072, wR_2 = 0.2953
R indices (all data)	R_1 = 0.1421, wR_2 = 0.3162
Largest diff. peak and hole /1-sigma level	1.468 / −0.515 / 0.071

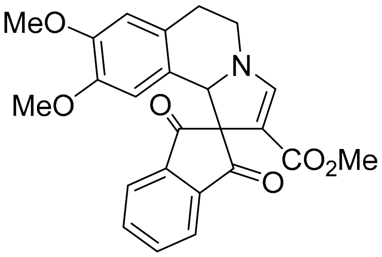

5b

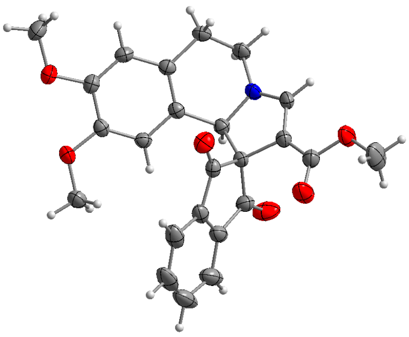

CCDC 2277406

Identification code	**5b**
Empirical formula	$C_{24}H_{21}NO_6$
Formula weight	419.42
Temperature/K	296
Crystal system	triclinic
Space group	P -1
a/Å	8.7361(13)
b/Å	9.9322(15)
c/Å	15.173(2)
α/°	106.255(2)
β/°	90.612(2)
γ/°	110.421(2)
Volume/Å^3	1175.8(3)

Z	2
ρ_{calc}g/cm^3	1.424
μ/mm^{-1}	0.318
F(000)	524
Crystal size/mm^3	0.26 × 0.25 × 0.20
Radiation	MoKα (λ = 0.71073)
2Θ range for data collection/°	2.30 to 27.37
Index ranges	− 8 ≤ h ≤ 10, − 11 ≤ k ≤ 9, − 17 ≤ l ≤ 18
Reflections collected	4105
Independent reflections	3232 [R_{int} = 0.0145]
Data/restraints/parameters	4105 / 0 / 310
Goodness-of-fit on F^2	1.012
Final R indexes [I>=2σ (I)]	R_1 = 0.0470, wR_2 = 0.1373
R indices (all data)	R_1 = 0.0601, wR_2 = 0.1457
Largest diff. peak and hole/ 1-sigma level	0.476 / − 0.714 / 0.052

Figure S2. NMR spectra

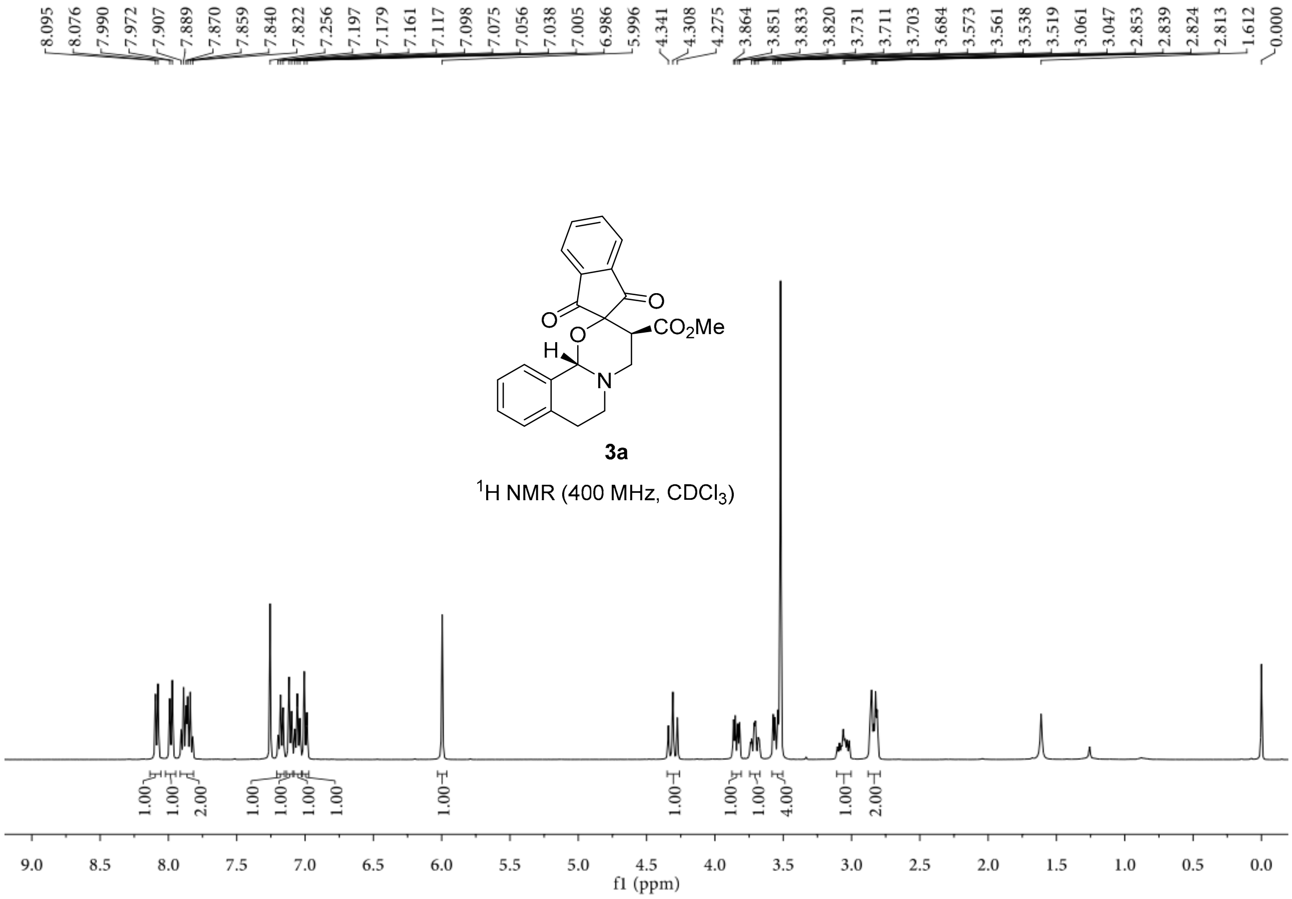

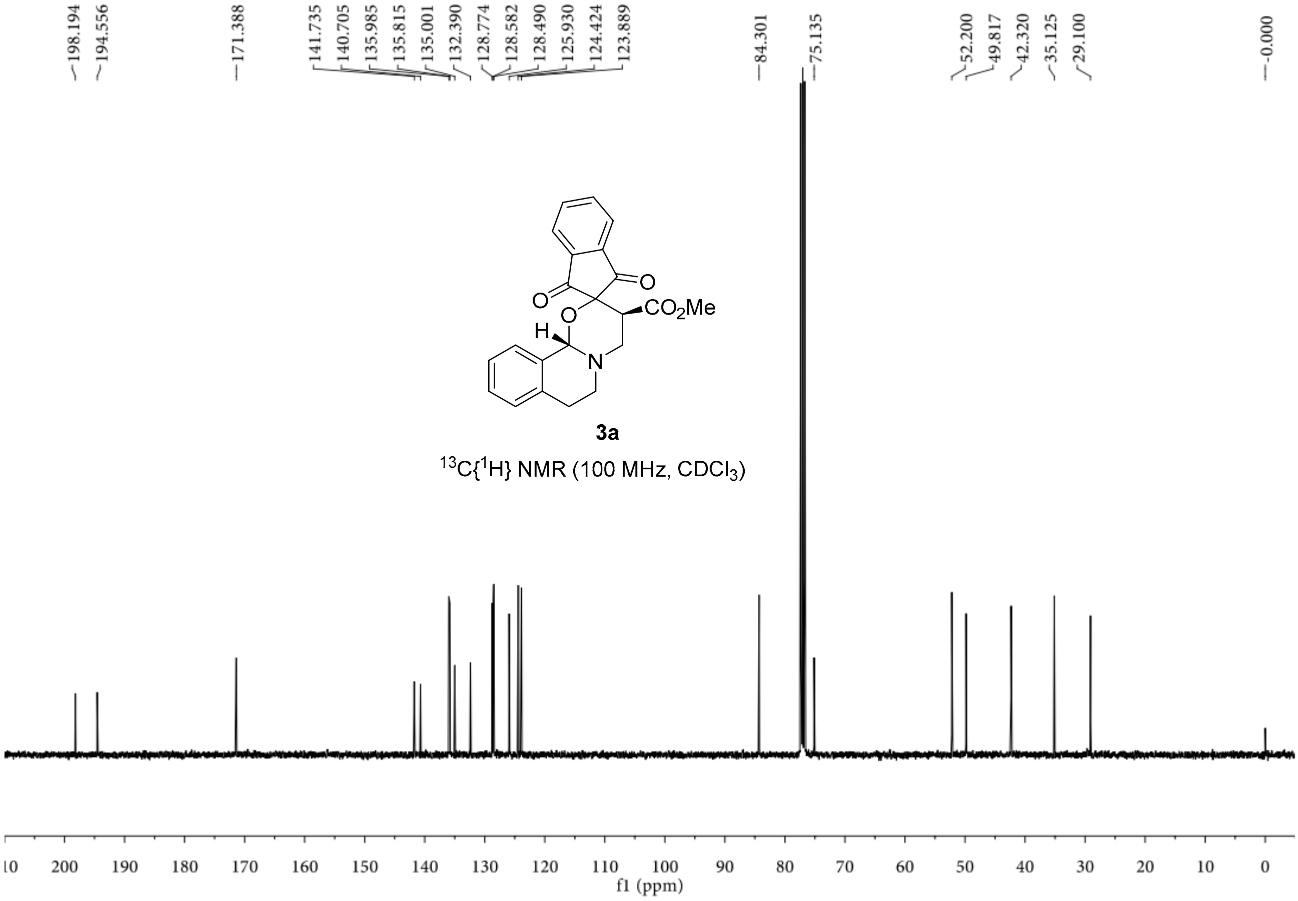

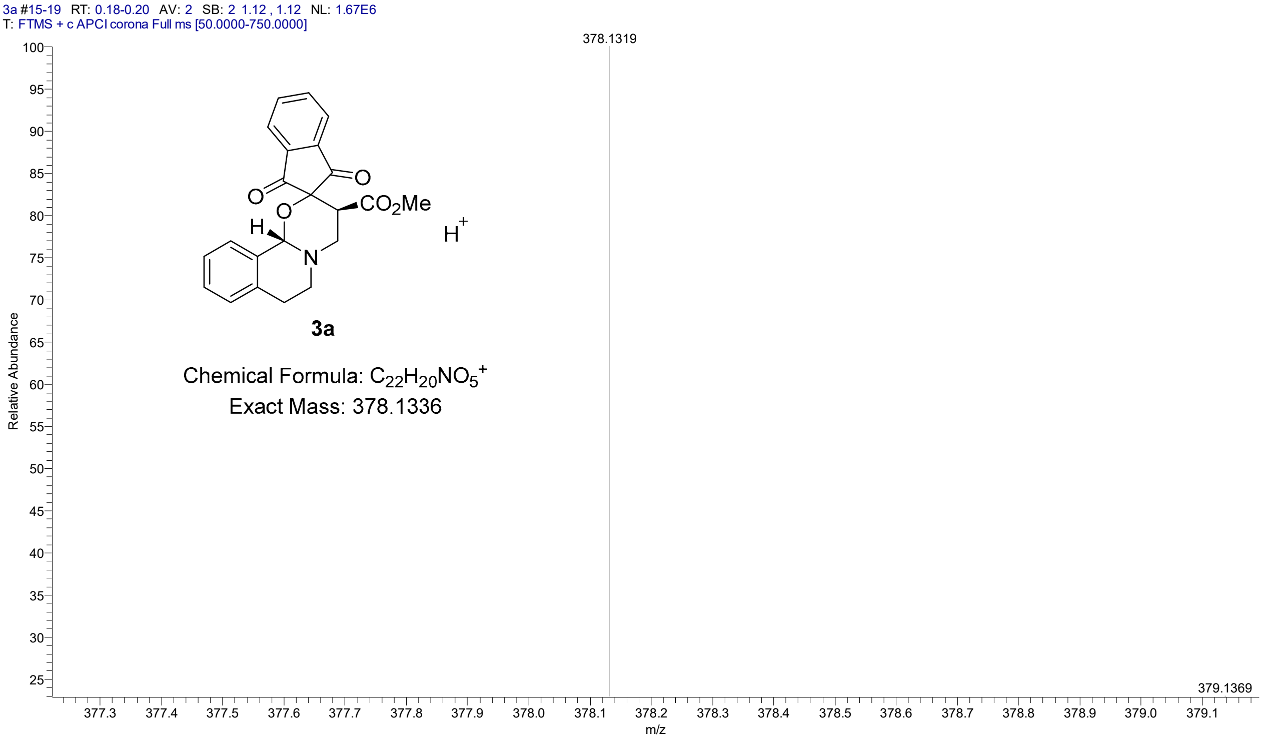

[M + H]+ calcd for $C_{22}H_{20}NO_5$ 378.1336, found 378.1319.

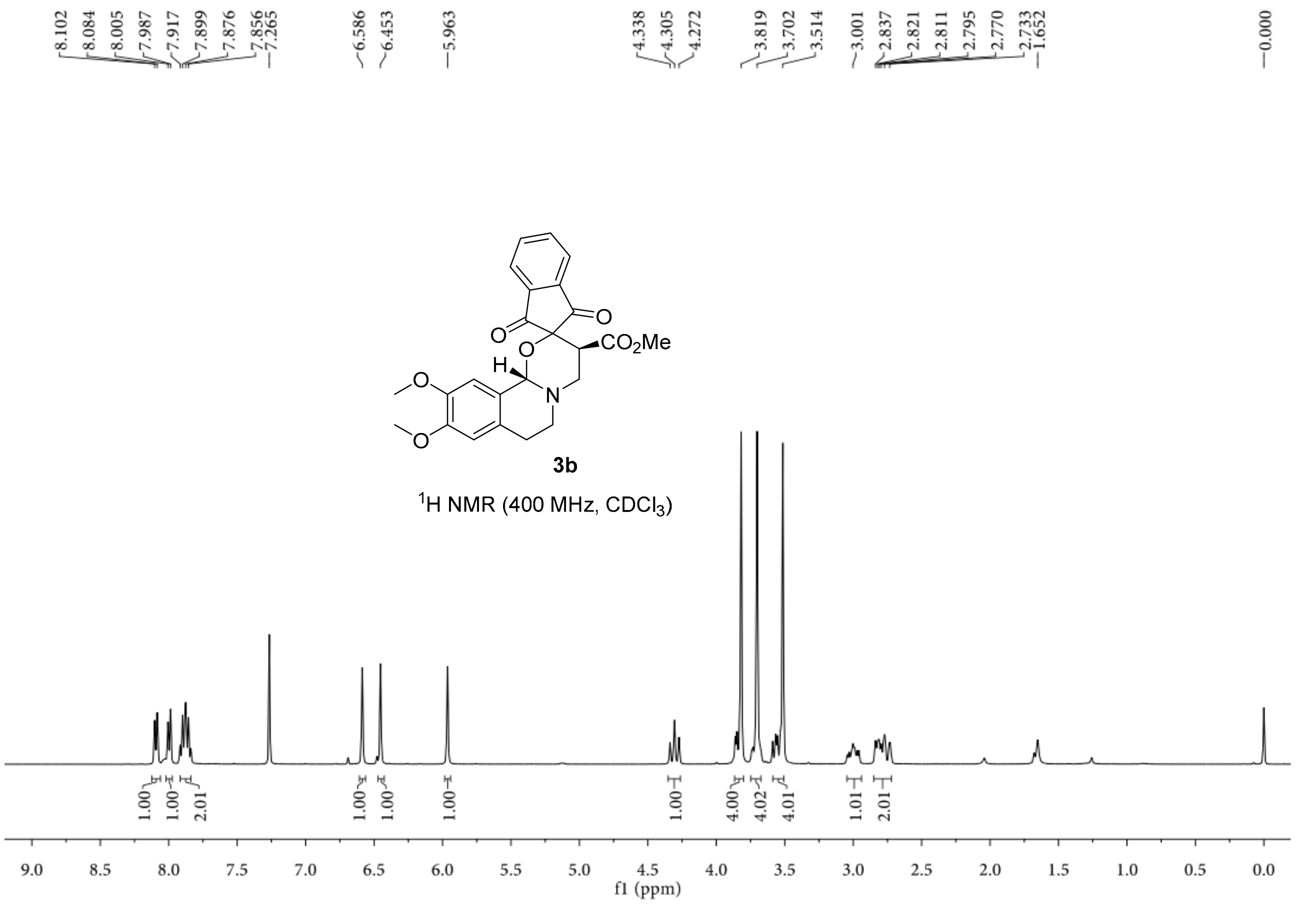
8.102 8.084 8.005 7.987 7.917 7.899 7.876 7.856 7.265 6.586 6.453 5.963 4.338 4.305 4.272 3.819 3.702 3.514 3.001 2.837 2.821 2.811 2.795 2.770 2.733 1.652 0.000
CO$_2$Me
3b
^{1}H NMR (400 MHz, CDCl$_3$)
1.00 1.00 2.01 1.00 1.00 1.00 1.00 4.00 4.02 4.01 1.01 2.01
f1 (ppm)

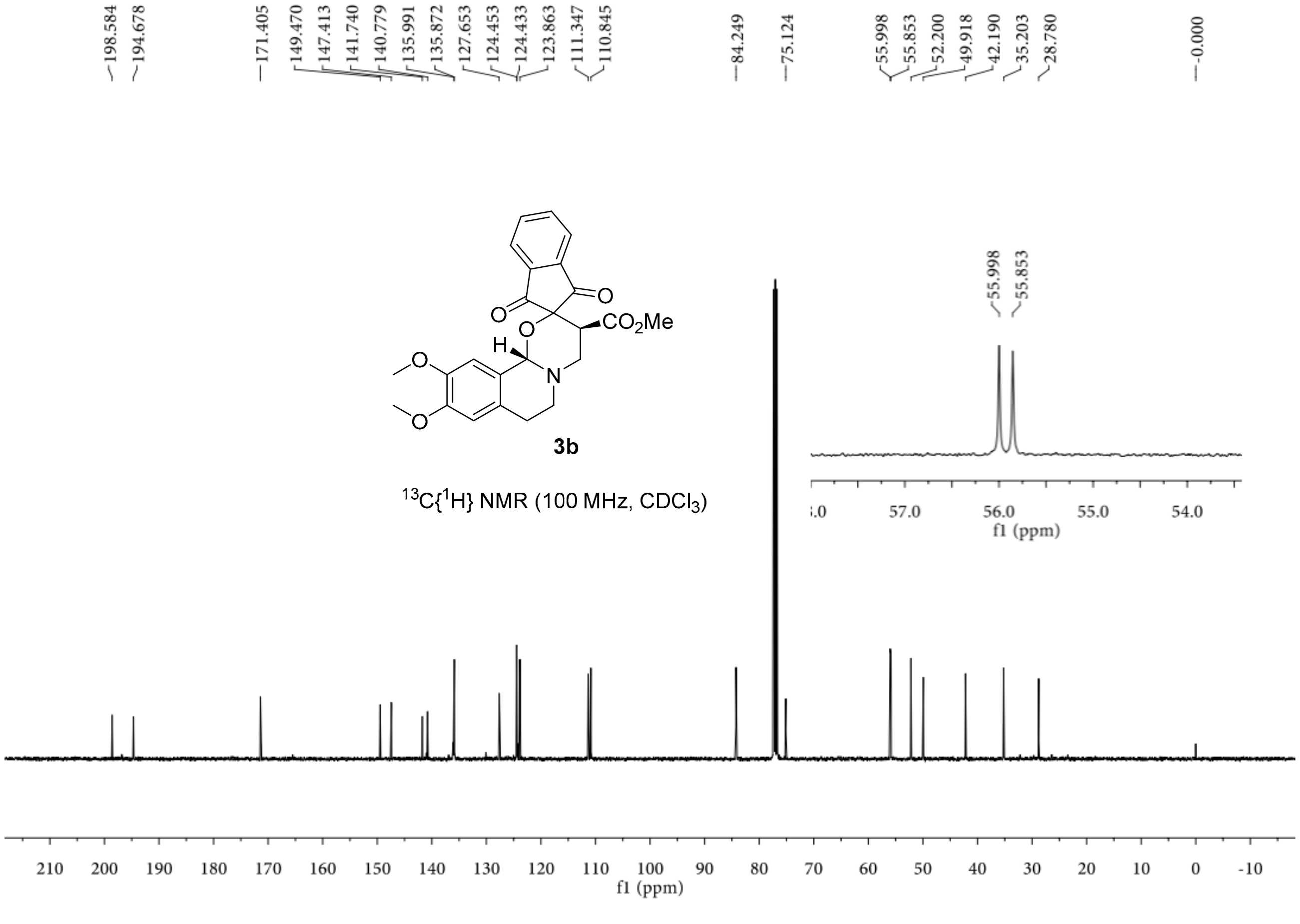
198.584 194.678 171.405 149.470 147.413 141.740 140.779 135.991 135.872 127.653 124.453 124.433 123.863 111.347 110.845 84.249 75.124 55.998 55.853 52.200 49.918 42.190 35.203 28.780 -0.000
CO$_2$Me
3b
^{13}C{^{1}H} NMR (100 MHz, CDCl$_3$)
55.998 55.853
f1 (ppm)

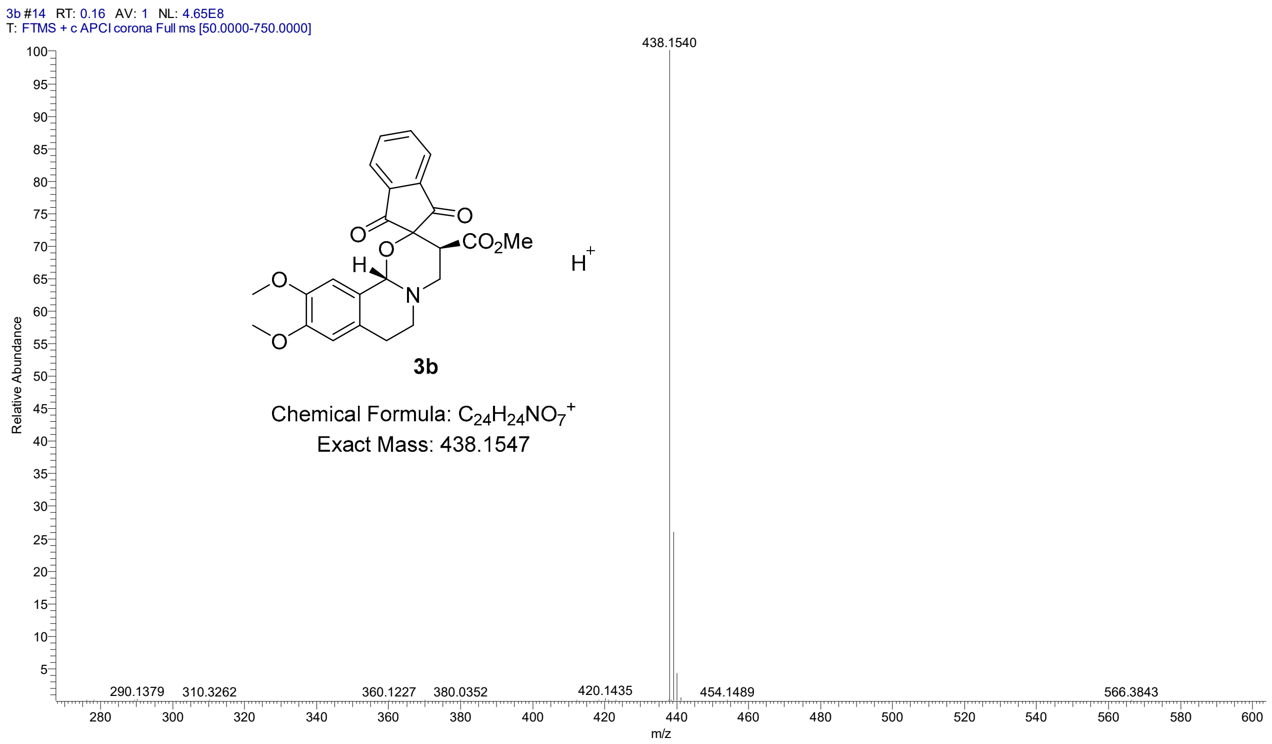

[M + H]+ calcd for $C_{24}H_{24}NO_7$ 438.1547, found 438.1540.

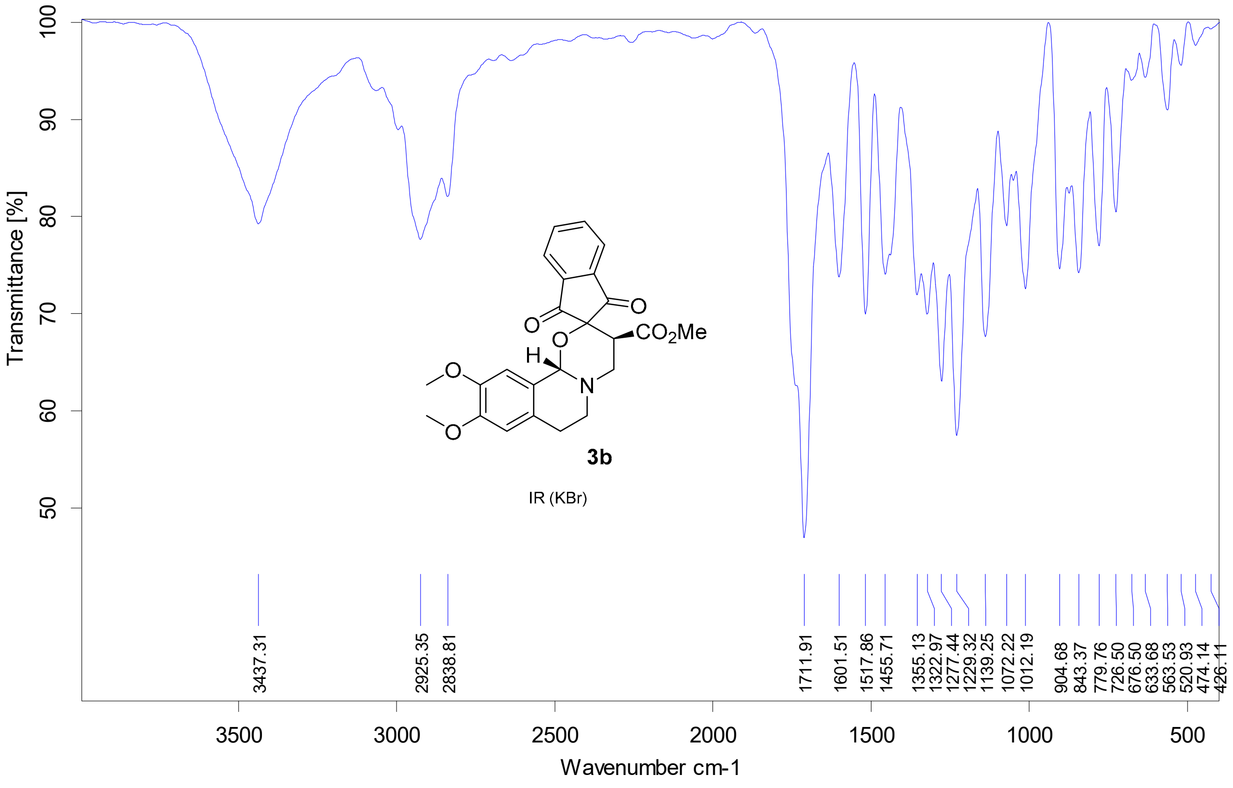

E:\2023\0913\1.0	bioi5-1-1-1	Instrument type and / or accessory	31/12/2008

Page 1/1

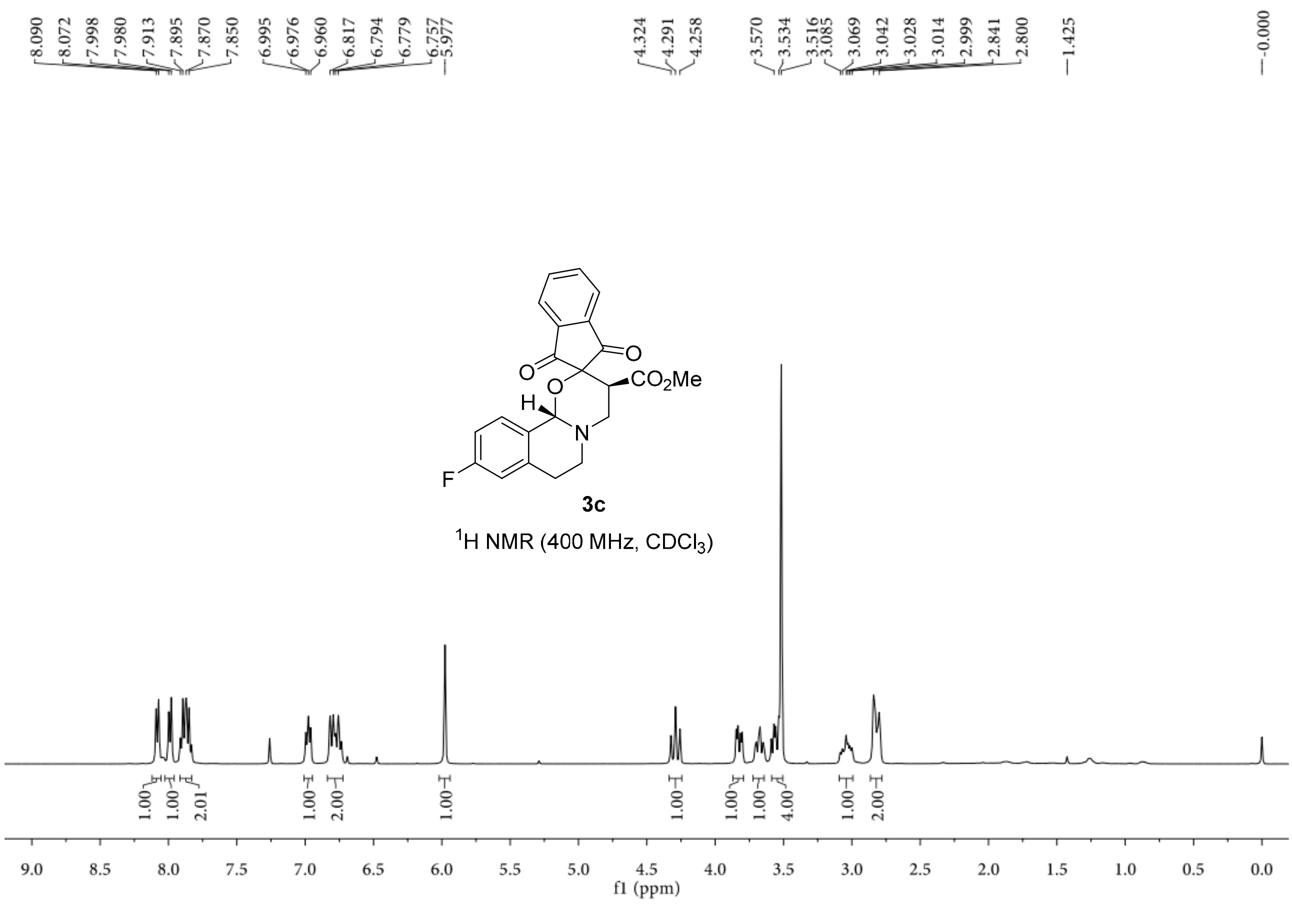

3c
^{1}H NMR (400 MHz, $CDCl_3$)
f1 (ppm)

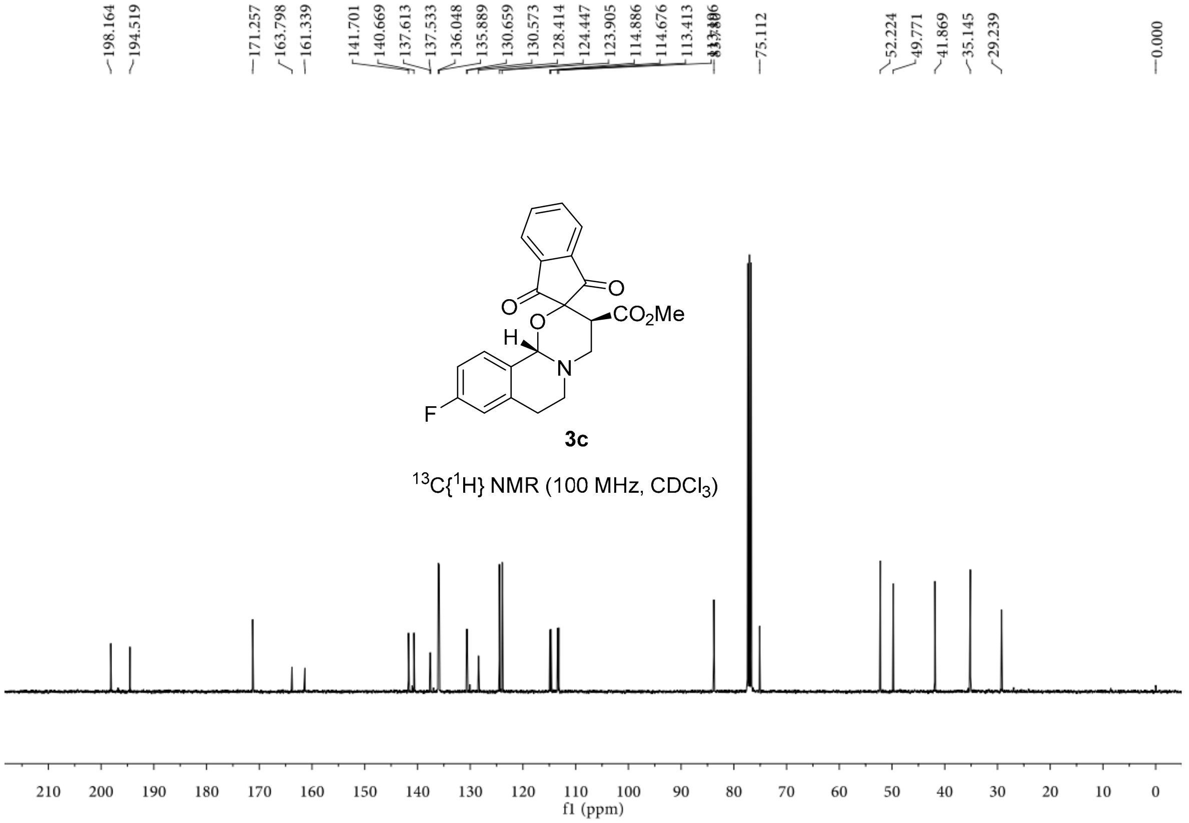

3c
$^{13}C\{^{1}H\}$ NMR (100 MHz, $CDCl_3$)
f1 (ppm)

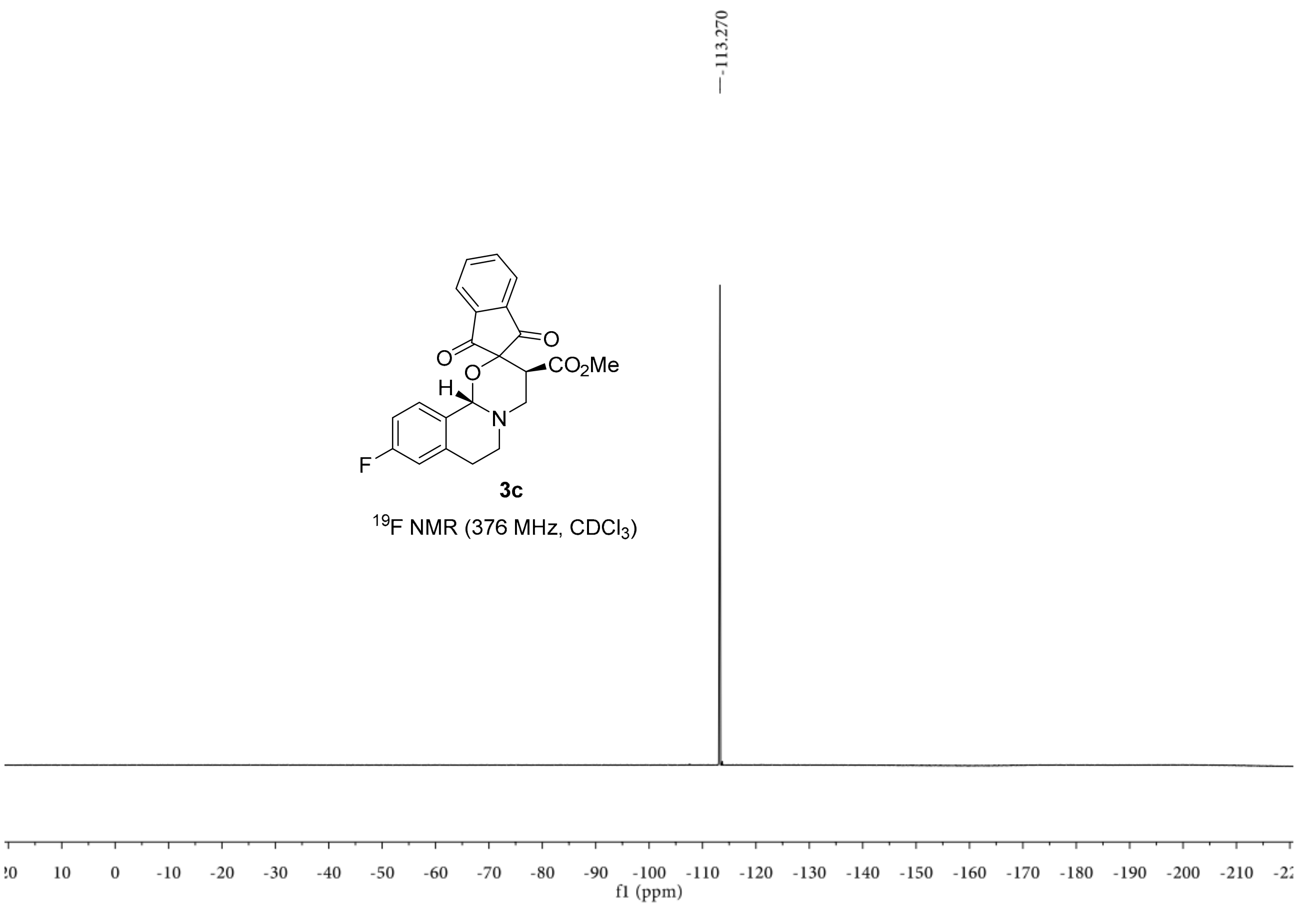

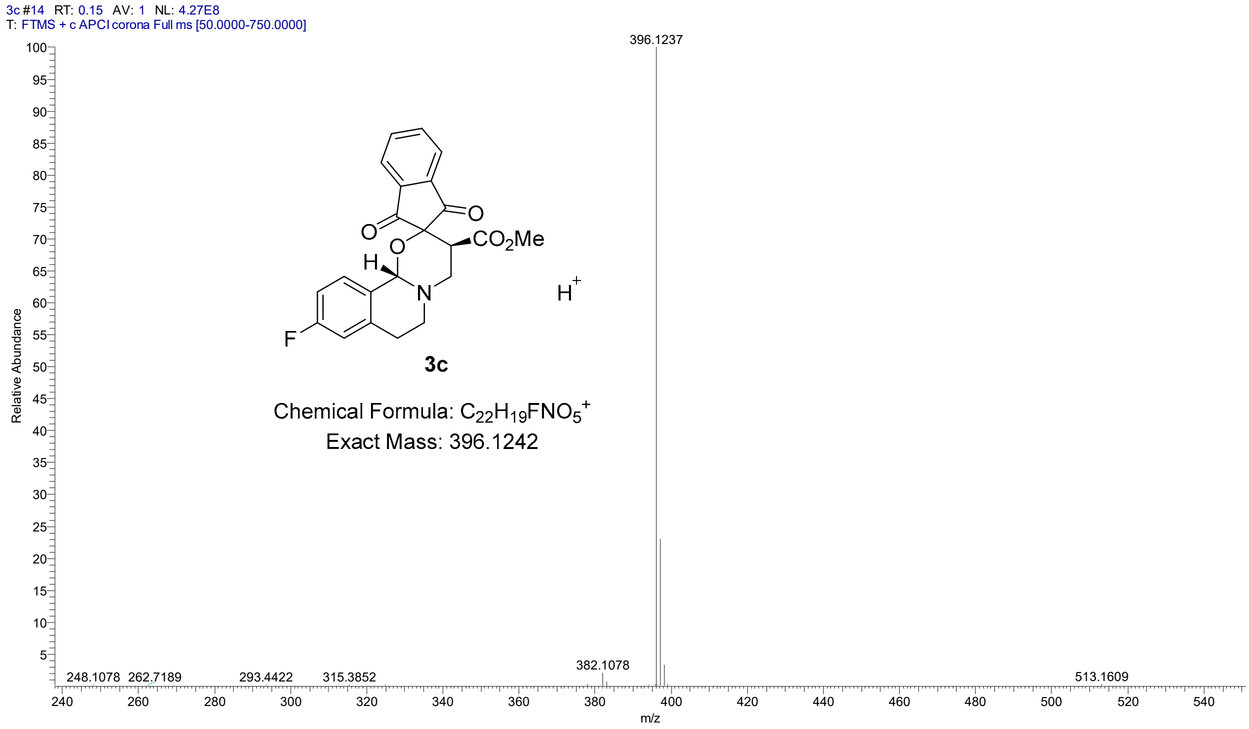

$[M + H]^+$ calcd for $C_{22}H_{19}FNO_5$ 396.1242, found 396.1237.

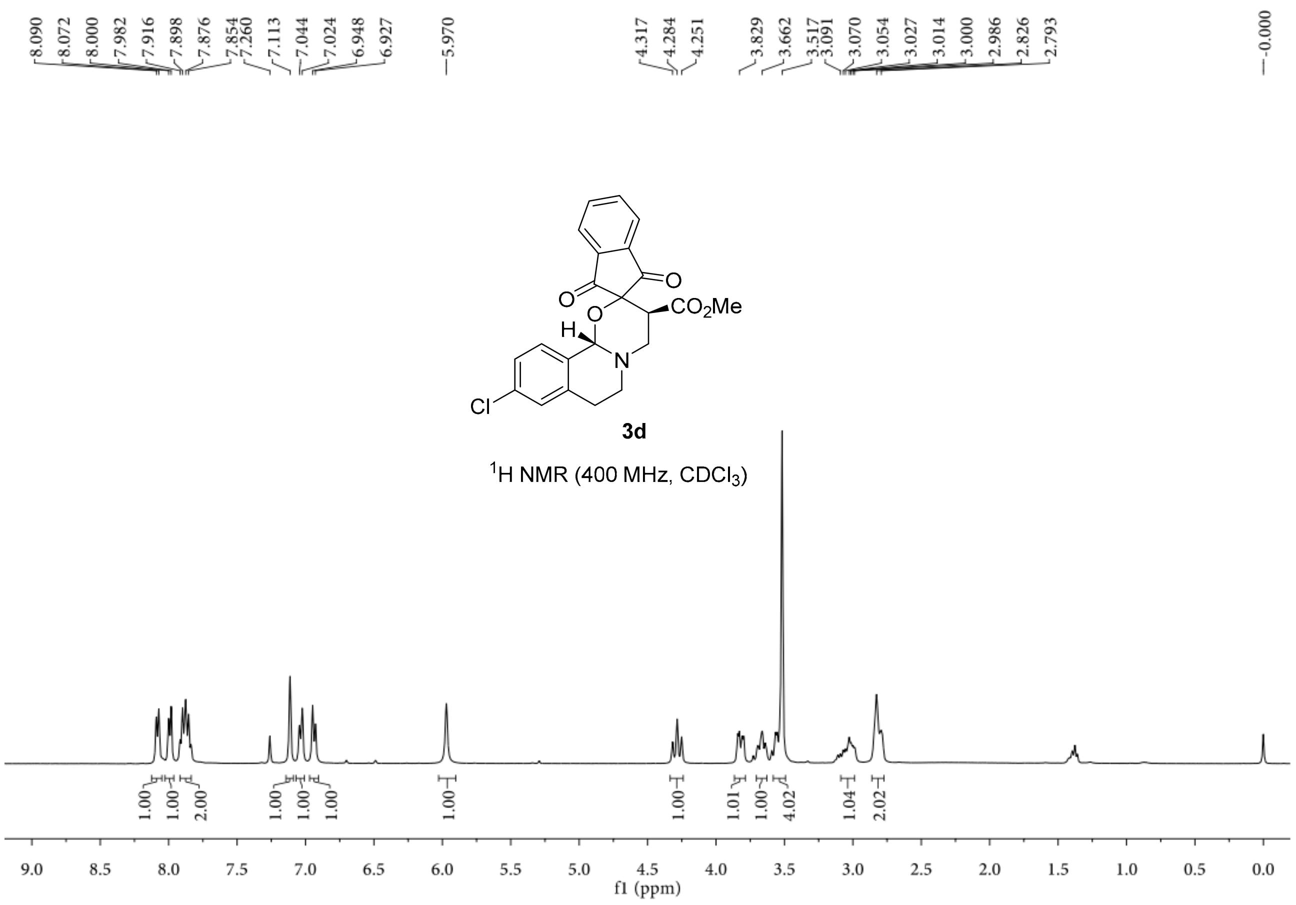
8.090 8.072 8.000 7.982 7.916 7.898 7.876 7.854 7.260 7.113 7.044 7.024 6.948 6.927
5.970
4.317 4.284 4.251
3.829 3.662 3.517 3.091 3.070 3.054 3.027 3.014 3.000 2.986 2.826 2.793
-0.000
O O CO$_2$Me H N O Cl
3d
^{1}H NMR (400 MHz, CDCl$_3$)
1.00 1.00 2.00 1.00 1.00 1.00 1.00 1.00 1.01 1.00 4.02 1.04 2.02
9.0 8.5 8.0 7.5 7.0 6.5 6.0 5.5 5.0 4.5 4.0 3.5 3.0 2.5 2.0 1.5 1.0 0.5 0.0
f1 (ppm)

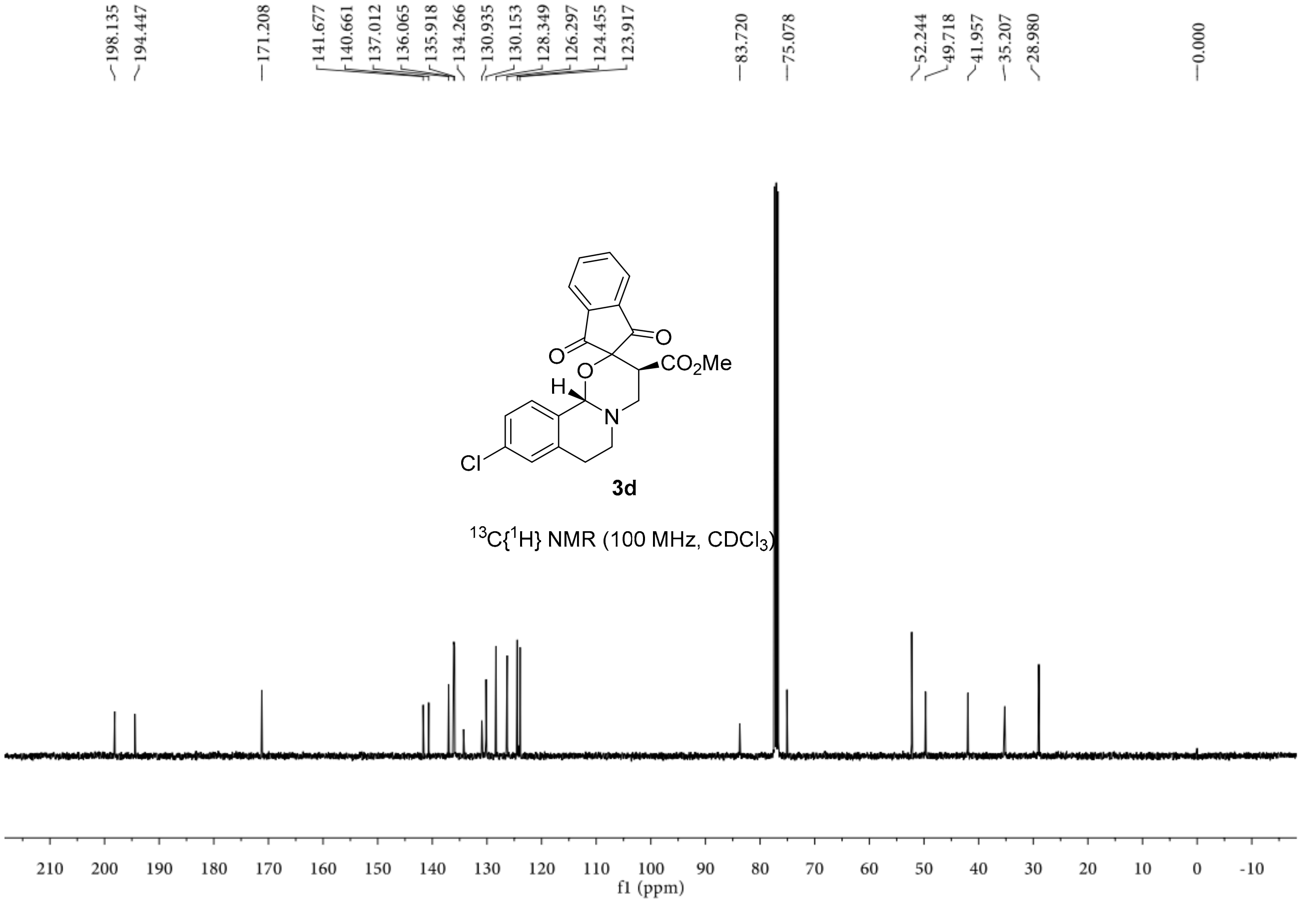
198.135 194.447
171.208
141.677 140.661 137.012 136.065 135.918 134.266 130.935 130.153 128.349 126.297 124.455 123.917
83.720
75.078
52.244 49.718 41.957 35.207 28.980
0.000
O O CO$_2$Me H N O Cl
3d
^{13}C{^{1}H} NMR (100 MHz, CDCl$_3$)
210 200 190 180 170 160 150 140 130 120 110 100 90 80 70 60 50 40 30 20 10 0 -10
f1 (ppm)

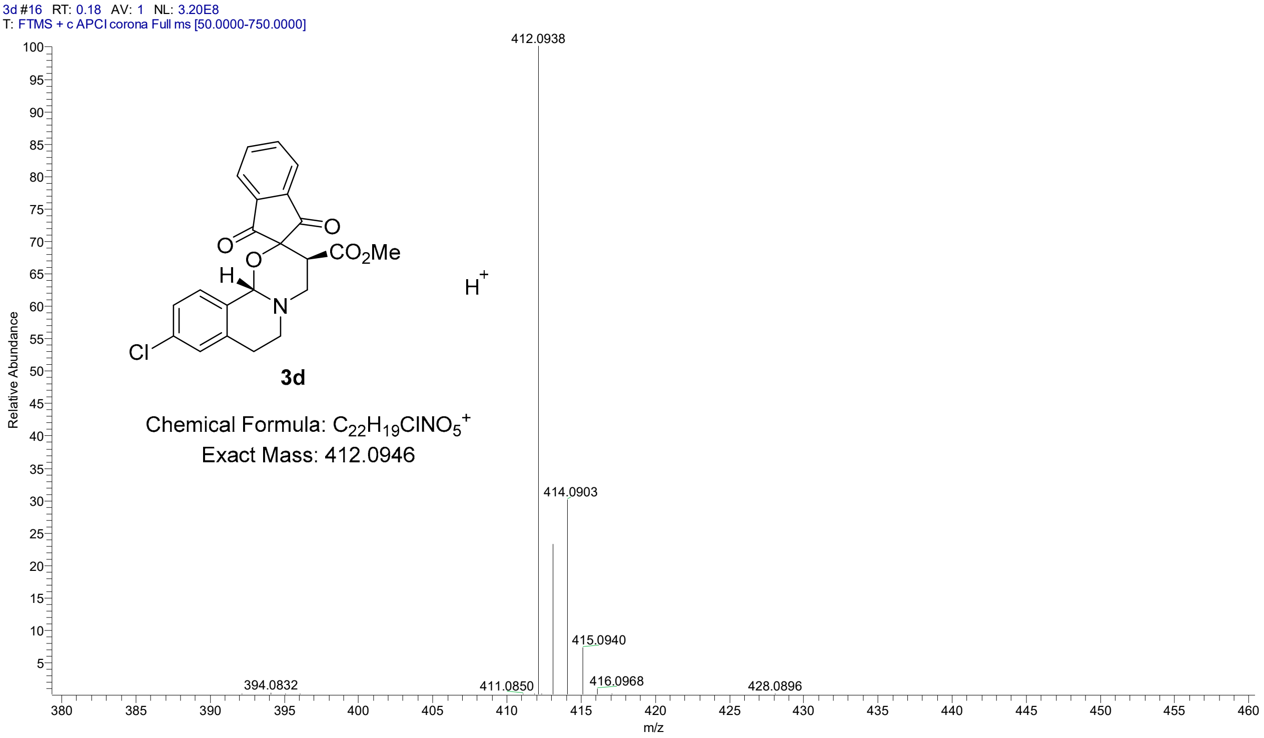

$[M + H]^+$ calcd for $C_{22}H_{19}ClNO_5$ 412.0946, found 412.0938.

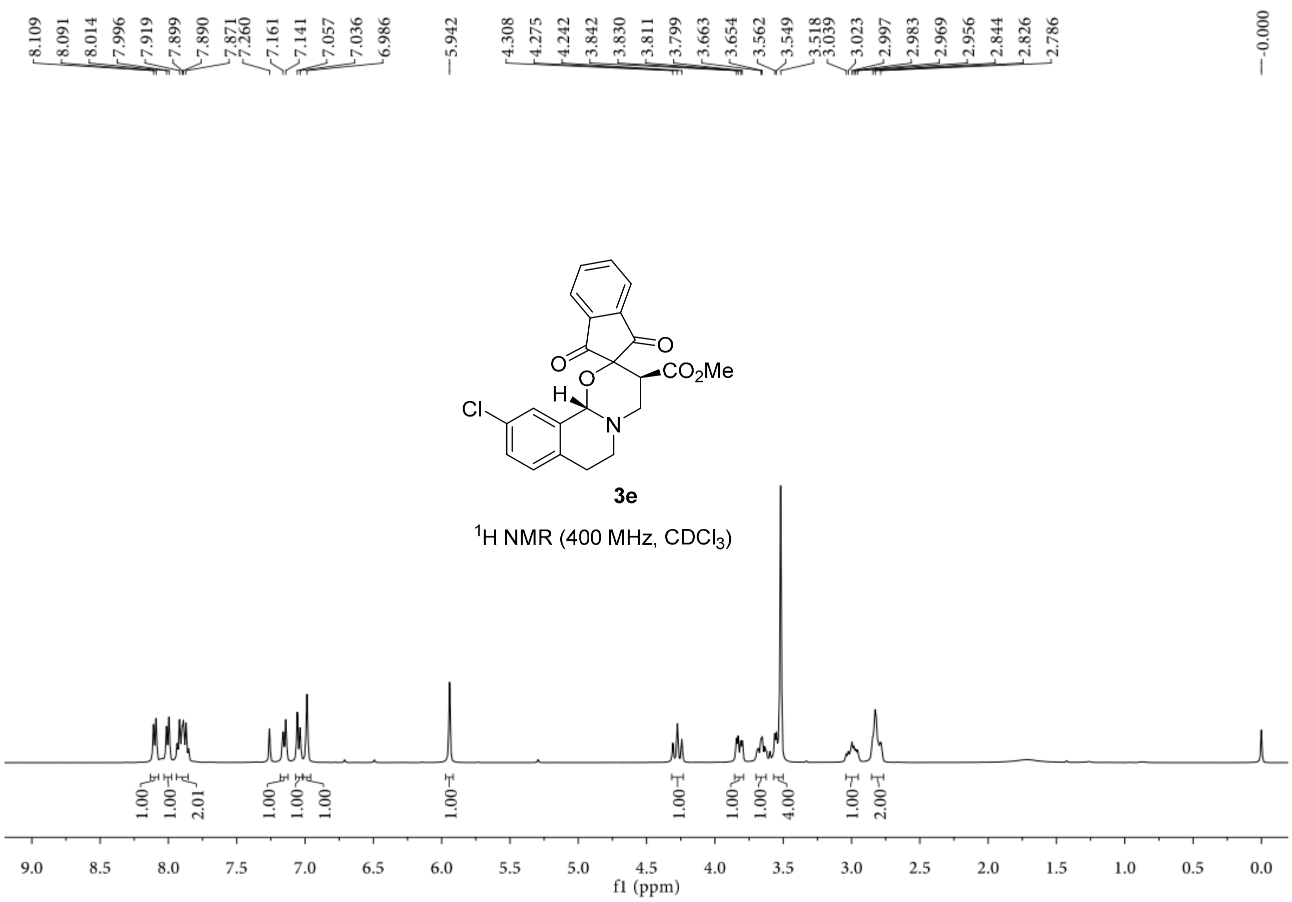
8.109 8.091 8.014 7.996 7.919 7.899 7.890 7.871 7.260 7.161 7.141 7.057 7.036 6.986
5.942
4.308 4.275 4.242 3.842 3.830 3.811 3.799 3.663 3.654 3.562 3.549 3.518 3.039 3.023 2.997 2.983 2.969 2.956 2.844 2.826 2.786
-0.000
O
O
CO2Me
H
O
Cl
N
3e
1H NMR (400 MHz, CDCl3)
1.00 1.00 2.01 1.00 1.00 1.00 1.00 1.00 1.00 1.00 4.00 1.00 2.00
9.0 8.5 8.0 7.5 7.0 6.5 6.0 5.5 5.0 4.5 4.0 3.5 3.0 2.5 2.0 1.5 1.0 0.5 0.0
f1 (ppm)

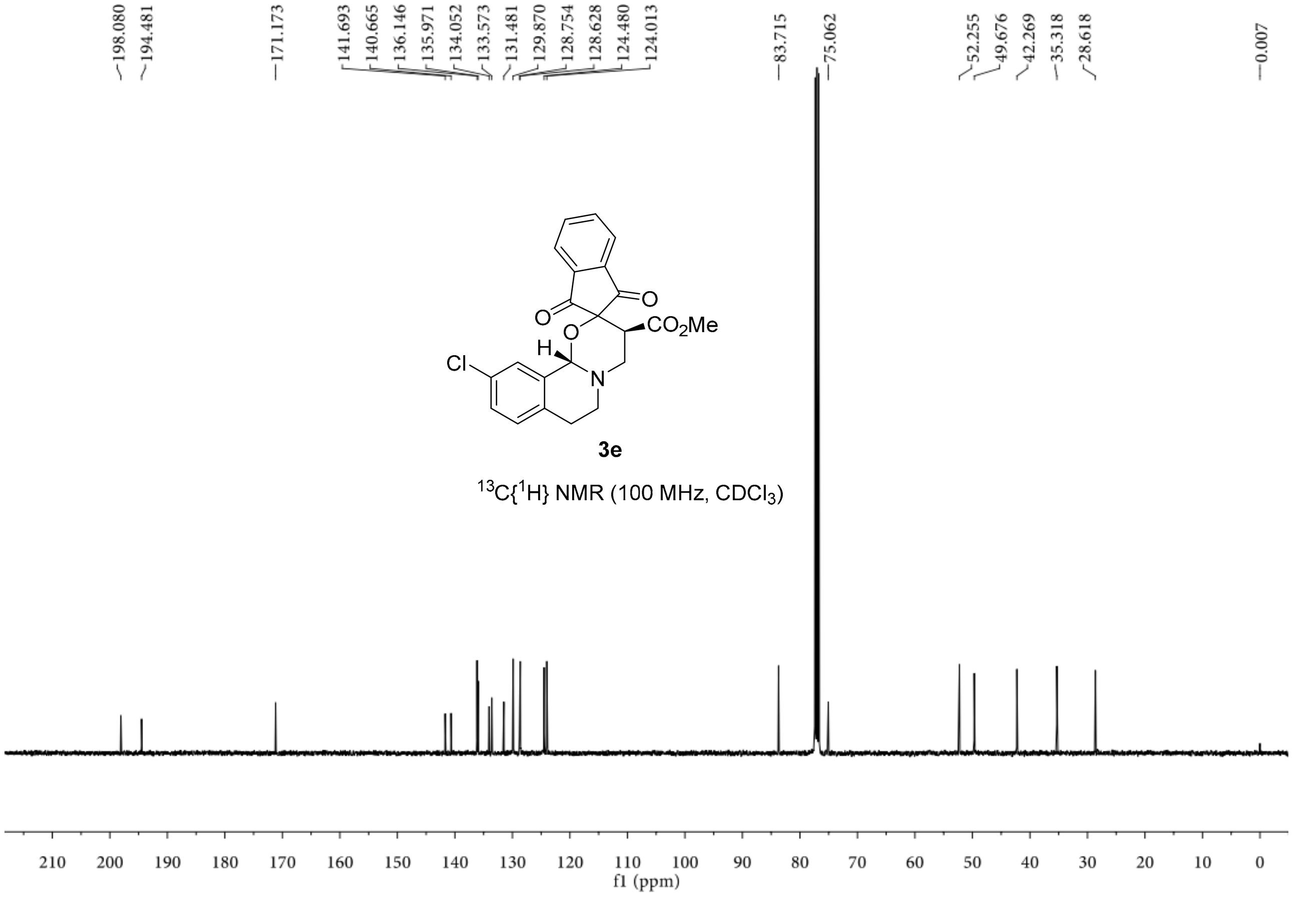
198.080 194.481
171.173
141.693 140.665 136.146 135.971 134.052 133.573 131.481 129.870 128.754 128.628 124.480 124.013
83.715 75.062
52.255 49.676 42.269 35.318 28.618
0.007
O
O
CO2Me
H
O
Cl
N
3e
13C{1H} NMR (100 MHz, CDCl3)
210 200 190 180 170 160 150 140 130 120 110 100 90 80 70 60 50 40 30 20 10 0
f1 (ppm)

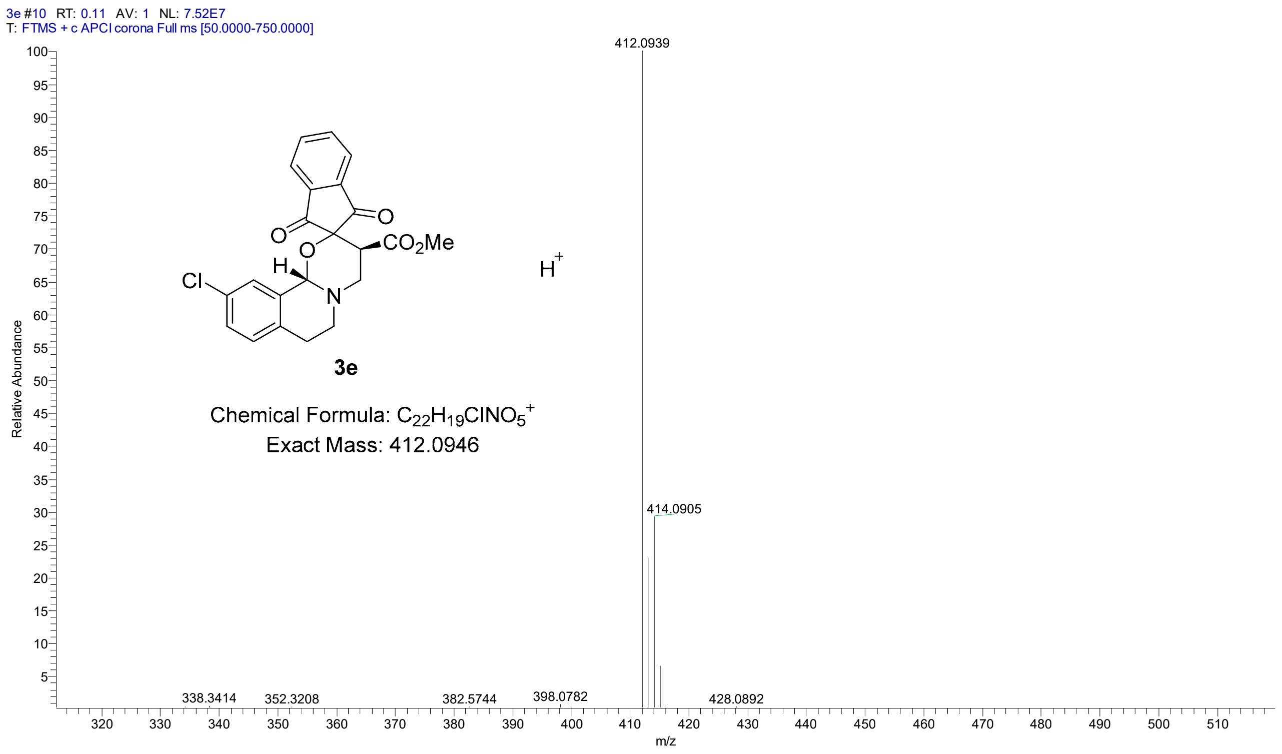

$[M + H]^+$ calcd for $C_{22}H_{19}ClNO_5$ 412.0946, found 412.0939.

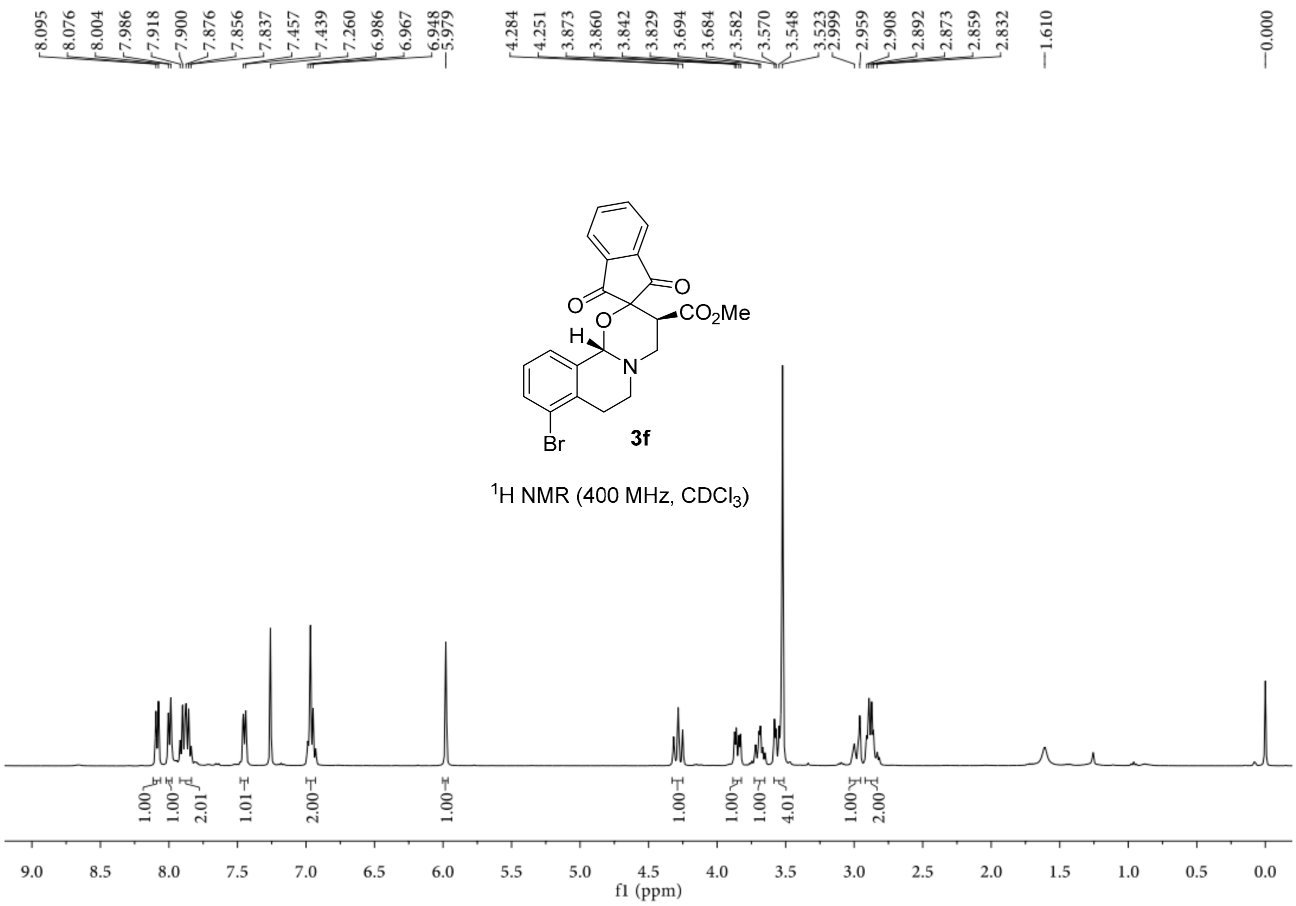
8.095
8.076
8.004
7.986
7.918
7.900
7.876
7.856
7.837
7.457
7.439
7.260
6.986
6.967
6.948
5.979
4.284
4.251
3.873
3.860
3.842
3.829
3.694
3.684
3.582
3.570
3.548
3.523
2.999
2.959
2.908
2.892
2.873
2.859
2.832
1.610
0.000
O
O
O
CO2Me
H
N
Br
3f
1H NMR (400 MHz, CDCl3)
1.00
1.00
2.01
1.01
2.00
1.00
1.00
1.00
1.00
4.01
1.00
2.00
f1 (ppm)

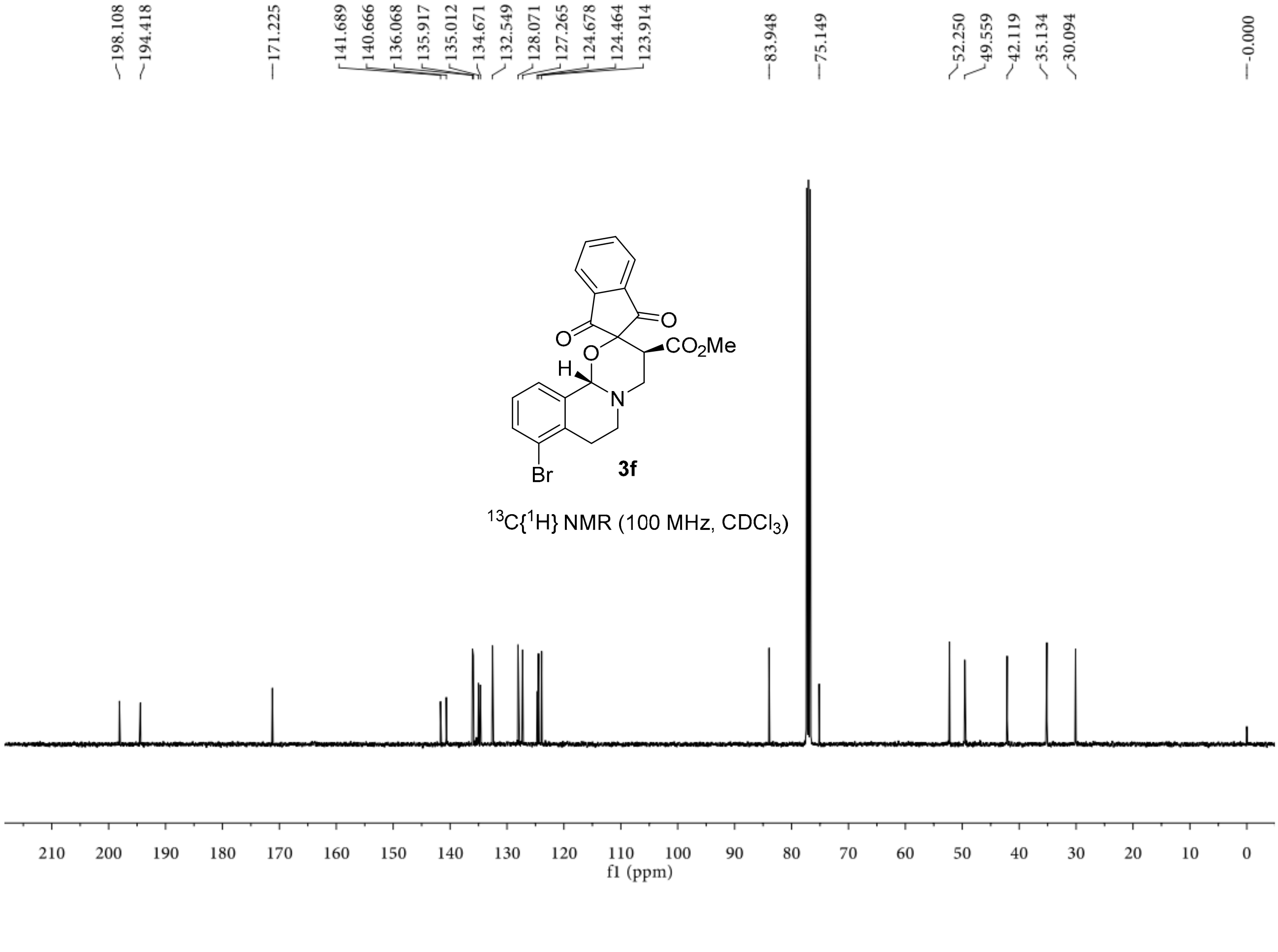
198.108
194.418
171.225
141.689
140.666
136.068
135.917
135.012
134.671
132.549
128.071
127.265
124.678
124.464
123.914
83.948
75.149
52.250
49.559
42.119
35.134
30.094
0.000
O
O
O
CO2Me
H
N
Br
3f
13C{1H} NMR (100 MHz, CDCl3)
f1 (ppm)

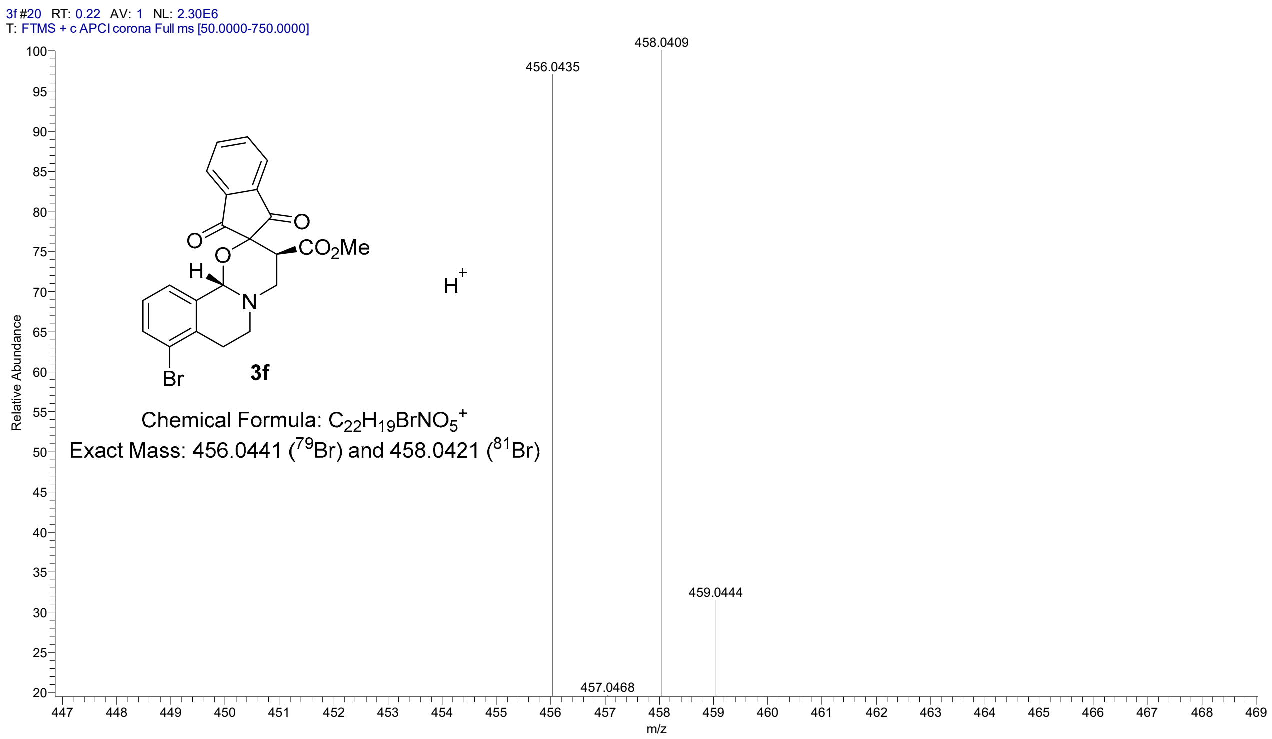

[M + H]$^+$ calcd for $C_{22}H_{19}BrNO_5$ 456.0441 (^{79}Br) and 458.0421 (^{81}Br), found 456.0435, 458.0409.

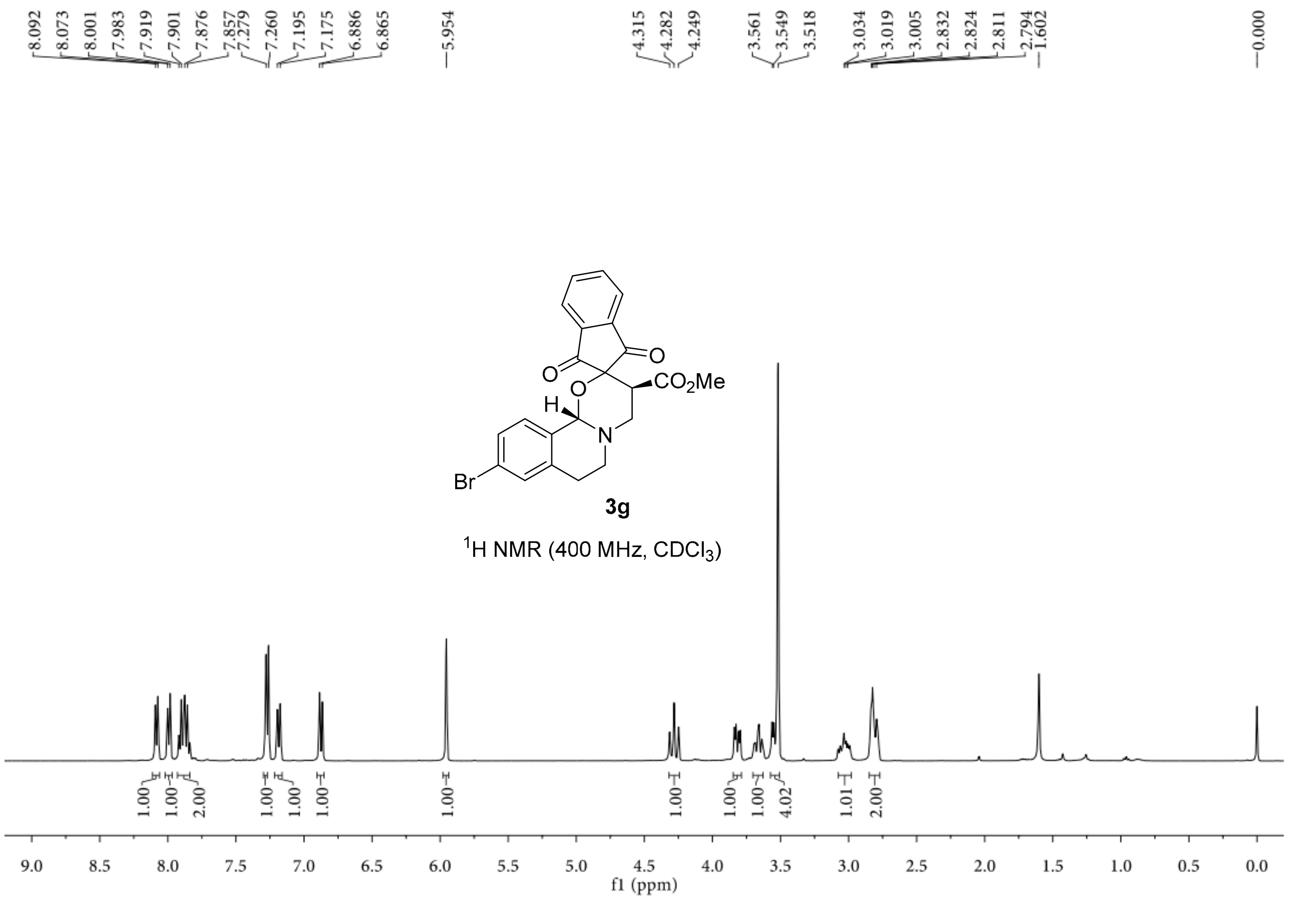
8.092 8.073 8.001 7.983 7.919 7.901 7.876 7.857 7.279 7.260 7.195 7.175 6.886 6.865 5.954 4.315 4.282 4.249 3.561 3.549 3.518 3.034 3.019 3.005 2.832 2.824 2.811 2.794 1.602 0.000
CO$_2$Me
O
H
N
Br
3g
^{1}H NMR (400 MHz, CDCl$_3$)
1.00 1.00 2.00 1.00 1.00 1.00 1.00 1.00 1.00 1.00 4.02 1.01 2.00
f1 (ppm)

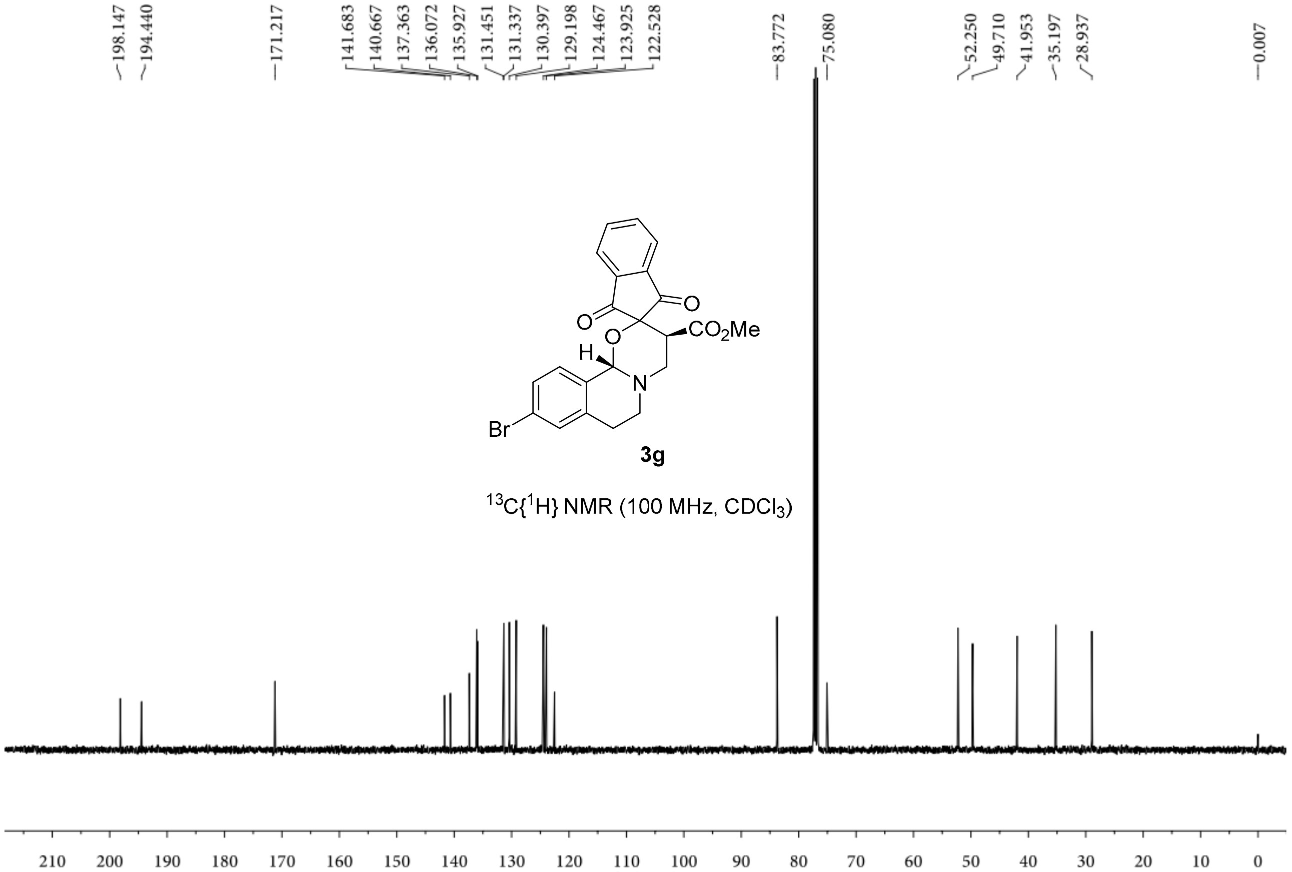
198.147 194.440 171.217 141.683 140.667 137.363 136.072 135.927 131.451 131.337 130.397 129.198 124.467 123.925 122.528 83.772 75.080 52.250 49.710 41.953 35.197 28.937 0.007
CO$_2$Me
O
H
N
Br
3g
^{13}C{^{1}H} NMR (100 MHz, CDCl$_3$)
f1 (ppm)

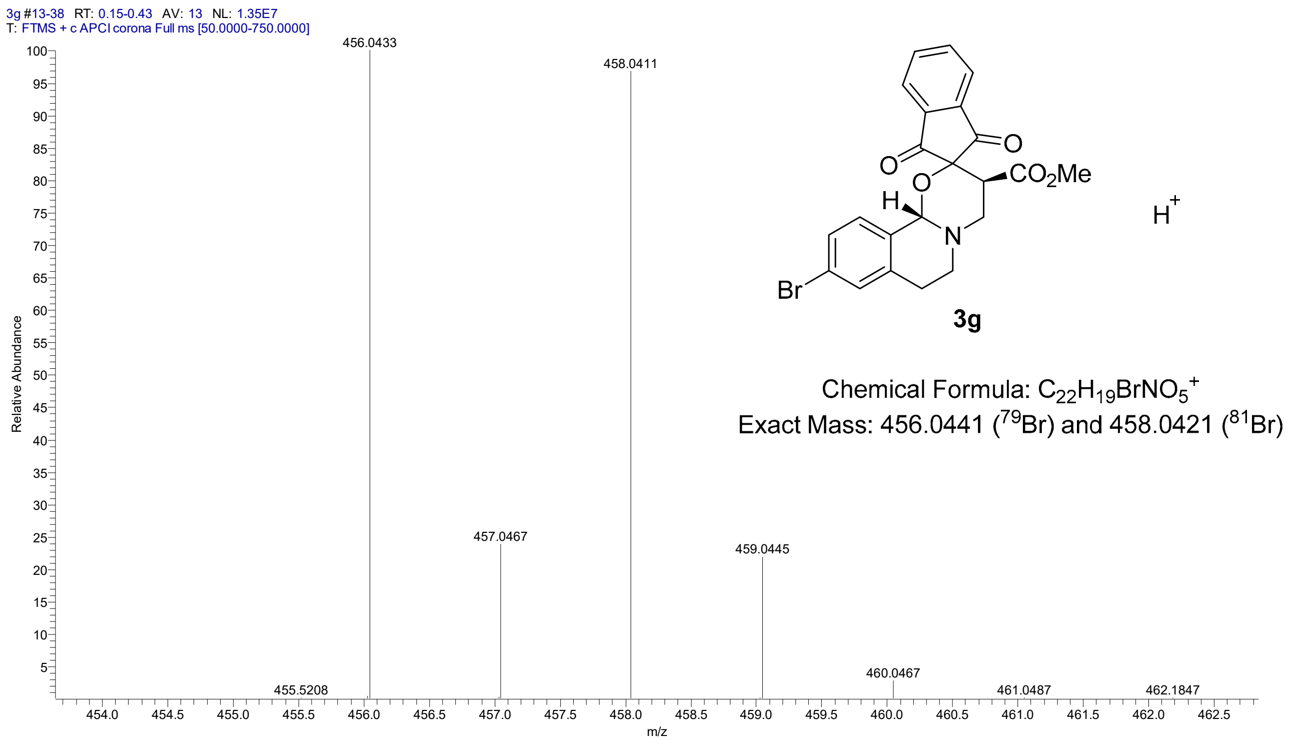

$[M + H]^+$ calcd for $C_{22}H_{19}BrNO_5$ 456.0441 (^{79}Br) and 458.0421 (^{81}Br), found 456.0433, 458.0411.

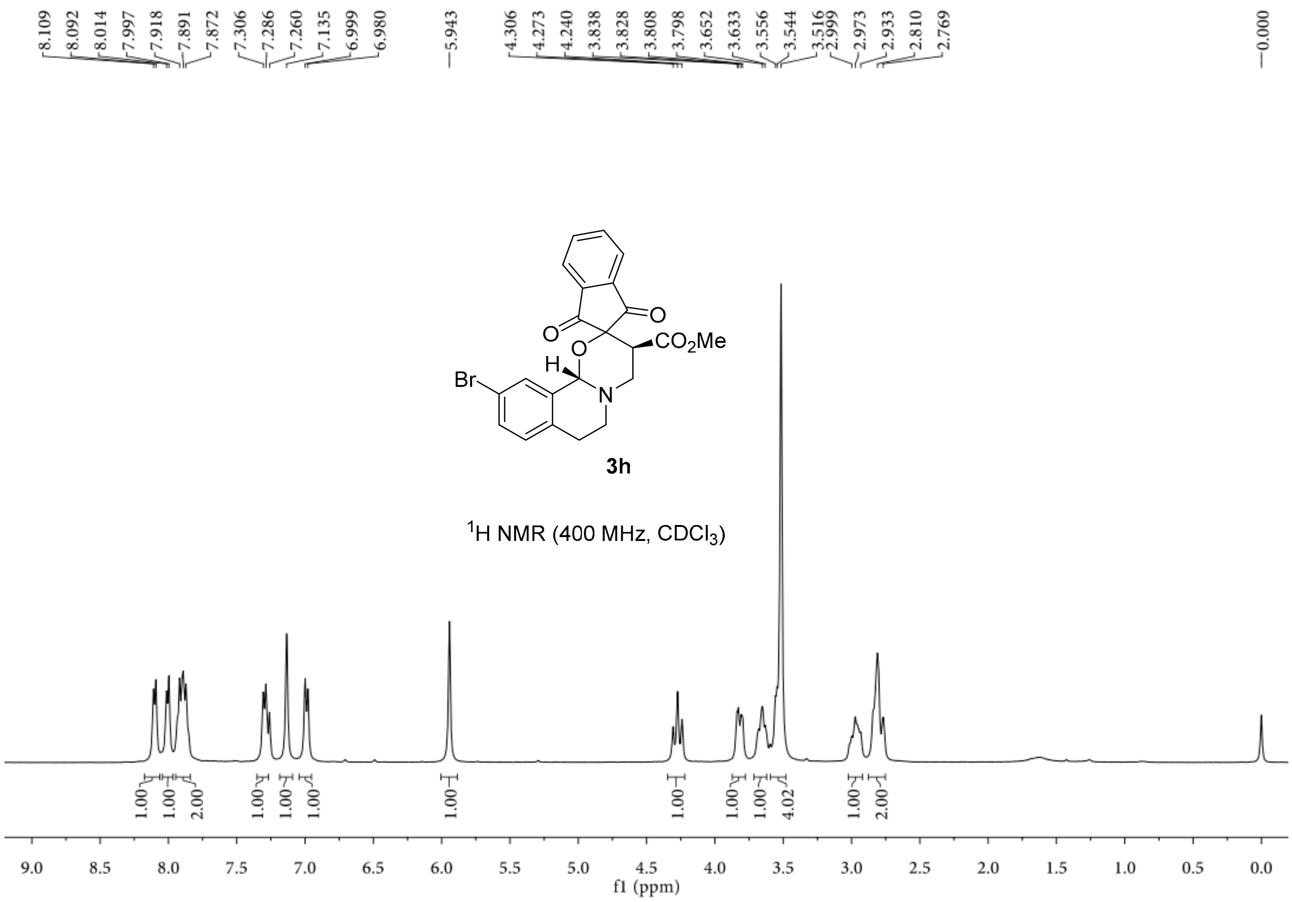
8.109
8.092
8.014
7.997
7.918
7.891
7.872
7.306
7.286
7.260
7.135
6.999
6.980
5.943
4.306
4.273
4.240
3.838
3.828
3.808
3.798
3.652
3.633
3.556
3.544
3.516
2.999
2.973
2.933
2.810
2.769
0.000
Br
H
O
N
CO_2Me
3h
^1H NMR (400 MHz, $CDCl_3$)
1.00
1.00
2.00
1.00
1.00
1.00
1.00
1.00
1.00
1.00
4.02
1.00
2.00
9.0
8.5
8.0
7.5
7.0
6.5
6.0
5.5
5.0
4.5
4.0
3.5
3.0
2.5
2.0
1.5
1.0
0.5
0.0
f1 (ppm)

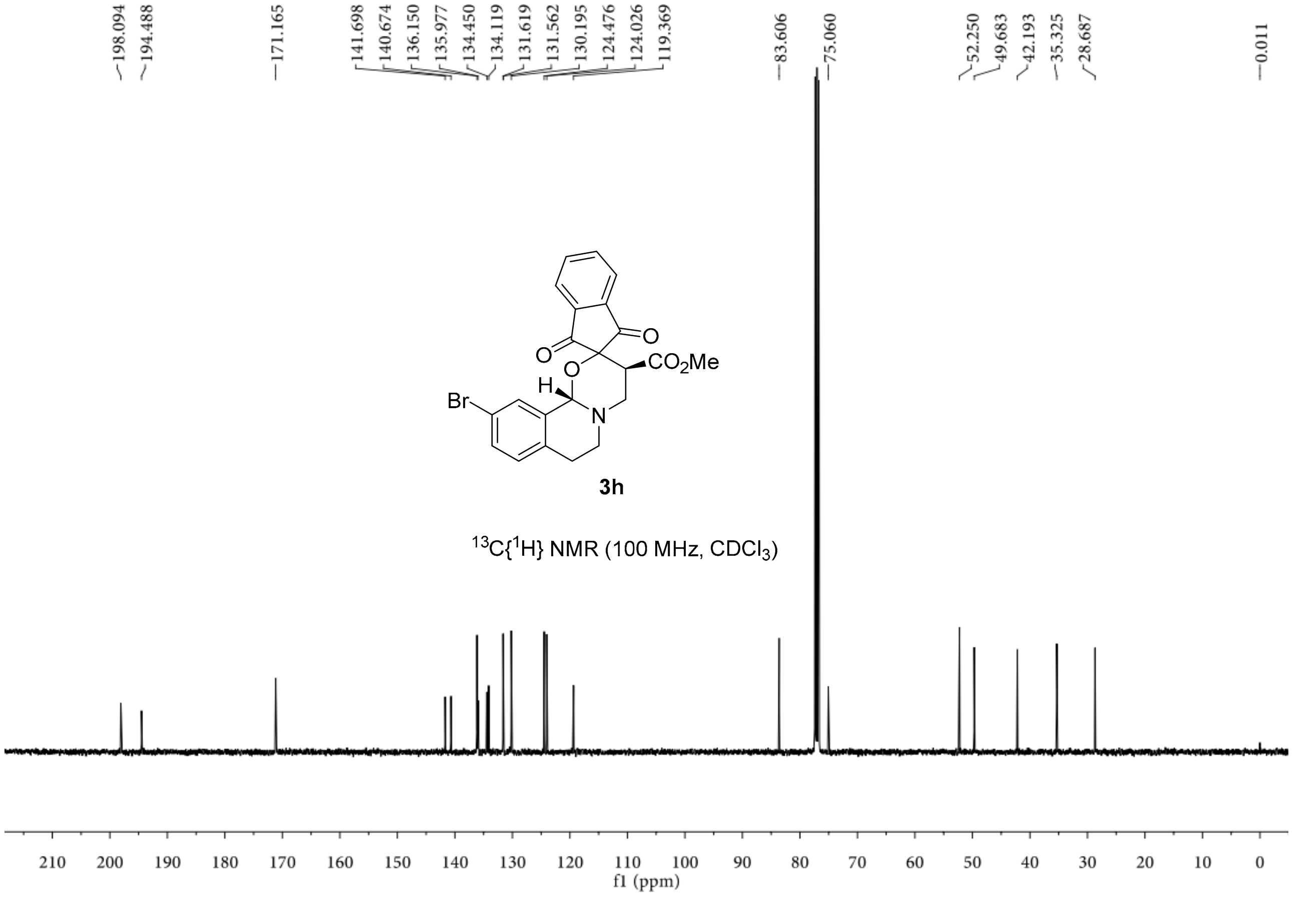
198.094
194.488
171.165
141.698
140.674
136.150
135.977
134.450
134.119
131.619
131.562
130.195
124.476
124.026
119.369
83.606
75.060
52.250
49.683
42.193
35.325
28.687
0.011
Br
H
O
N
CO_2Me
3h
$^{13}C\{^1H\}$ NMR (100 MHz, $CDCl_3$)
210
200
190
180
170
160
150
140
130
120
110
100
90
80
70
60
50
40
30
20
10
0
f1 (ppm)

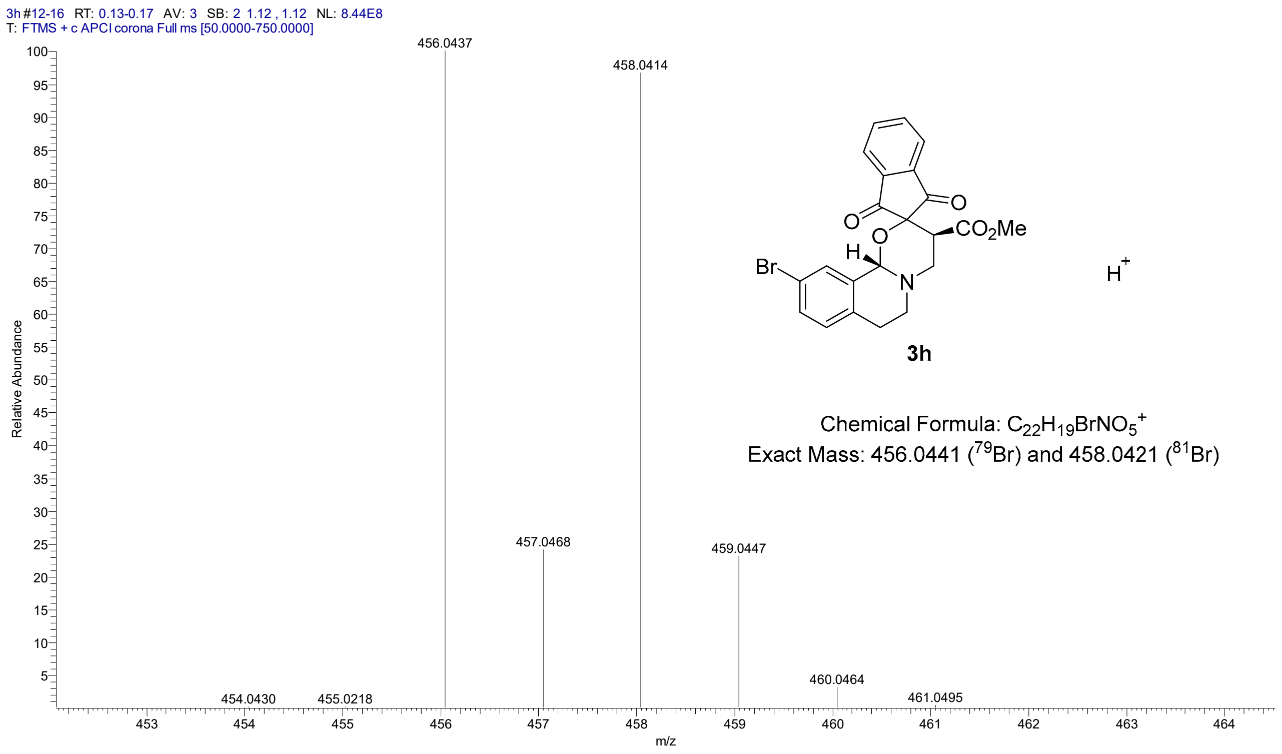

$[M + H]^+$ calcd for $C_{22}H_{19}BrNO_5$ 456.0441 (^{79}Br) and 458.0421 (^{81}Br), found 456.0437, 458.0414.

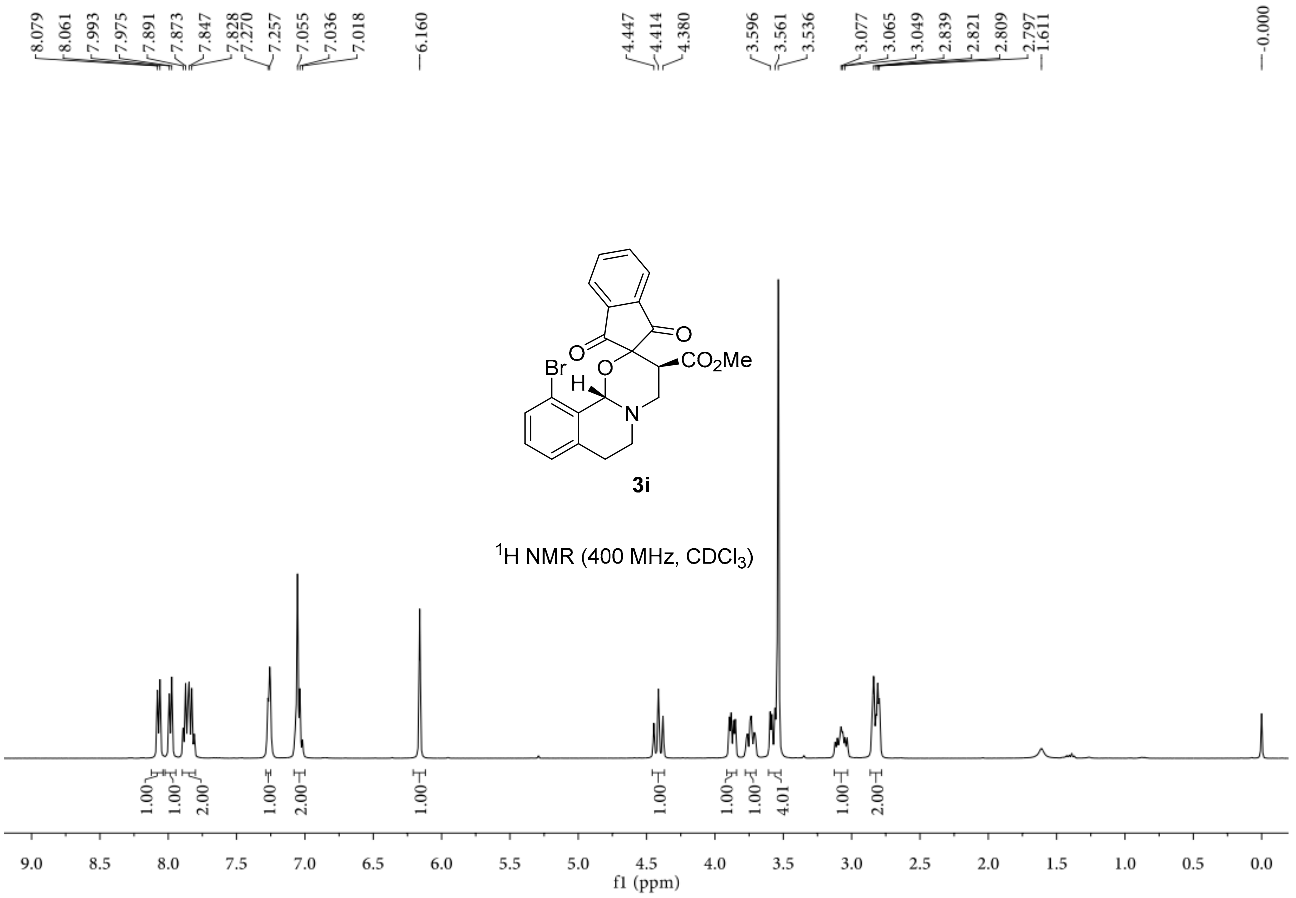
8.079 8.061 7.993 7.975 7.891 7.873 7.847 7.828 7.270 7.257 7.055 7.036 7.018
6.160
4.447 4.414 4.380
3.596 3.561 3.536
3.077 3.065 3.049 2.839 2.821 2.809 2.797 1.611
-0.000
Br
H
O
N
CO2Me
3i
1H NMR (400 MHz, CDCl3)
1.00 1.00 2.00 1.00 2.00 1.00 1.00 1.00 1.00 4.01 1.00 2.00
9.0 8.5 8.0 7.5 7.0 6.5 6.0 5.5 5.0 4.5 4.0 3.5 3.0 2.5 2.0 1.5 1.0 0.5 0.0
f1 (ppm)

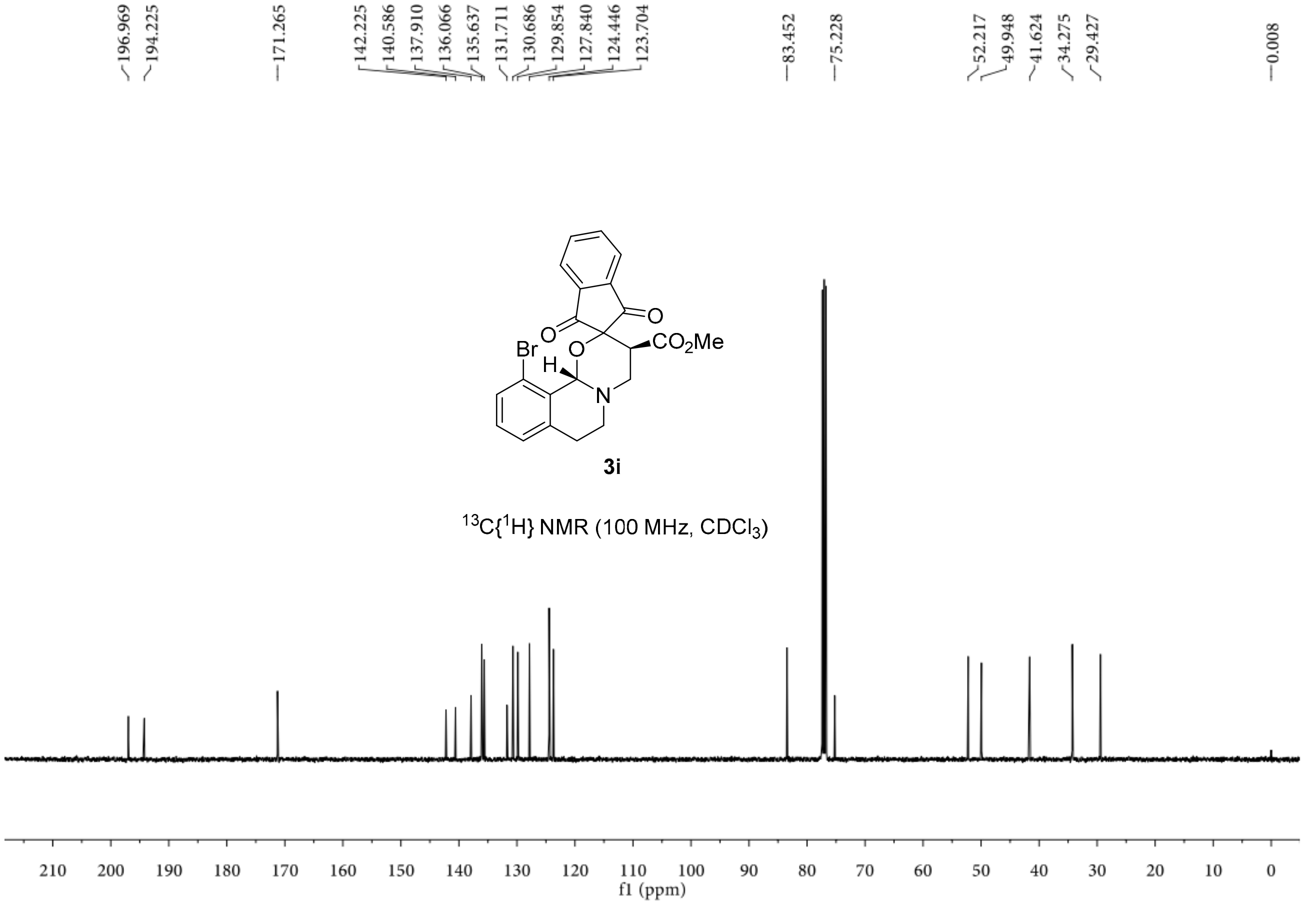
196.969 194.225
171.265
142.225 140.586 137.910 136.066 135.637 131.711 130.686 129.854 127.840 124.446 123.704
83.452
75.228
52.217 49.948 41.624 34.275 29.427
0.008
Br
H
O
N
CO2Me
3i
13C{1H} NMR (100 MHz, CDCl3)
210 200 190 180 170 160 150 140 130 120 110 100 90 80 70 60 50 40 30 20 10 0
f1 (ppm)

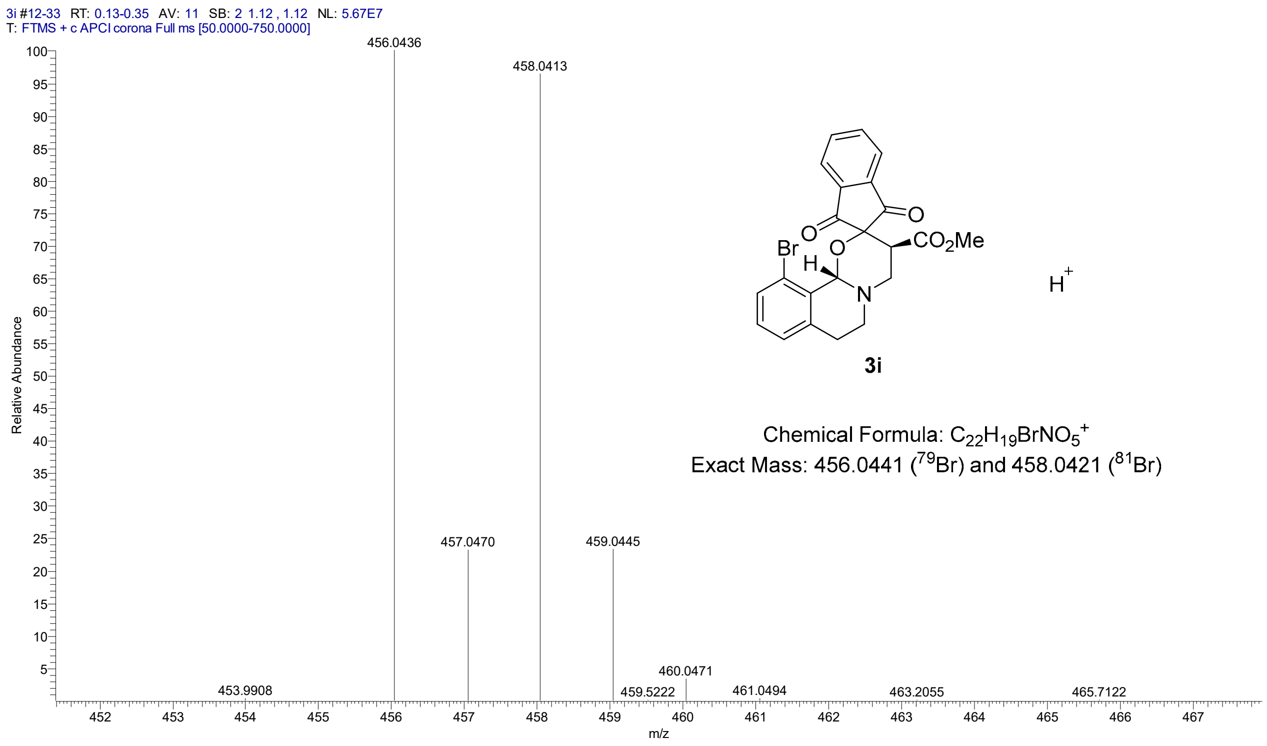

$[M + H]^+$ calcd for $C_{22}H_{19}BrNO_5$ 456.0441 (^{79}Br) and 458.0421 (^{81}Br), found 456.0436, 458.0413.

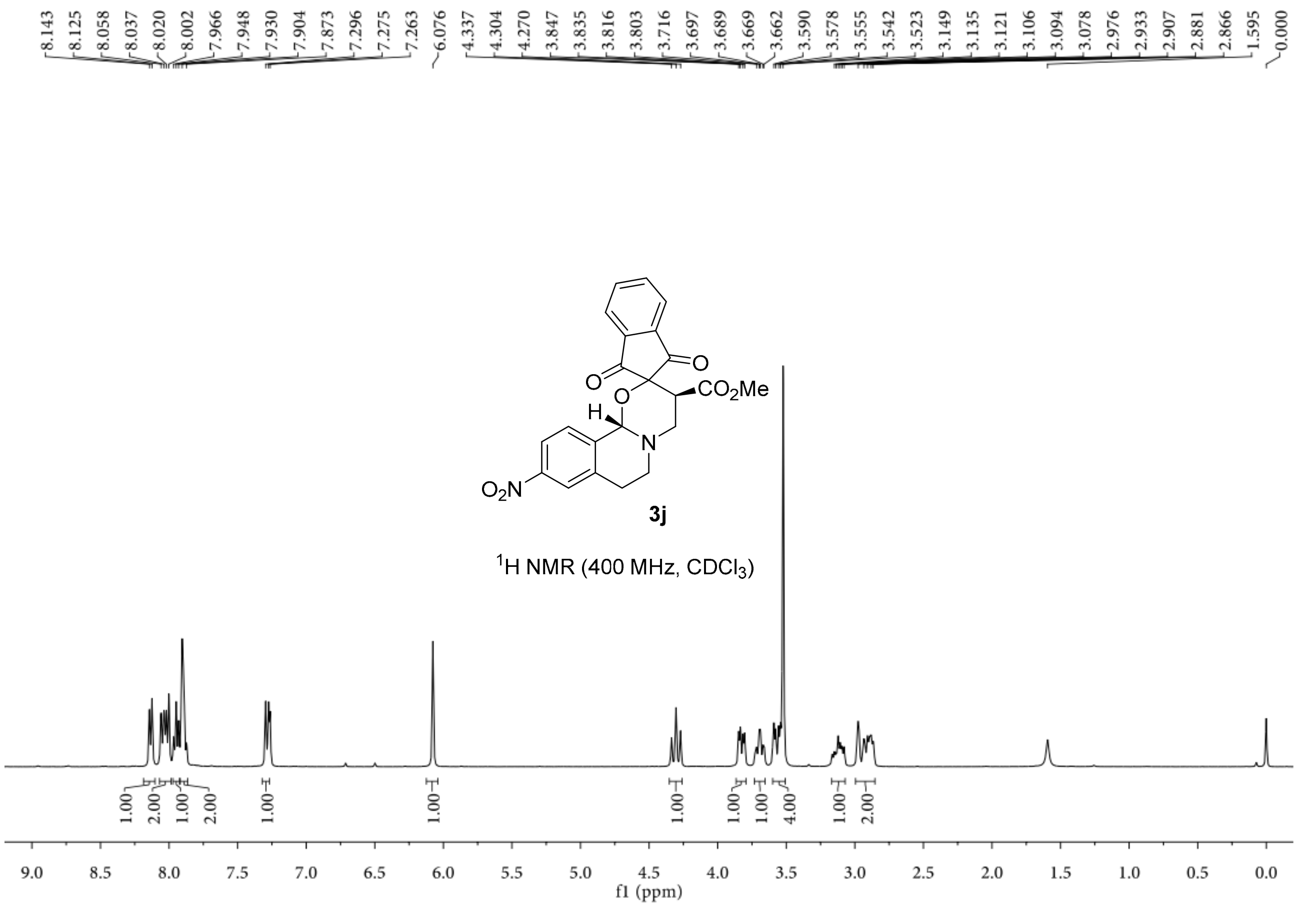
8.143 8.125 8.058 8.037 8.020 8.002 7.966 7.948 7.930 7.904 7.873 7.296 7.275 7.263 6.076 4.337 4.304 4.270 3.847 3.835 3.816 3.803 3.716 3.697 3.689 3.669 3.662 3.590 3.578 3.555 3.542 3.523 3.149 3.135 3.121 3.106 3.094 3.078 2.976 2.933 2.907 2.881 2.866 1.595 0.000
O
O
O
H
N
CO2Me
O2N
3j
1H NMR (400 MHz, CDCl3)
1.00 2.00 1.00 2.00 1.00 1.00 1.00 1.00 1.00 4.00 1.00 2.00
9.0 8.5 8.0 7.5 7.0 6.5 6.0 5.5 5.0 4.5 4.0 3.5 3.0 2.5 2.0 1.5 1.0 0.5 0.0
f1 (ppm)

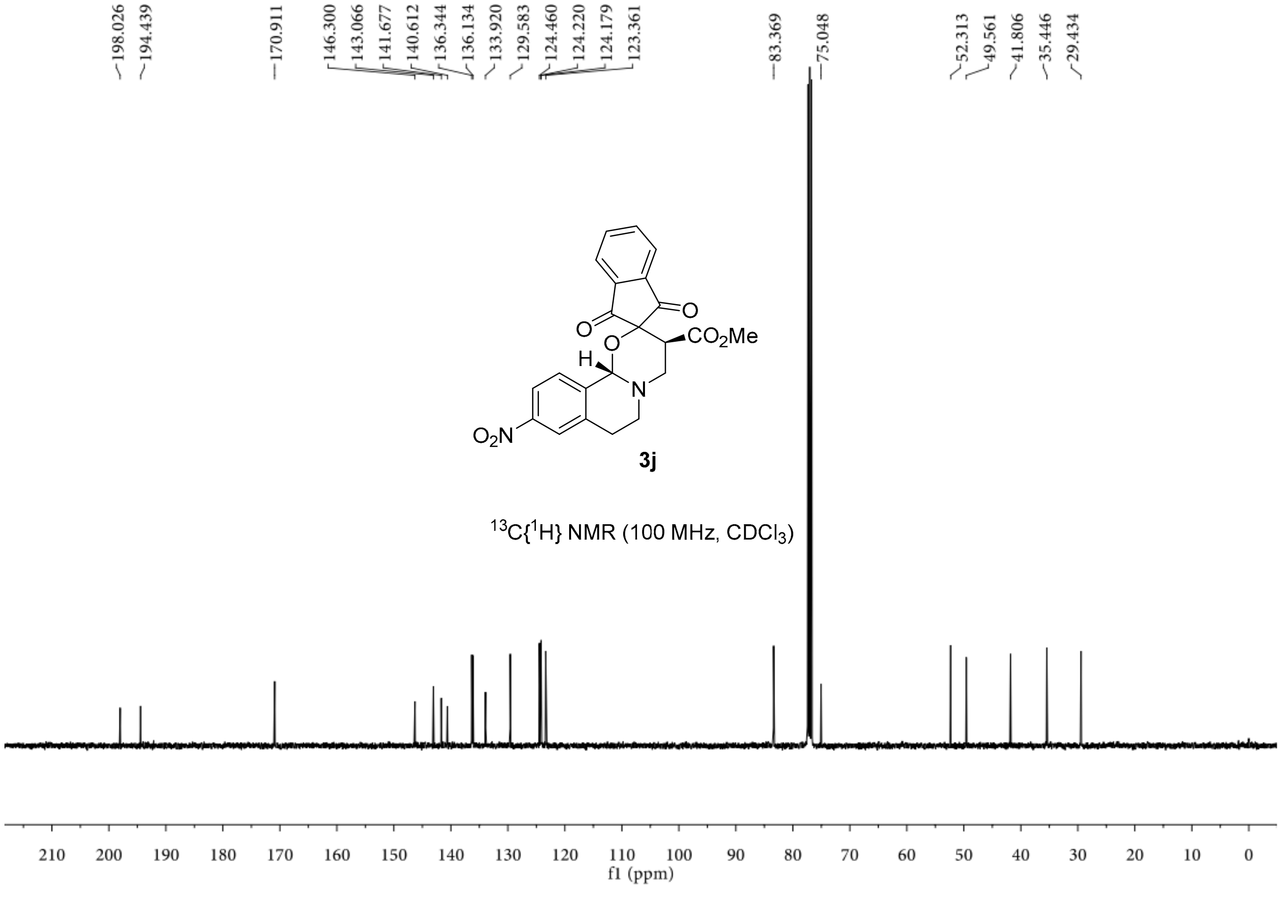
198.026 194.439 170.911 146.300 143.066 141.677 140.612 136.344 136.134 133.920 129.583 124.460 124.220 124.179 123.361 83.369 75.048 52.313 49.561 41.806 35.446 29.434
O
O
O
H
N
CO2Me
O2N
3j
13C{1H} NMR (100 MHz, CDCl3)
210 200 190 180 170 160 150 140 130 120 110 100 90 80 70 60 50 40 30 20 10 0
f1 (ppm)

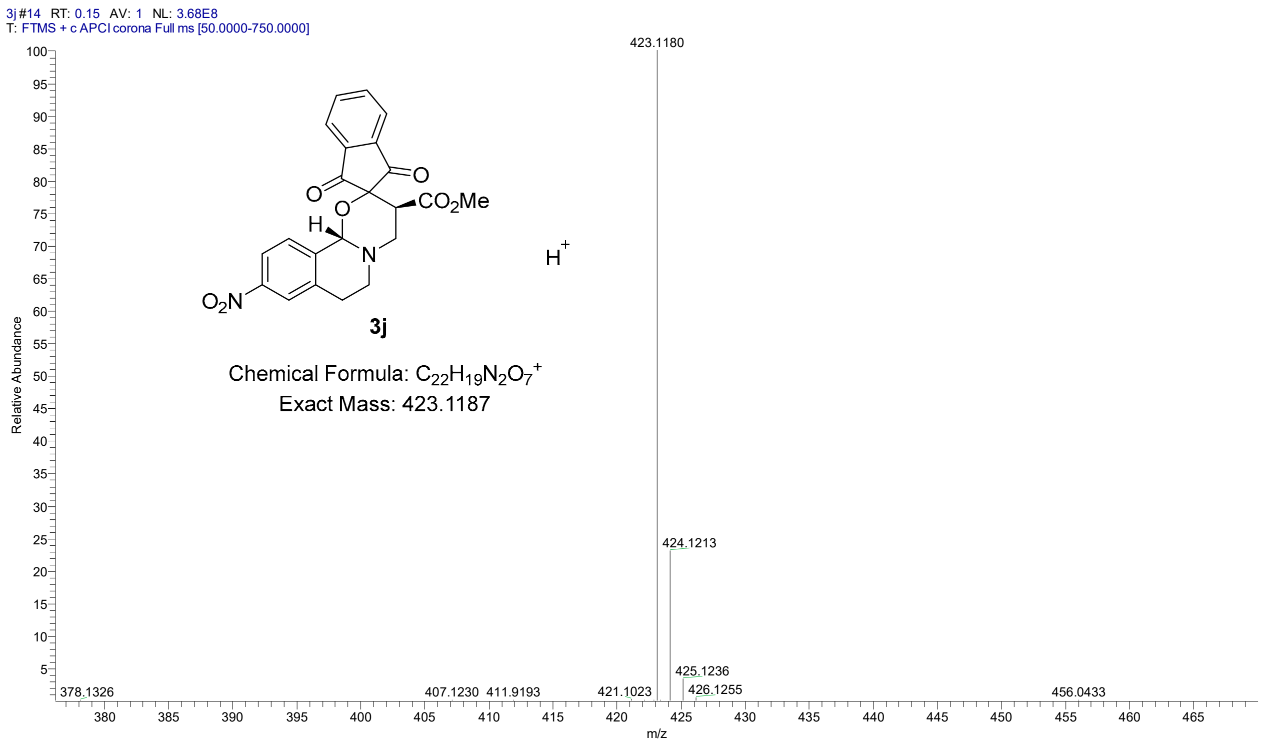

$[M + H]^+$ calcd for $C_{22}H_{19}N_2O_7$ 423.1187, found 423.1180.

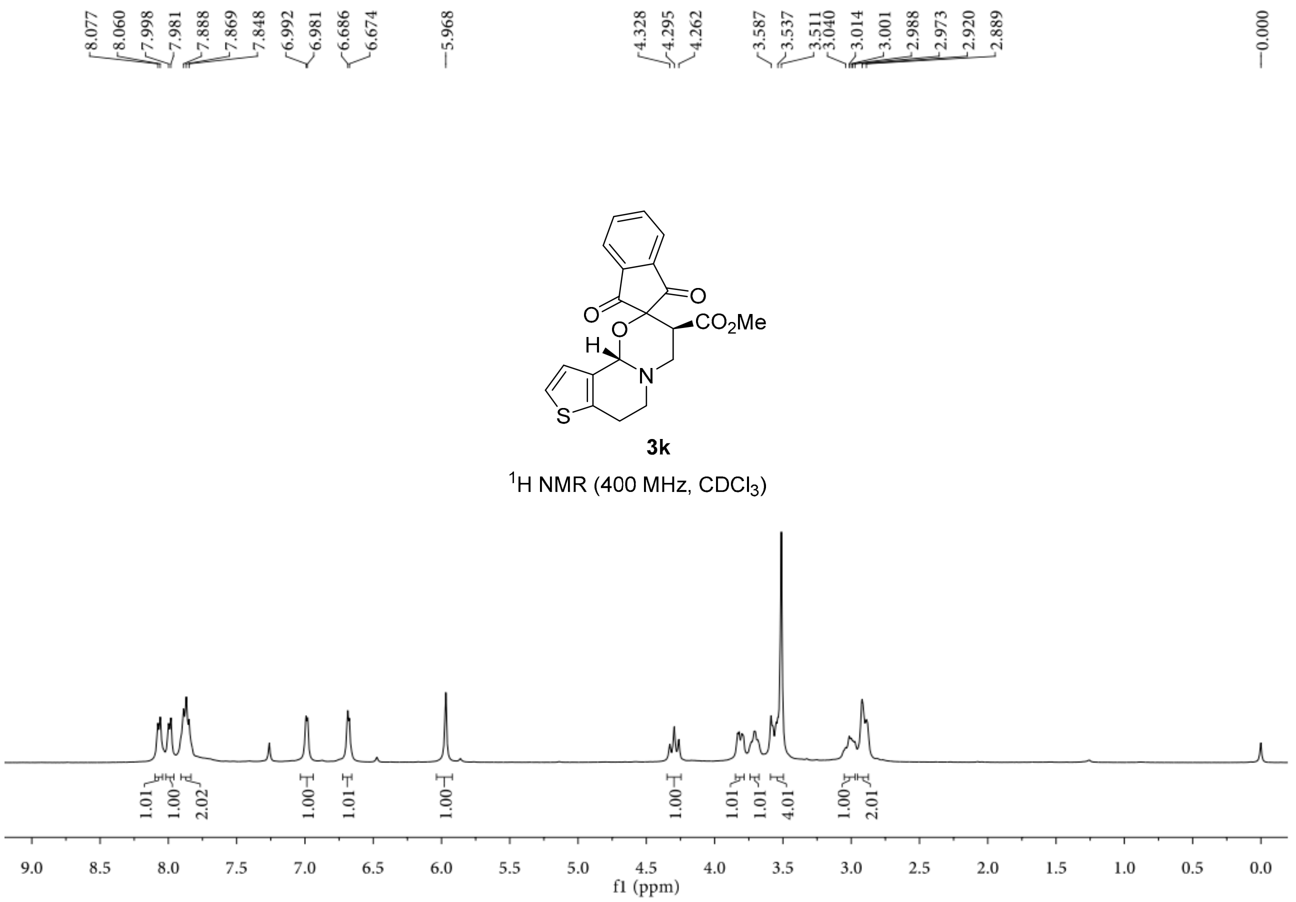

3k

^1H NMR (400 MHz, $CDCl_3$)

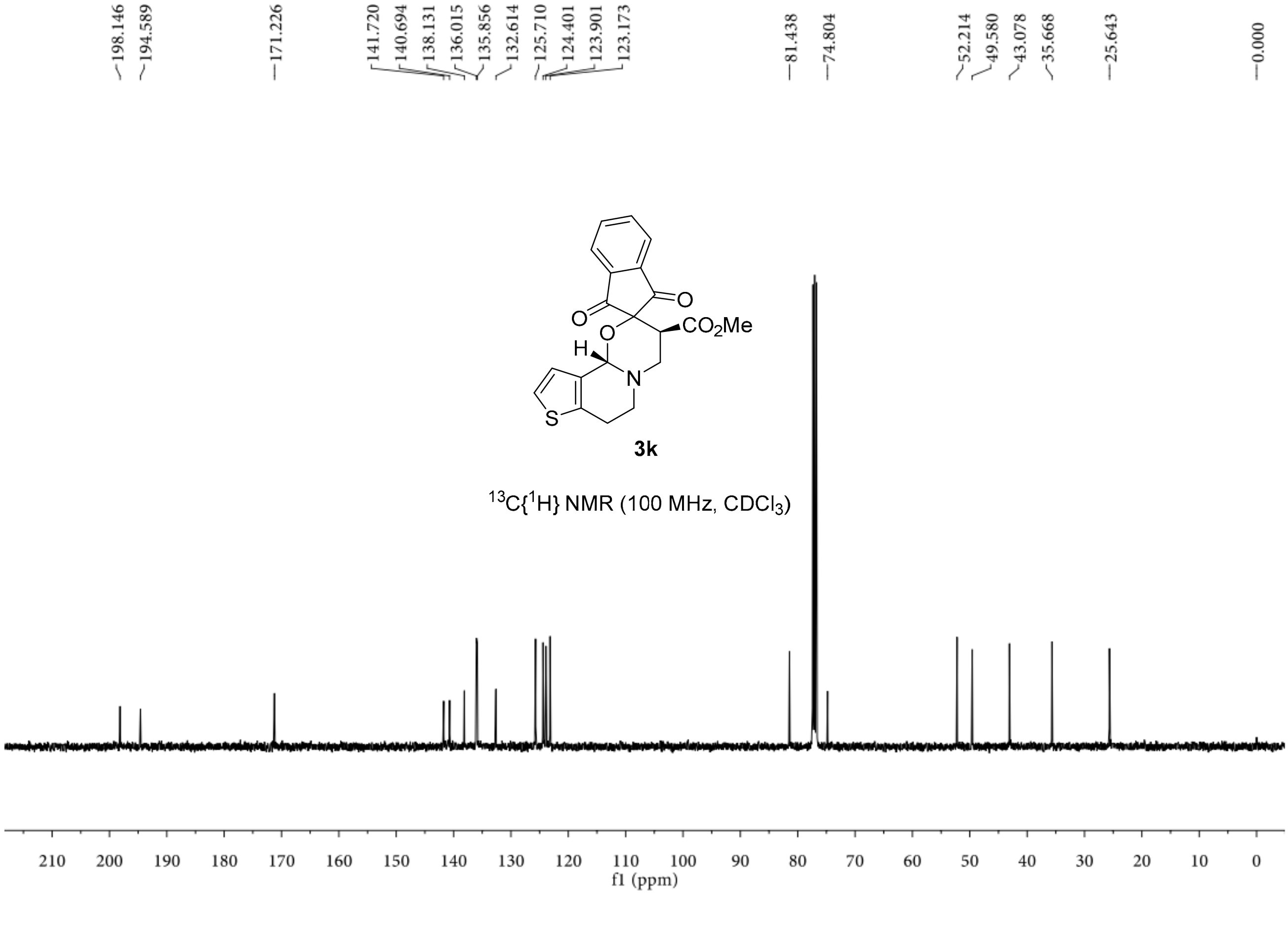

3k

$^{13}C\{^1H\}$ NMR (100 MHz, $CDCl_3$)

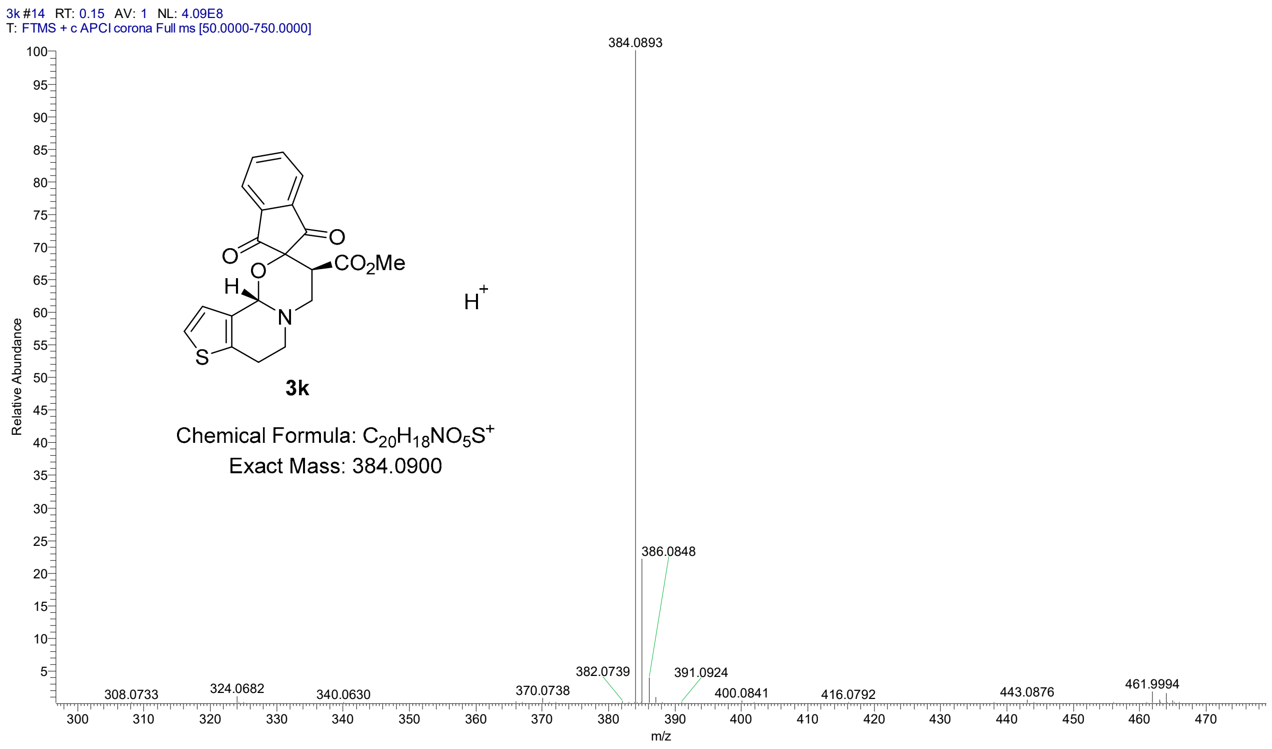

[M + H]+ calcd for $C_{20}H_{18}NO_5S$ 384.0900, found 384.0893.

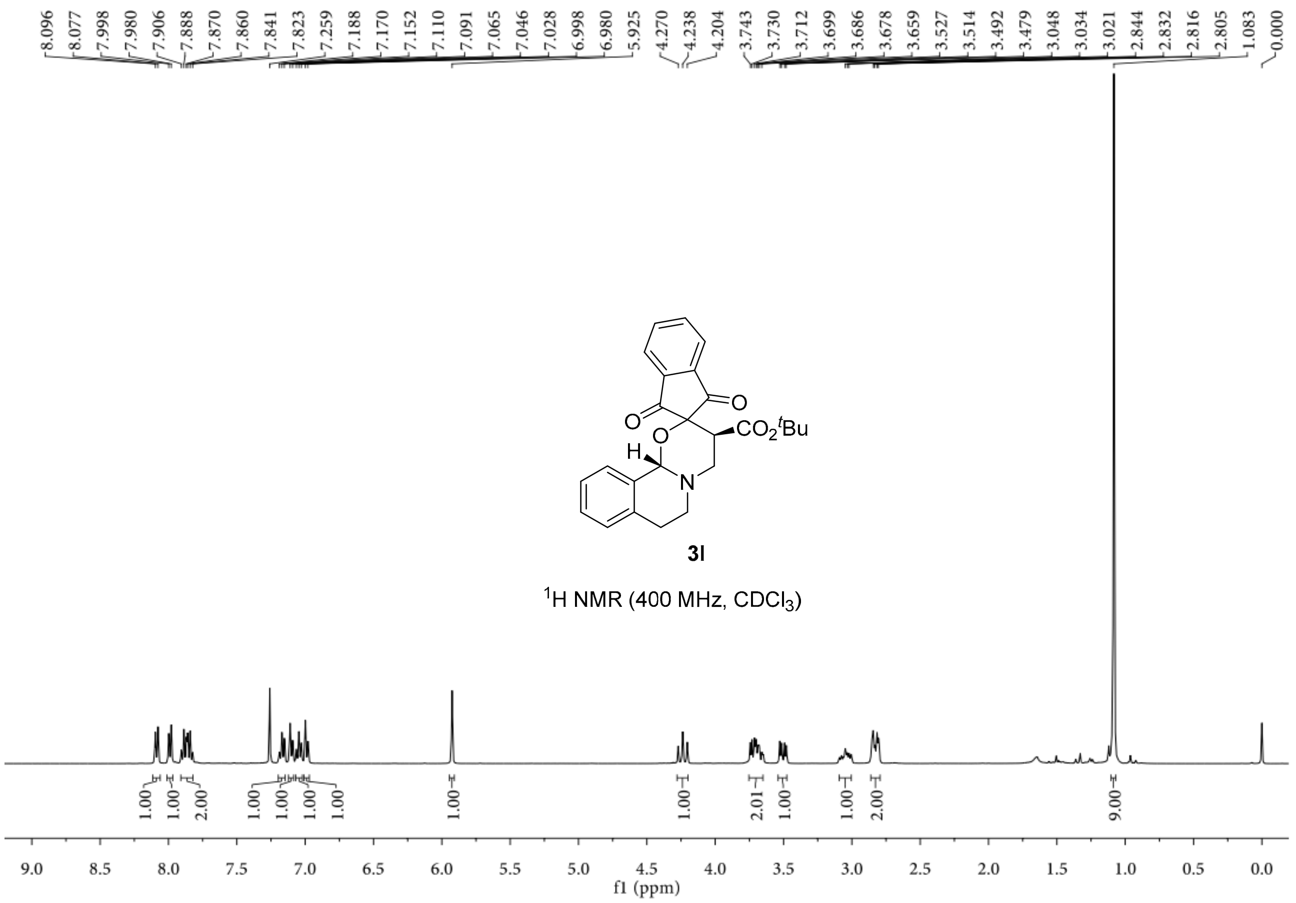
8.096 8.077 7.998 7.980 7.906 7.888 7.870 7.860 7.841 7.823 7.259 7.188 7.170 7.152 7.110 7.091 7.065 7.046 7.028 6.998 6.980 5.925 4.270 4.238 4.204 3.743 3.730 3.712 3.699 3.686 3.678 3.659 3.527 3.514 3.492 3.479 3.048 3.034 3.021 2.844 2.832 2.816 2.805 1.083 0.000
CO2tBu
3l
1H NMR (400 MHz, CDCl3)
1.00 1.00 2.00 1.00 1.00 1.00 1.00 1.00 1.00 2.01 1.00 1.00 2.00 9.00
f1 (ppm)

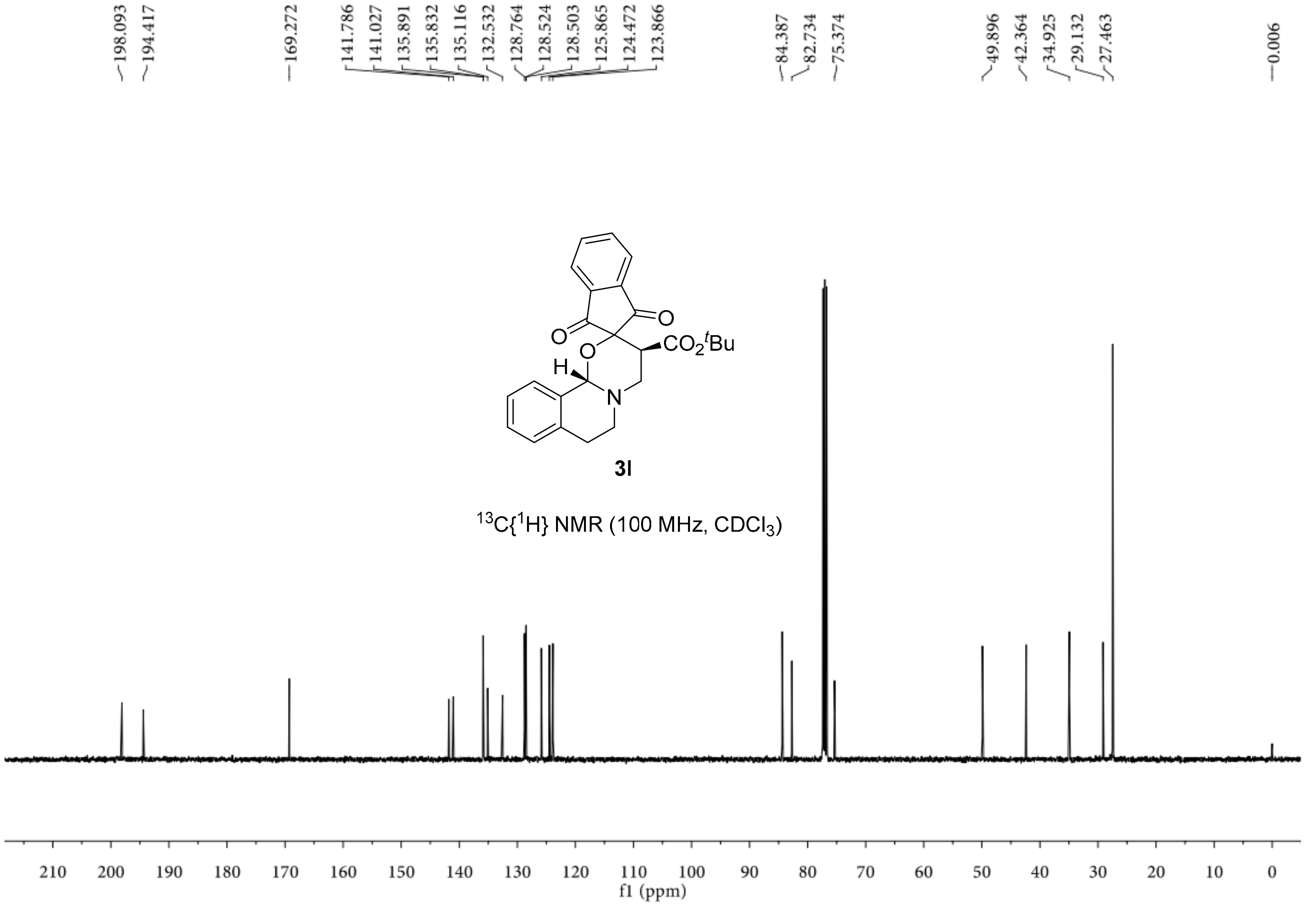
198.093 194.417 169.272 141.786 141.027 135.891 135.832 135.116 132.532 128.764 128.524 128.503 125.865 124.472 123.866 84.387 82.734 75.374 49.896 42.364 34.925 29.132 27.463 0.006
CO2tBu
3l
13C{1H} NMR (100 MHz, CDCl3)
f1 (ppm)

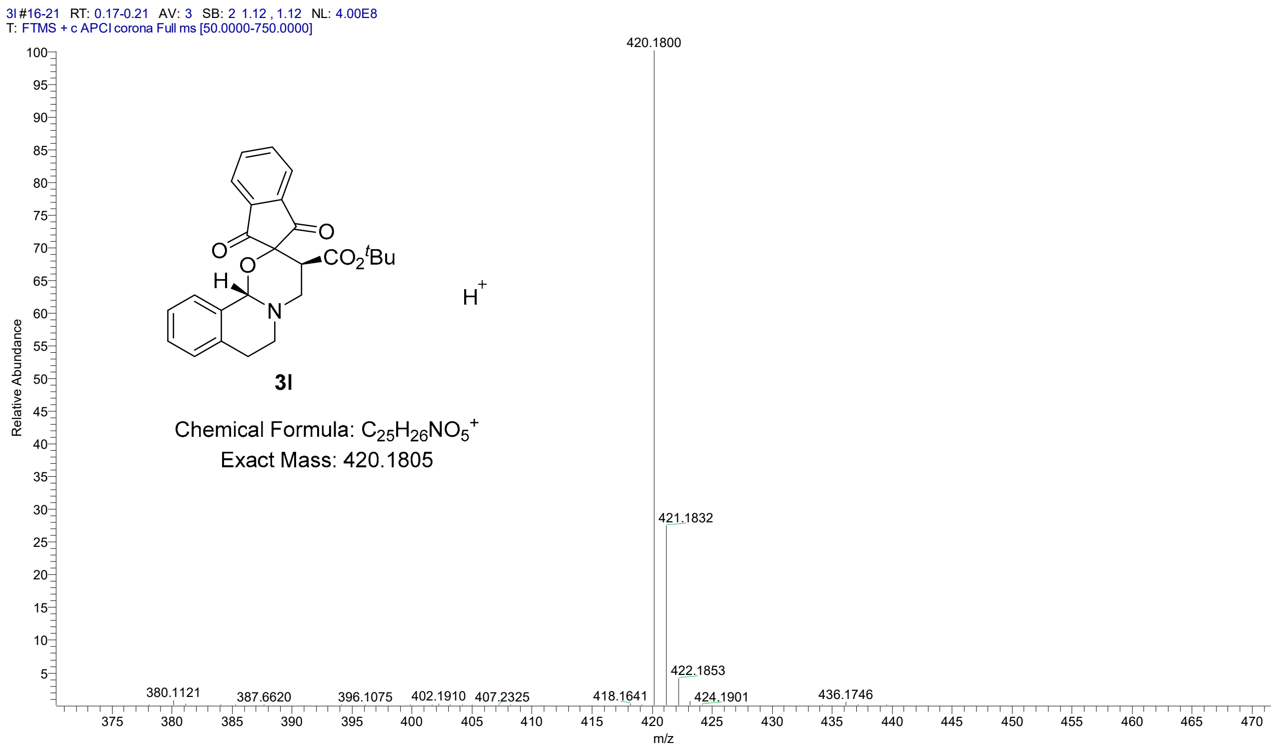

$[M + H]^+$ calcd for $C_{25}H_{26}NO_5$ 420.1805, found 420.1800.

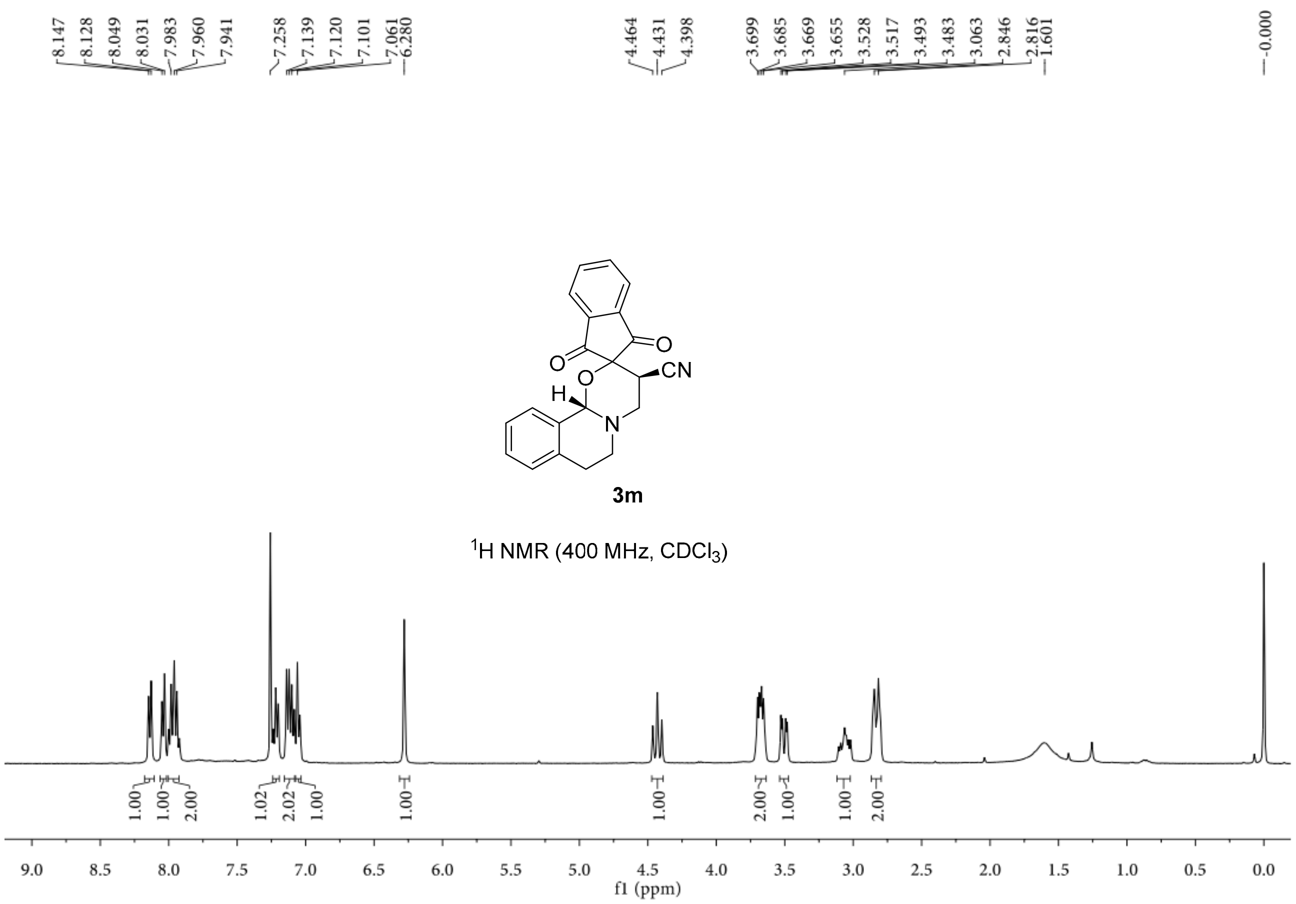
8.147 8.128 8.049 8.031 7.983 7.960 7.941 7.258 7.139 7.120 7.101 7.061 6.280 4.464 4.431 4.398 3.699 3.685 3.669 3.655 3.528 3.517 3.493 3.483 3.063 2.846 2.816 1.601 -0.000
O
O
CN
H
O
N
3m
1H NMR (400 MHz, CDCl3)
1.00 1.00 2.00 1.02 2.02 1.00 1.00 1.00 2.00 1.00 1.00 2.00
f1 (ppm)

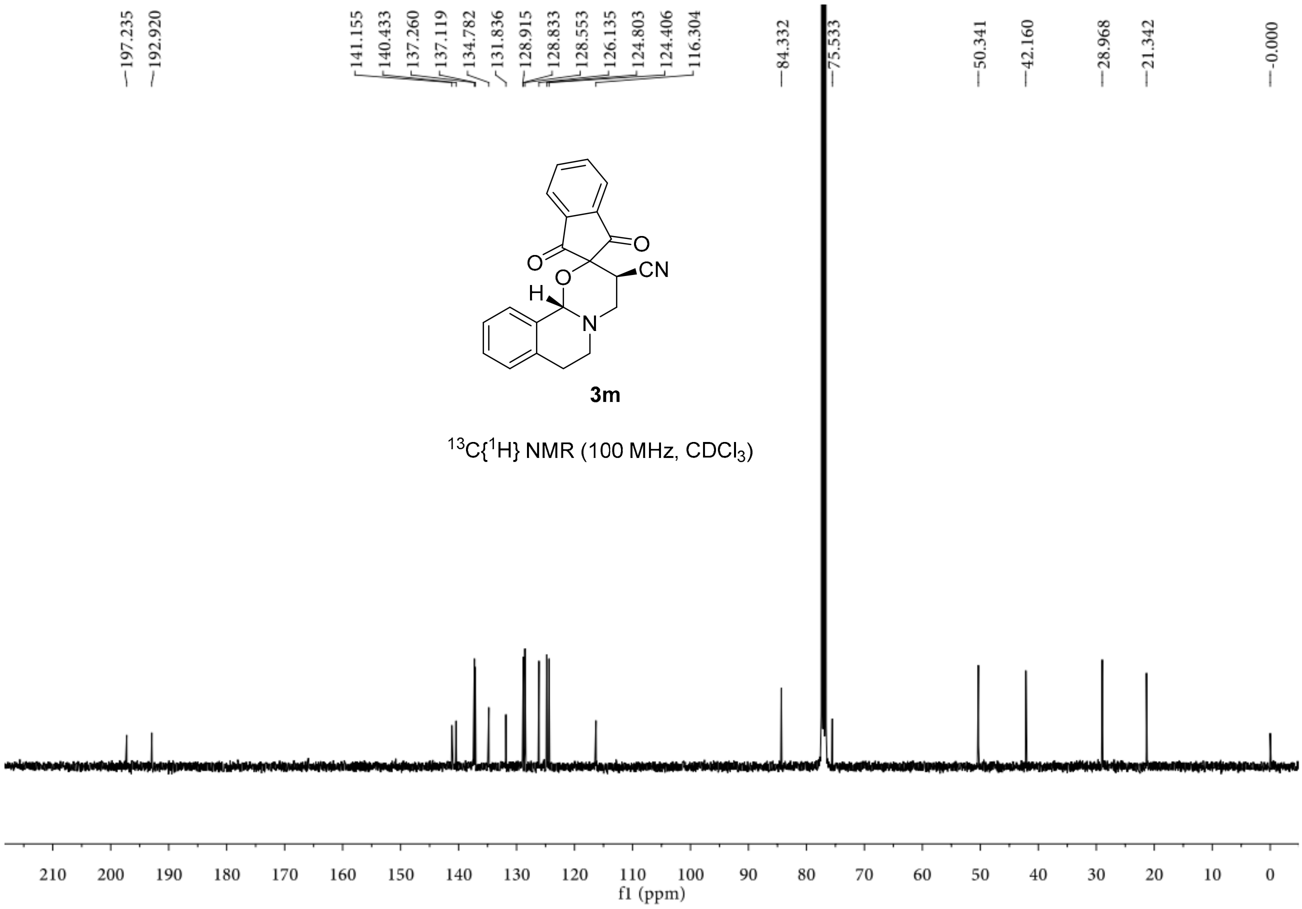
197.235 192.920 141.155 140.433 137.260 137.119 134.782 131.836 128.915 128.833 128.553 126.135 124.803 124.406 116.304 84.332 75.533 50.341 42.160 28.968 21.342 -0.000
O
O
CN
H
O
N
3m
13C{1H} NMR (100 MHz, CDCl3)
f1 (ppm)

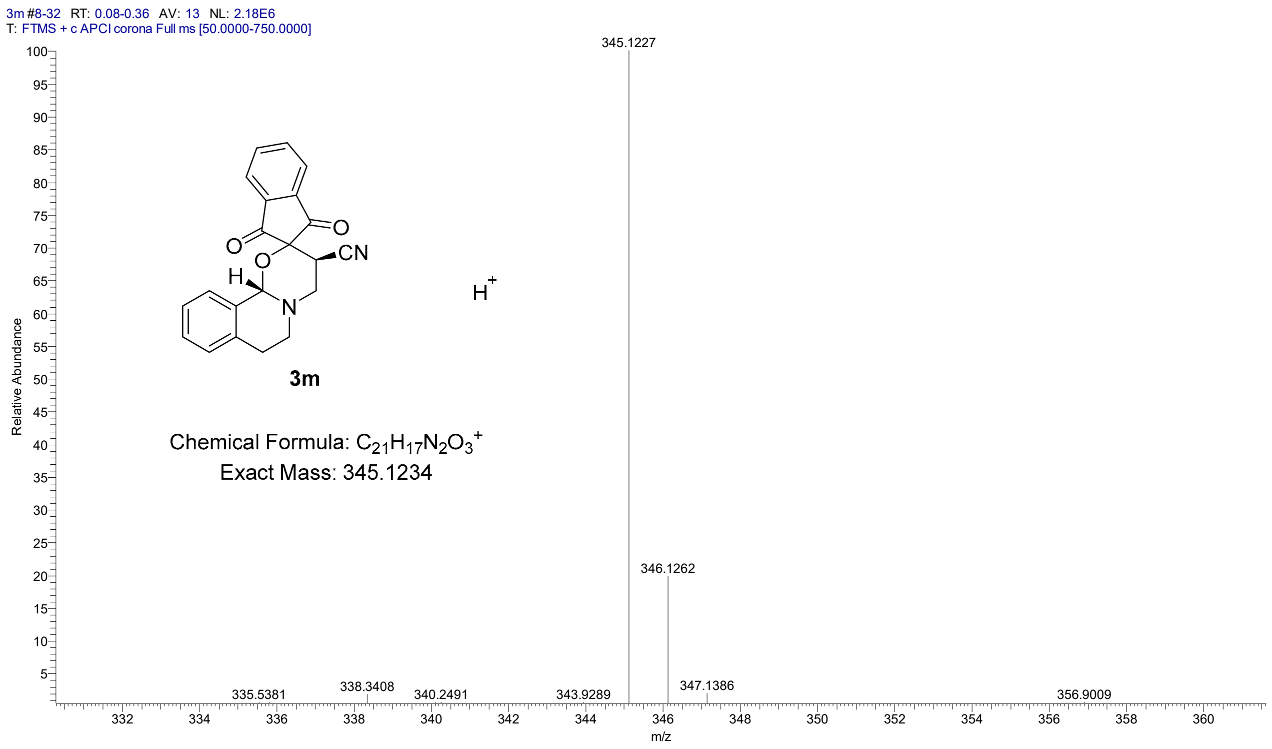

[M + H]+ calcd for $C_{21}H_{17}N_2O_3$ 345.1234, found 345.1227.

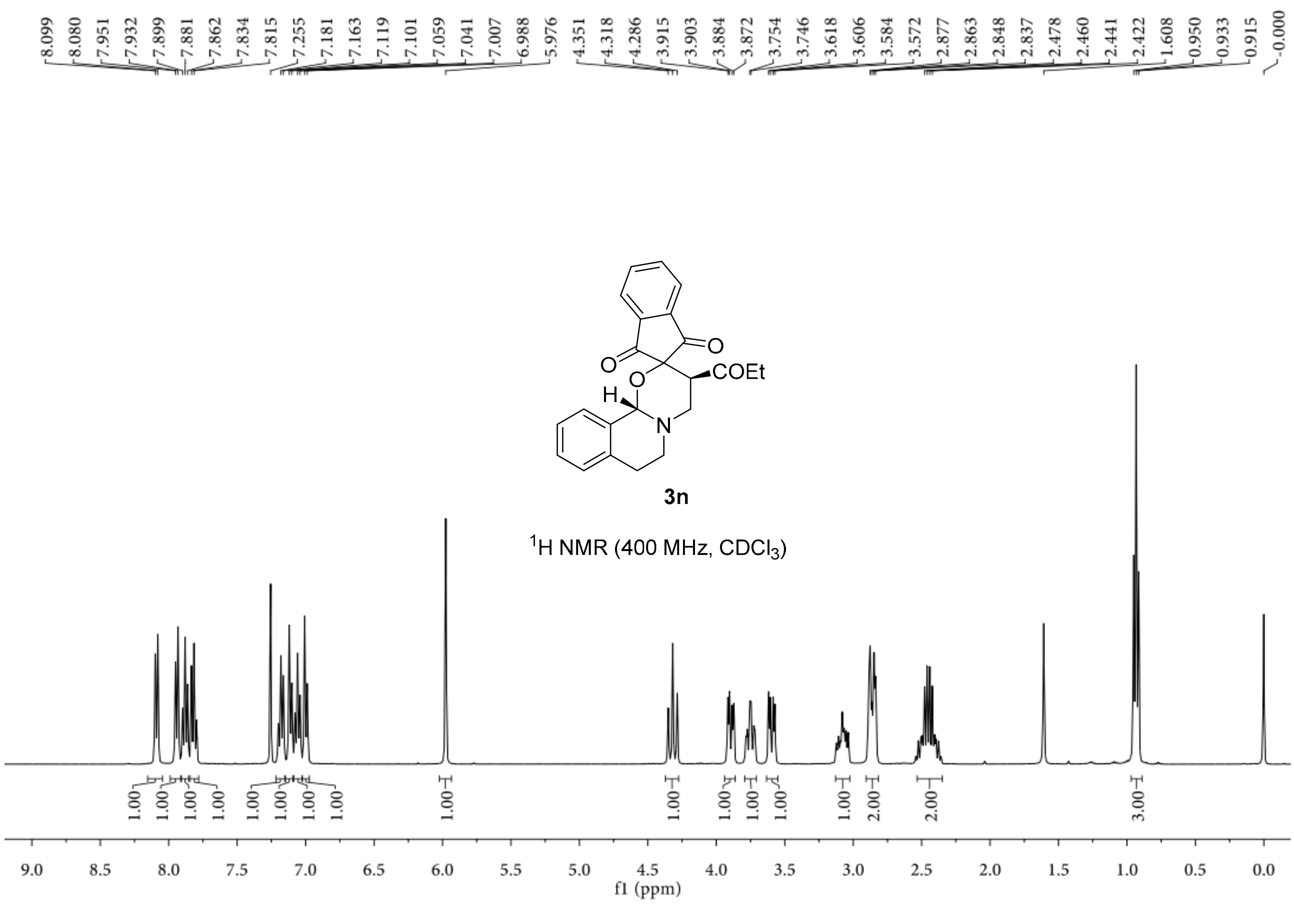

8.099 8.080 7.951 7.932 7.899 7.881 7.862 7.834 7.815 7.255 7.181 7.163 7.119 7.101 7.059 7.041 7.007 6.988 5.976 4.351 4.318 4.286 3.915 3.903 3.884 3.872 3.754 3.746 3.618 3.606 3.584 3.572 2.877 2.863 2.848 2.837 2.478 2.460 2.441 2.422 1.608 0.950 0.933 0.915 -0.000
COEt
H
O
N
3n
1H NMR (400 MHz, CDCl3)
1.00 1.00 1.00 1.00 1.00 1.00 1.00 1.00 1.00 1.00 1.00 1.00 1.00 1.00 2.00 2.00 3.00
f1 (ppm)

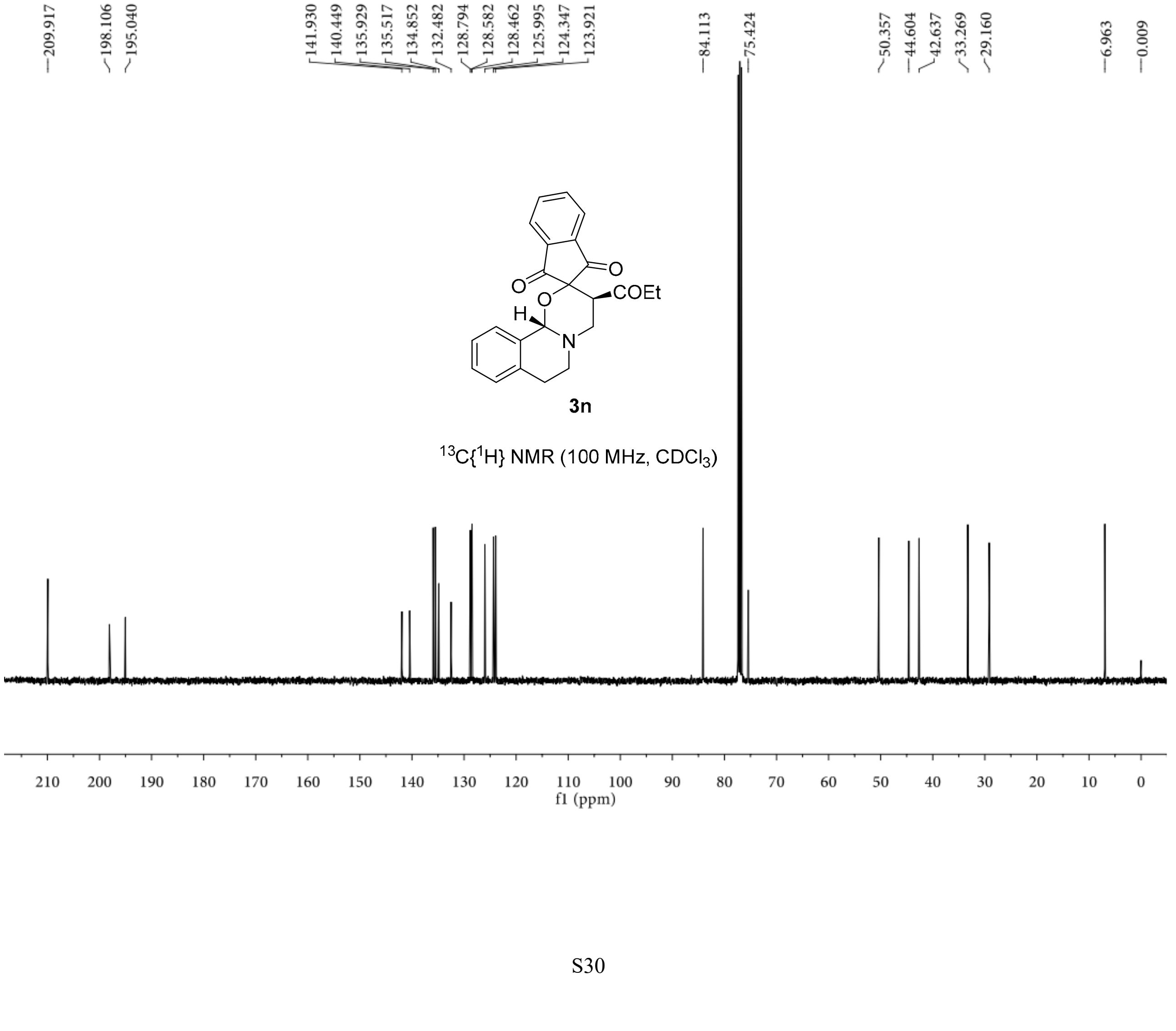

209.917 198.106 195.040 141.930 140.449 135.929 135.517 134.852 132.482 128.794 128.582 128.462 125.995 124.347 123.921 84.113 75.424 50.357 44.604 42.637 33.269 29.160 6.963 0.009
COEt
H
O
N
3n
13C{1H} NMR (100 MHz, CDCl3)
f1 (ppm)

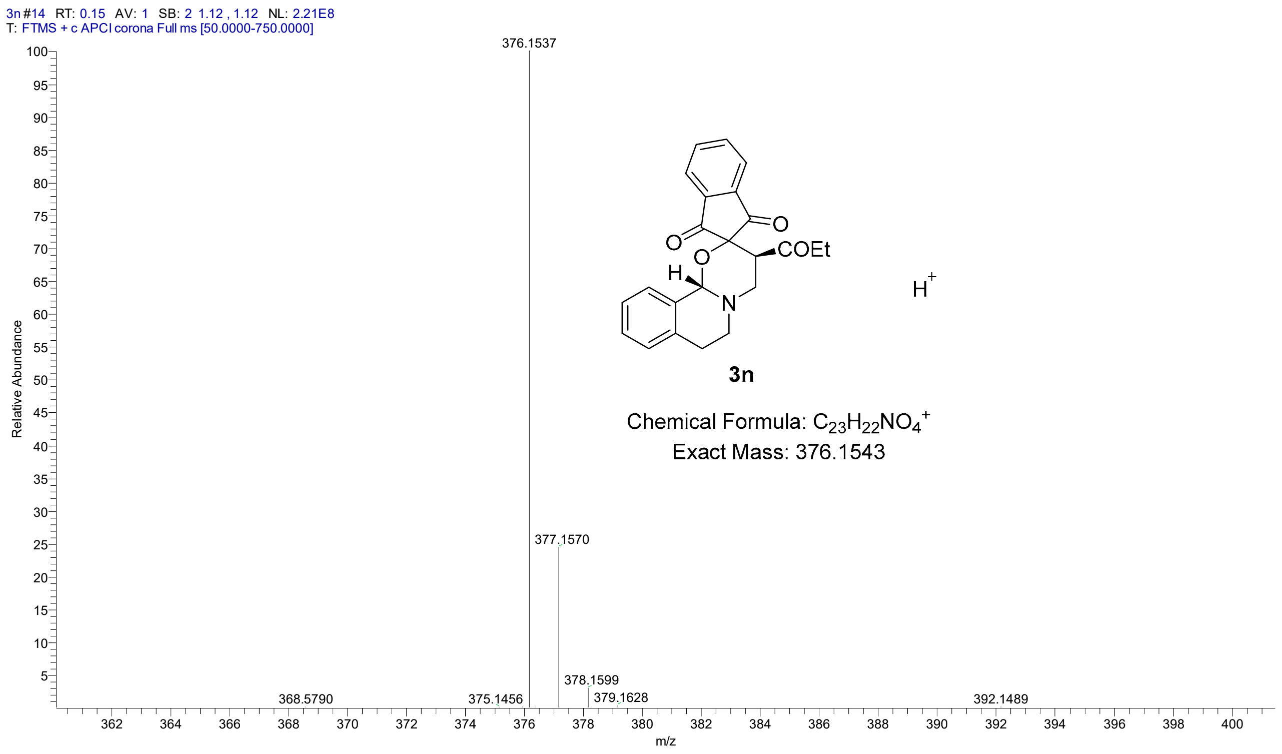

[M + H]$^+$ calcd for $C_{23}H_{22}NO_4$ 376.1543, found 376.1537.

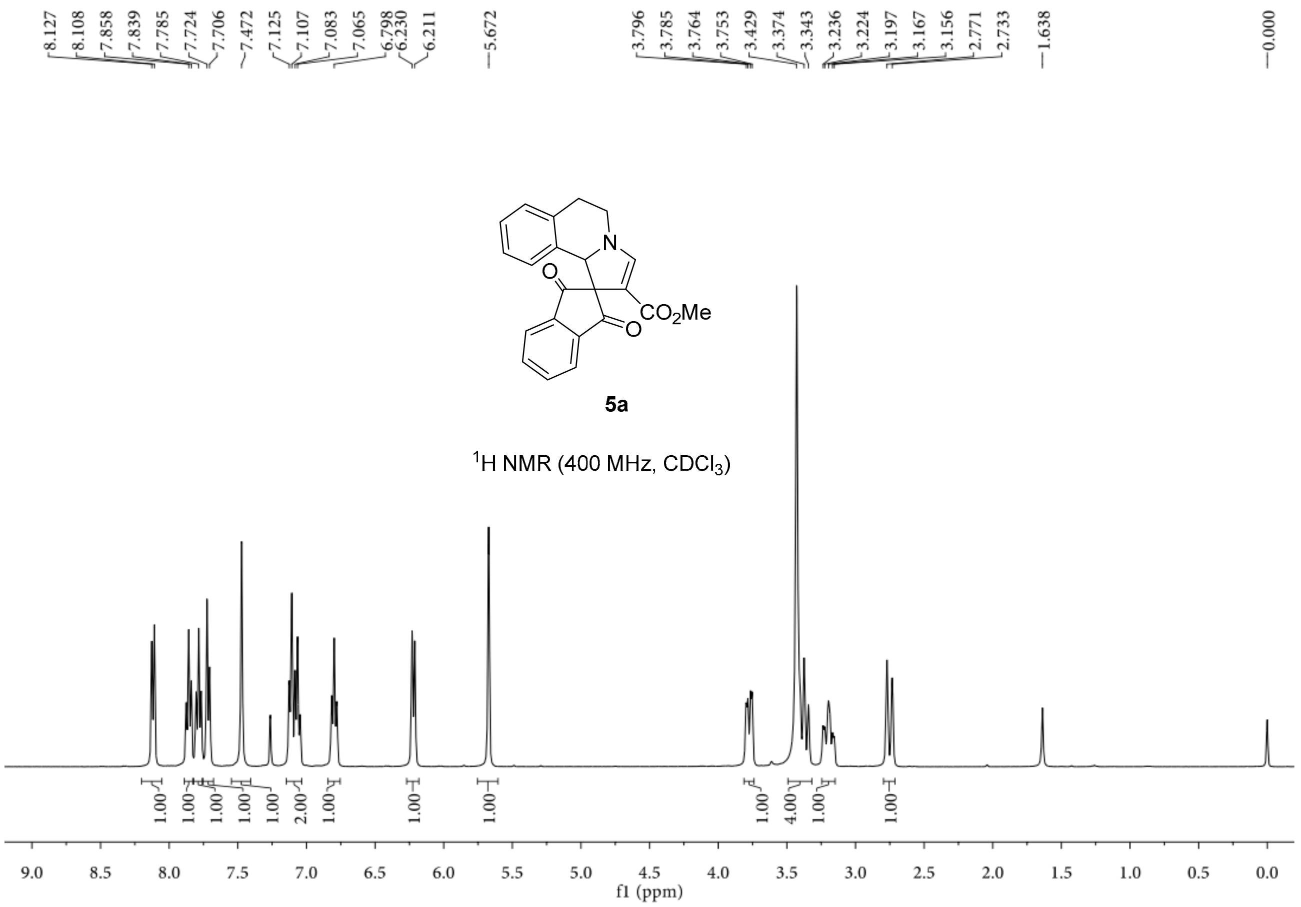
8.127
8.108
7.858
7.839
7.785
7.724
7.706
7.472
7.125
7.107
7.083
7.065
6.798
6.230
6.211
5.672
3.796
3.785
3.764
3.753
3.429
3.374
3.343
3.236
3.224
3.197
3.167
3.156
2.771
2.733
1.638
0.000
N
O
CO2Me
O
5a
1H NMR (400 MHz, CDCl3)
1.00
1.00
1.00
1.00
1.00
2.00
1.00
1.00
1.00
1.00
4.00
1.00
1.00
9.0 8.5 8.0 7.5 7.0 6.5 6.0 5.5 5.0 4.5 4.0 3.5 3.0 2.5 2.0 1.5 1.0 0.5 0.0
f1 (ppm)

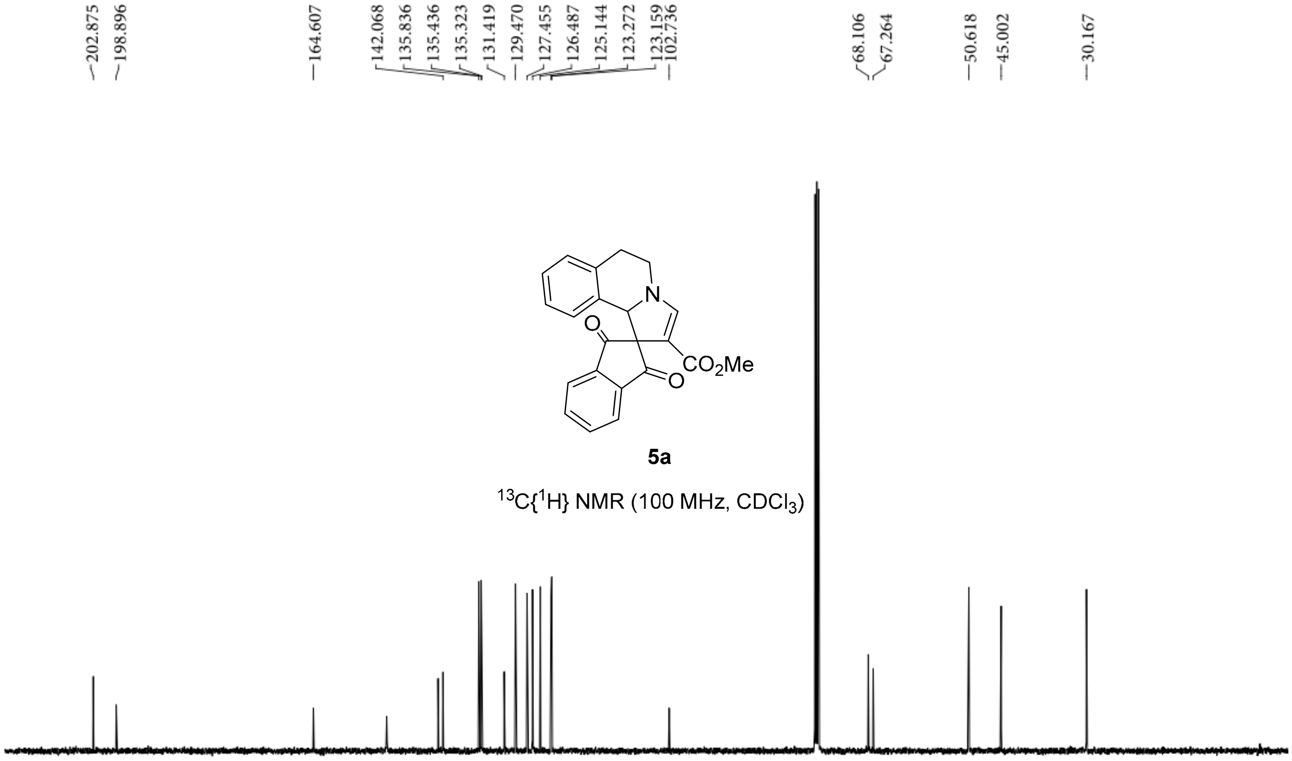
202.875
198.896
164.607
142.068
135.836
135.436
135.323
131.419
129.470
127.455
126.487
125.144
123.272
123.159
102.736
68.106
67.264
50.618
45.002
30.167
N
O
CO2Me
O
5a
13C{1H} NMR (100 MHz, CDCl3)
210 200 190 180 170 160 150 140 130 120 110 100 90 80 70 60 50 40 30 20 10 0
f1 (ppm)

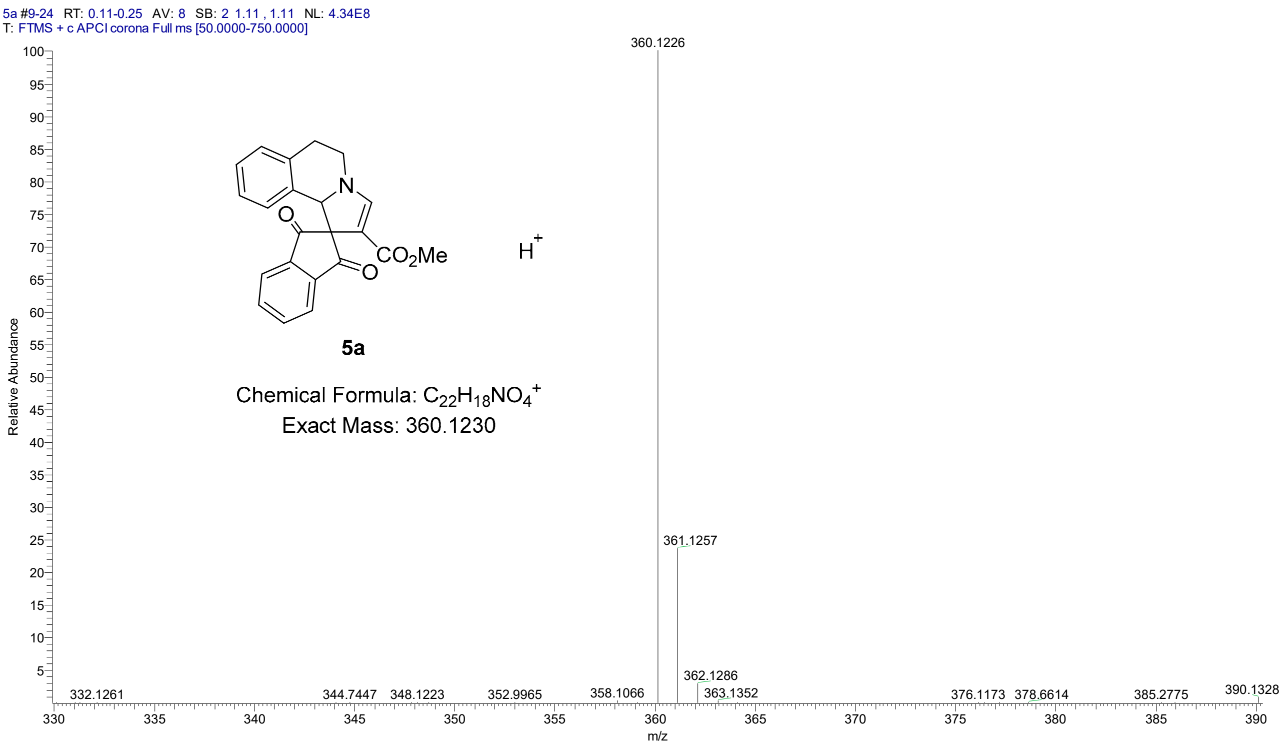

[M + H]$^+$ calcd for $C_{22}H_{18}NO_4$ 360.1230, found 360.1226.

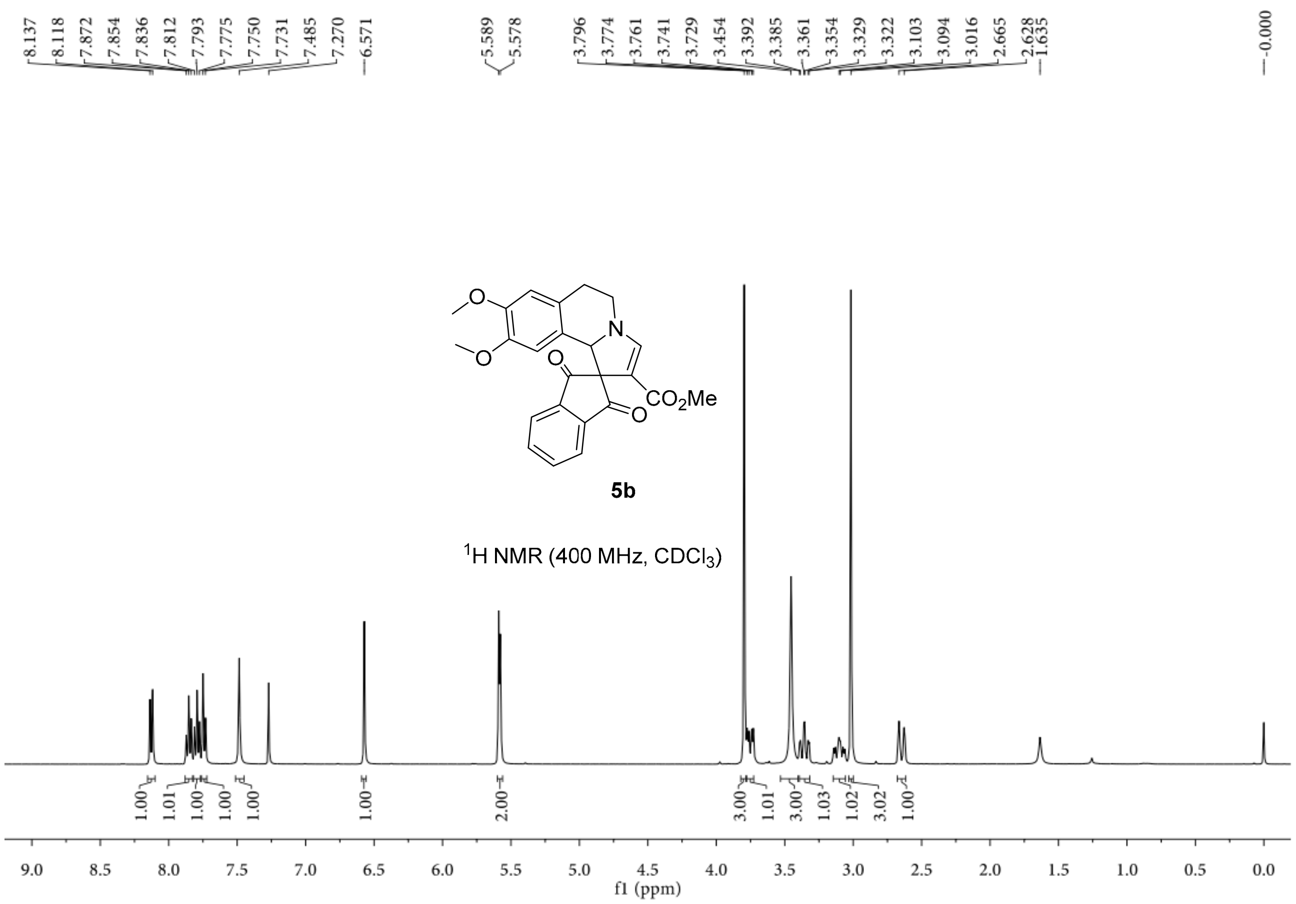
8.137 8.118 7.872 7.854 7.836 7.812 7.793 7.775 7.750 7.731 7.485 7.270 6.571
5.589 5.578
3.796 3.774 3.761 3.741 3.729 3.454 3.392 3.385 3.361 3.354 3.329 3.322 3.103 3.094 3.016 2.665 2.628 1.635
-0.000
CO2Me
5b
1H NMR (400 MHz, CDCl3)
1.00 1.01 1.00 1.00 1.00 1.00 2.00 3.00 1.01 3.00 1.03 1.02 3.02 1.00
f1 (ppm)

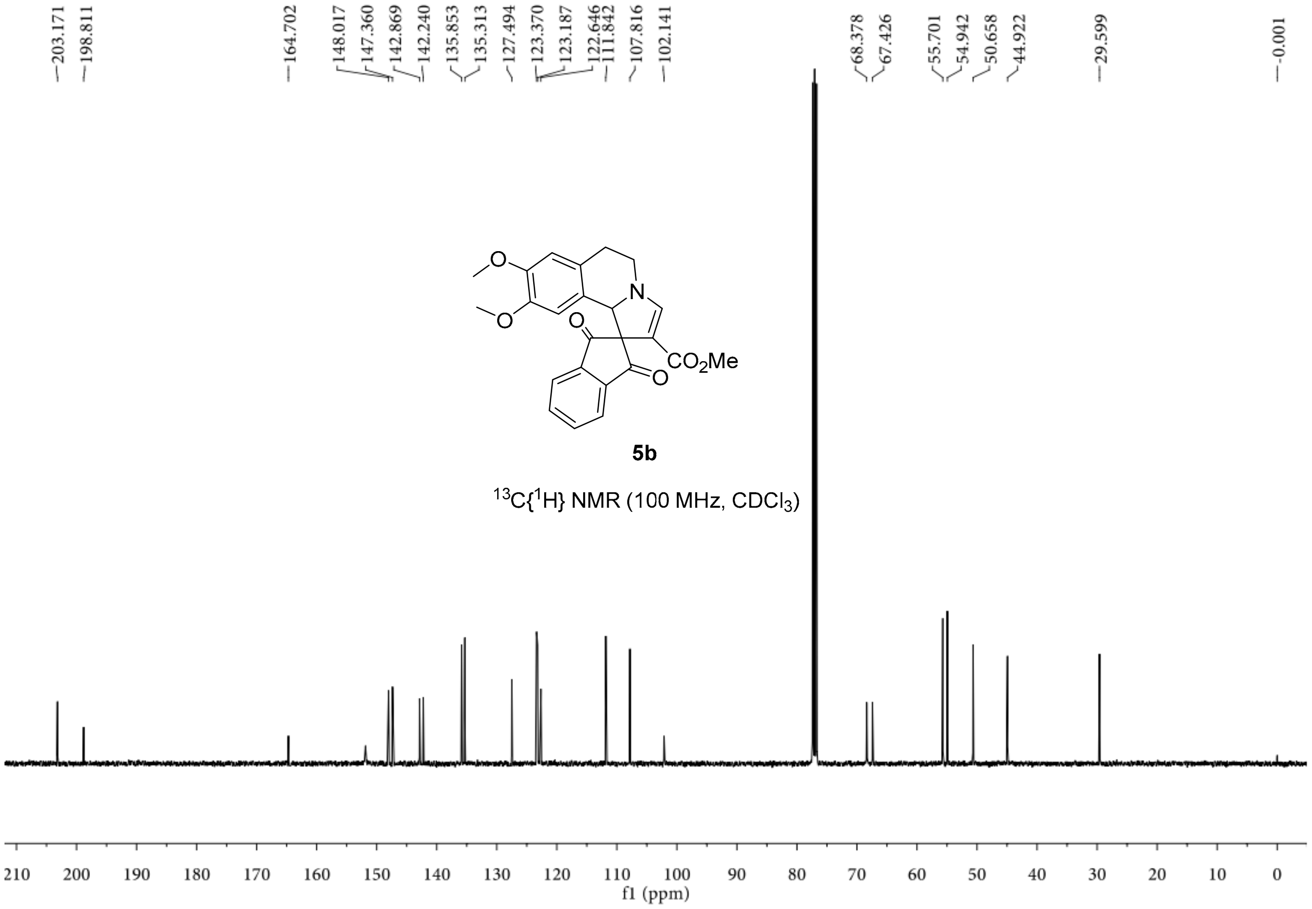
203.171 198.811 164.702 148.017 147.360 142.869 142.240 135.853 135.313 127.494 123.370 123.187 122.646 111.842 107.816 102.141
68.378 67.426 55.701 54.942 50.658 44.922 29.599 -0.001
CO2Me
5b
13C{1H} NMR (100 MHz, CDCl3)
f1 (ppm)

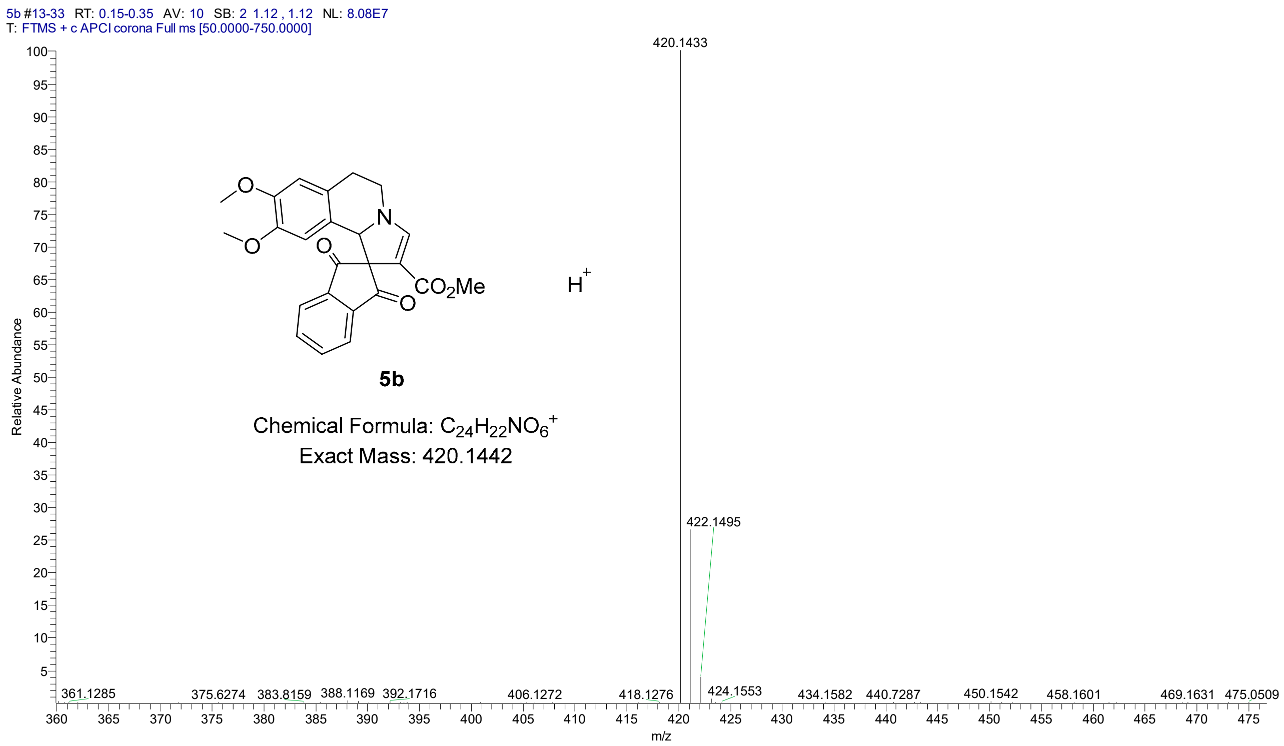

[M + H]+ calcd for $C_{24}H_{22}NO_6$ 420.1442, found 420.1433.

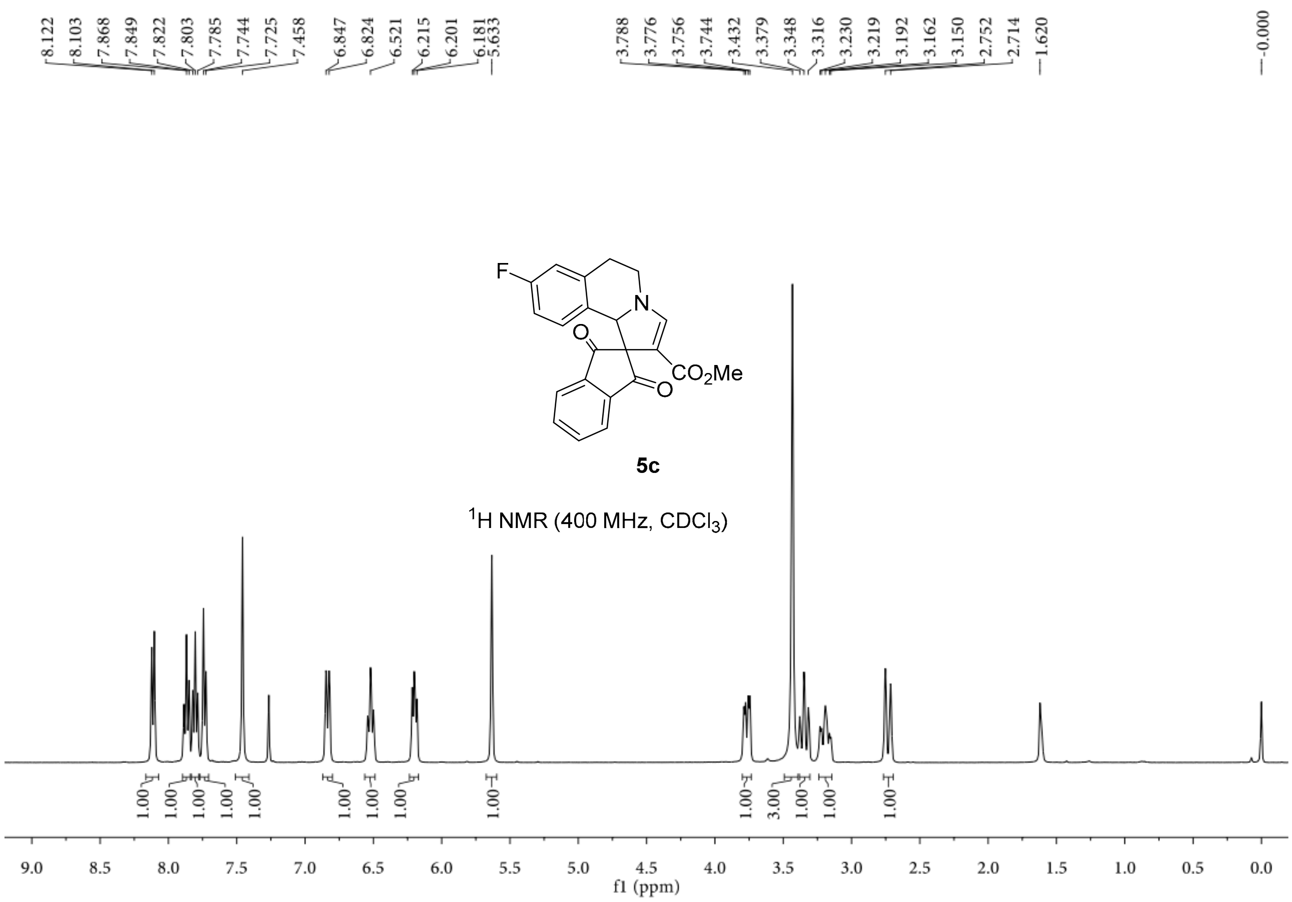
8.122
8.103
7.868
7.849
7.822
7.803
7.785
7.744
7.725
7.458
6.847
6.824
6.521
6.215
6.201
6.181
5.633
3.788
3.776
3.756
3.744
3.432
3.379
3.348
3.316
3.230
3.219
3.192
3.162
3.150
2.752
2.714
1.620
-0.000
F
N
O
CO2Me
O
5c
1H NMR (400 MHz, CDCl3)
1.00
1.00
1.00
1.00
1.00
1.00
1.00
1.00
1.00
1.00
3.00
1.00
1.00
1.00
9.0
8.5
8.0
7.5
7.0
6.5
6.0
5.5
5.0
4.5
4.0
3.5
3.0
2.5
2.0
1.5
1.0
0.5
0.0
f1 (ppm)

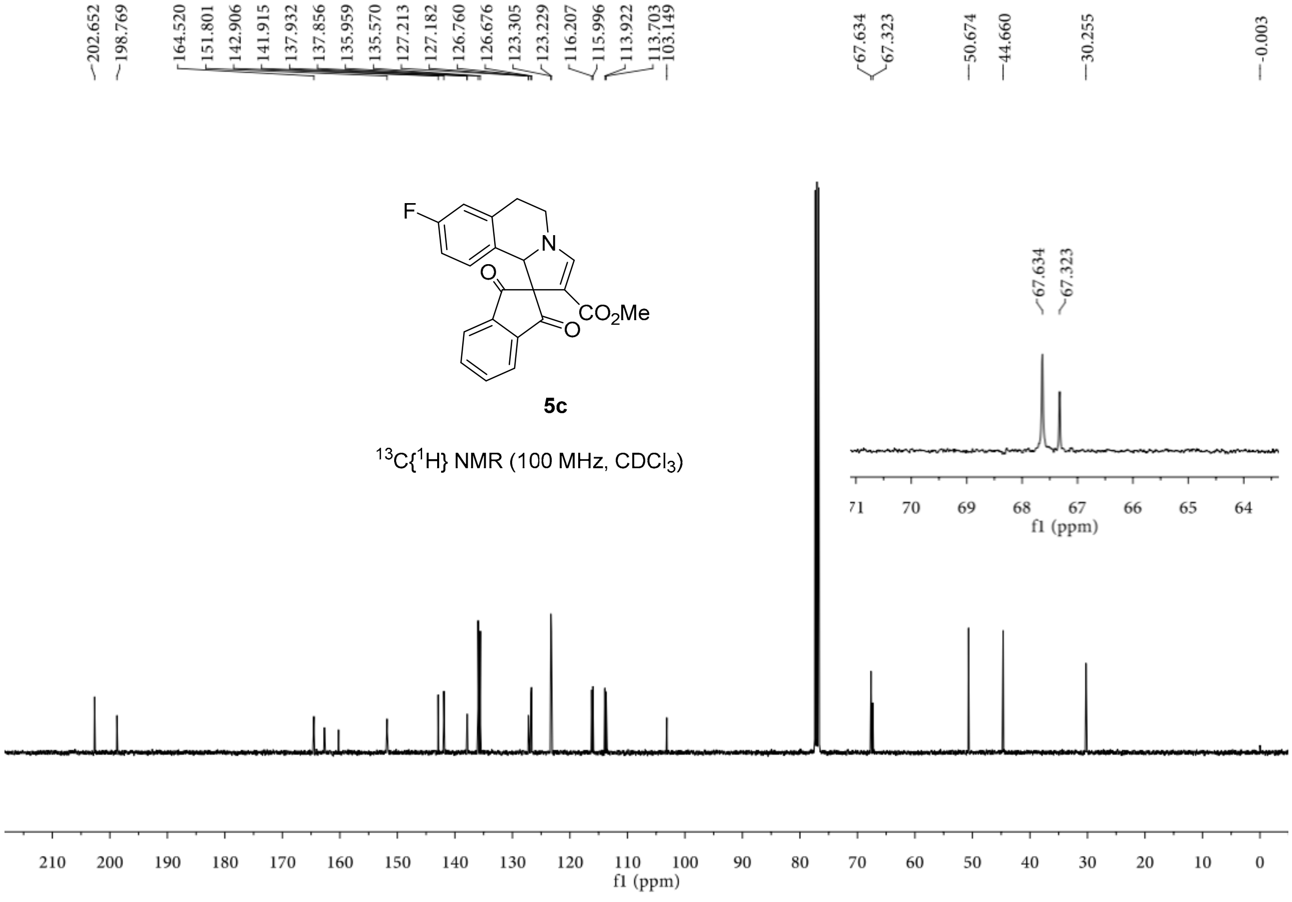
202.652
198.769
164.520
151.801
142.906
141.915
137.932
137.856
135.959
135.570
127.213
127.182
126.760
126.676
123.305
123.229
116.207
115.996
113.922
113.703
103.149
67.634
67.323
50.674
44.660
30.255
-0.003
F
N
O
CO2Me
O
5c
13C{1H} NMR (100 MHz, CDCl3)
67.634
67.323
71
70
69
68
67
66
65
64
f1 (ppm)
210
200
190
180
170
160
150
140
130
120
110
100
90
80
70
60
50
40
30
20
10
0
f1 (ppm)

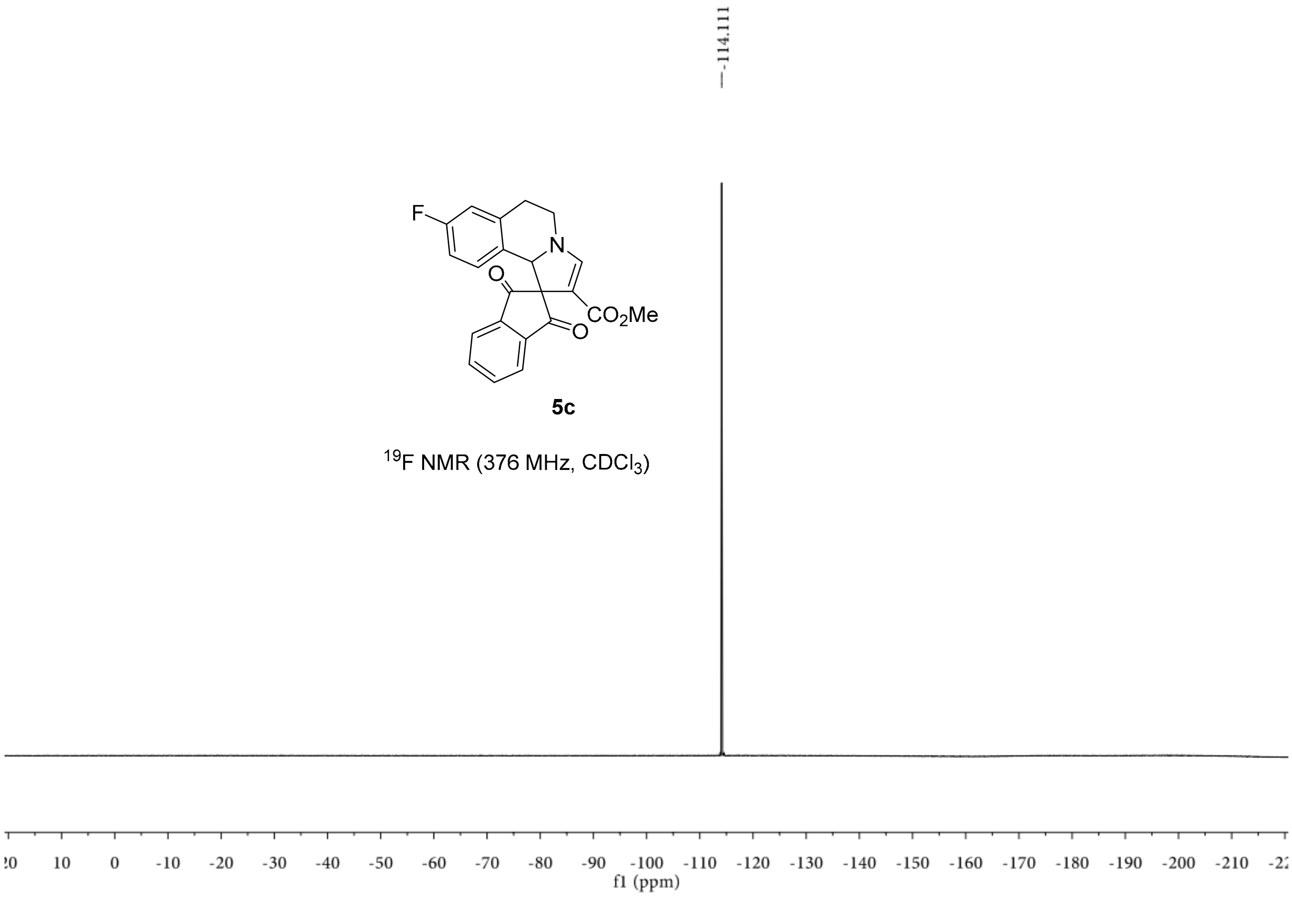

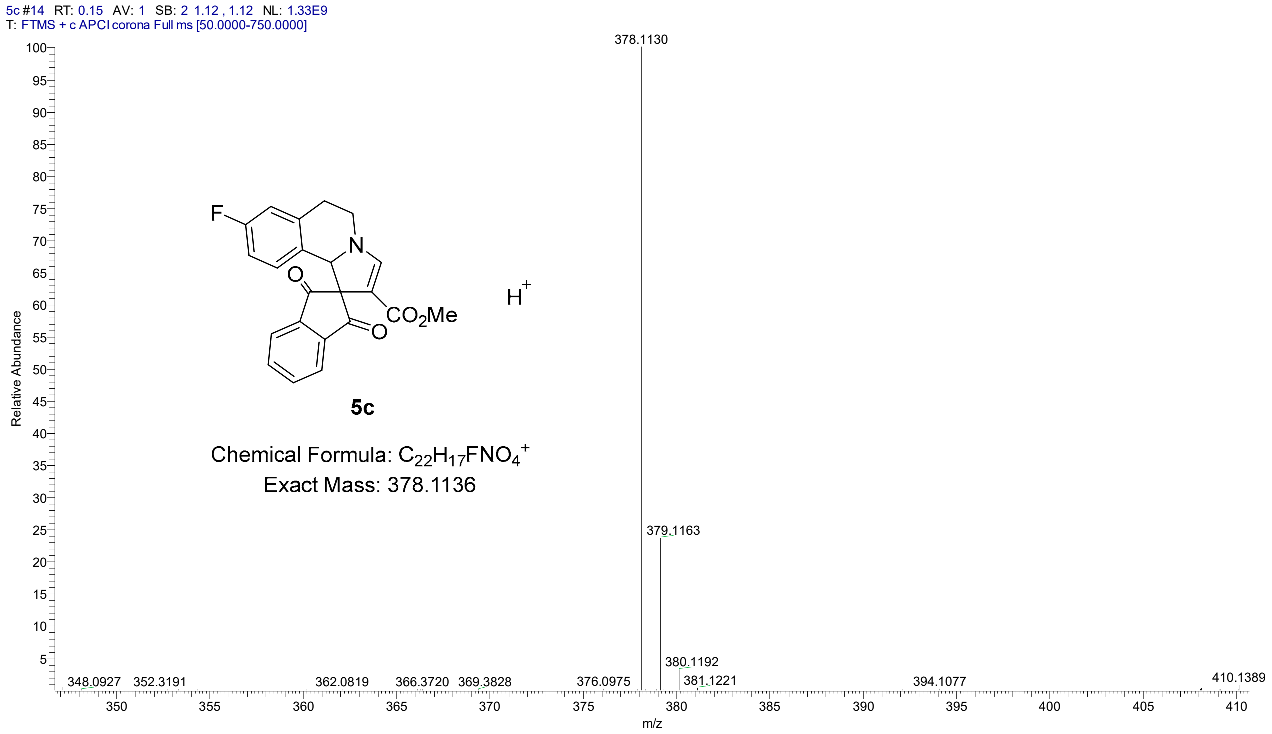

$[M + H]^+$ calcd for $C_{22}H_{17}FNO_4$ 378.1136, found 378.1130.

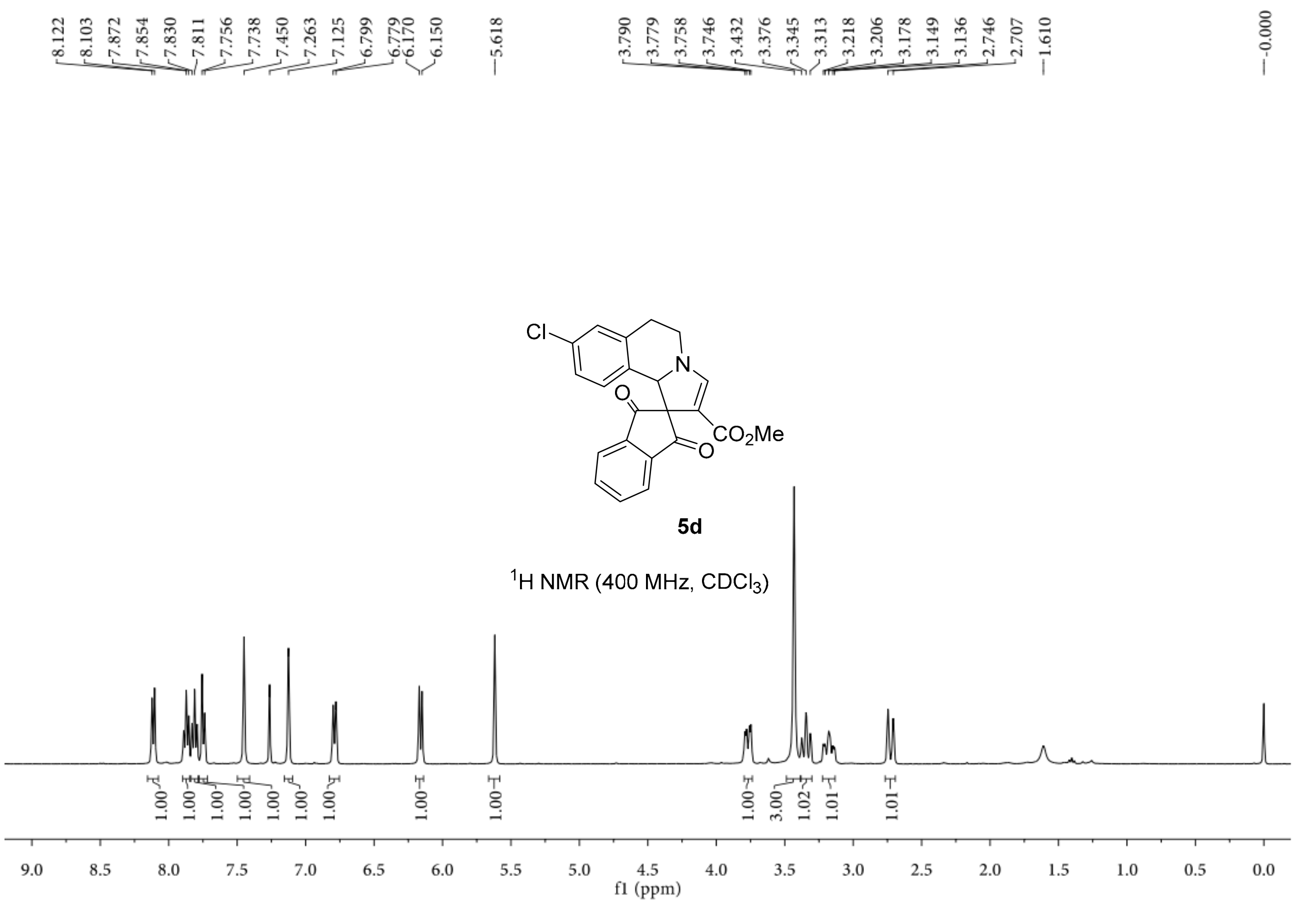
8.122 8.103 7.872 7.854 7.830 7.811 7.756 7.738 7.450 7.263 7.125 6.799 6.779 6.170 6.150
5.618
3.790 3.779 3.758 3.746 3.432 3.376 3.345 3.313 3.218 3.206 3.178 3.149 3.136 2.746 2.707
1.610
-0.000
Cl
N
O
O
CO2Me
5d
1H NMR (400 MHz, CDCl3)
1.00 1.00 1.00 1.00 1.00 1.00 1.00 1.00 1.00 1.00 3.00 1.02 1.01 1.01
f1 (ppm)

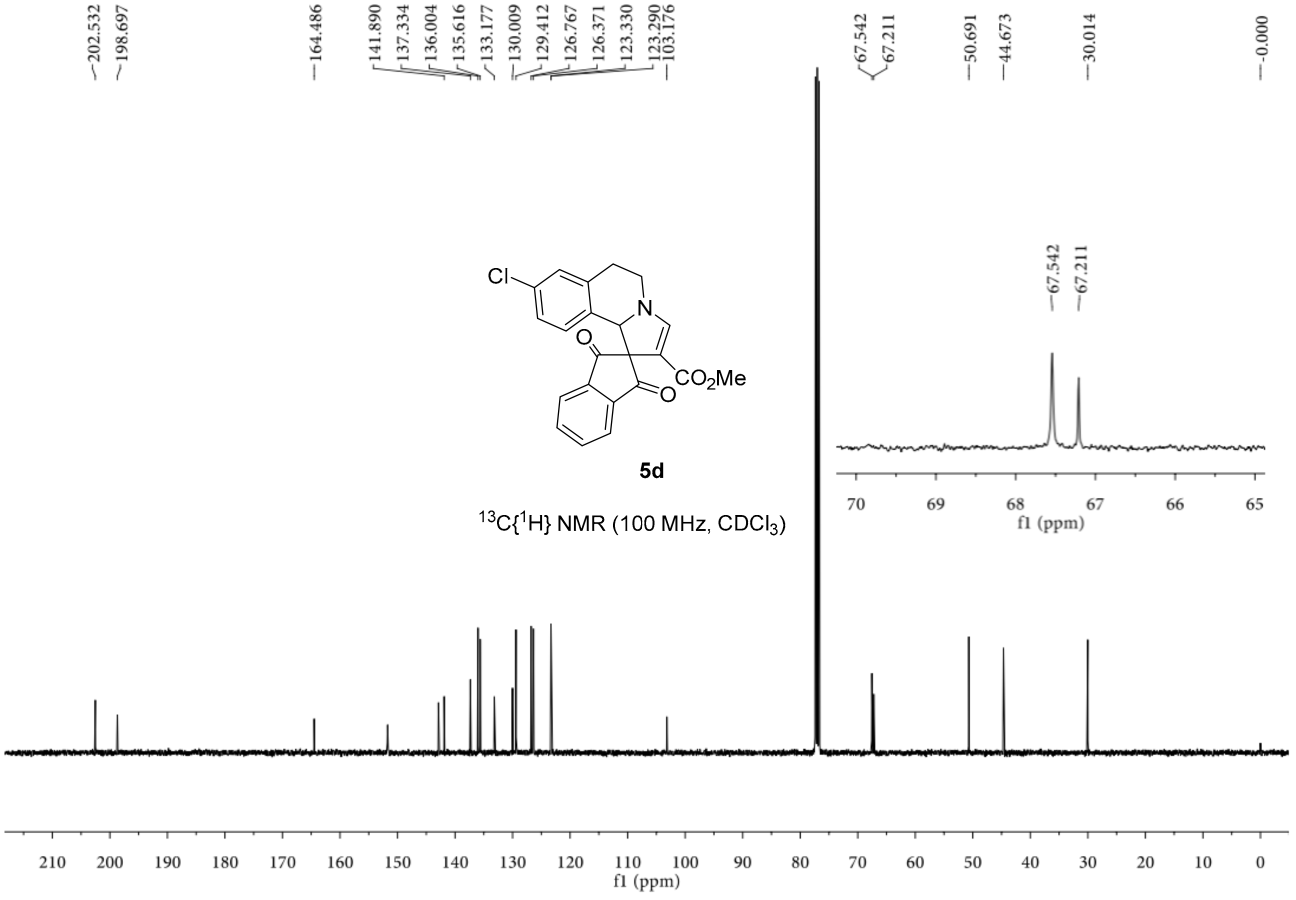
202.532 198.697
164.486
141.890 137.334 136.004 135.616 133.177 130.009 129.412 126.767 126.371 123.330 123.290 103.176
67.542 67.211
50.691 44.673
30.014
-0.000
Cl
N
O
O
CO2Me
5d
13C{1H} NMR (100 MHz, CDCl3)
f1 (ppm)

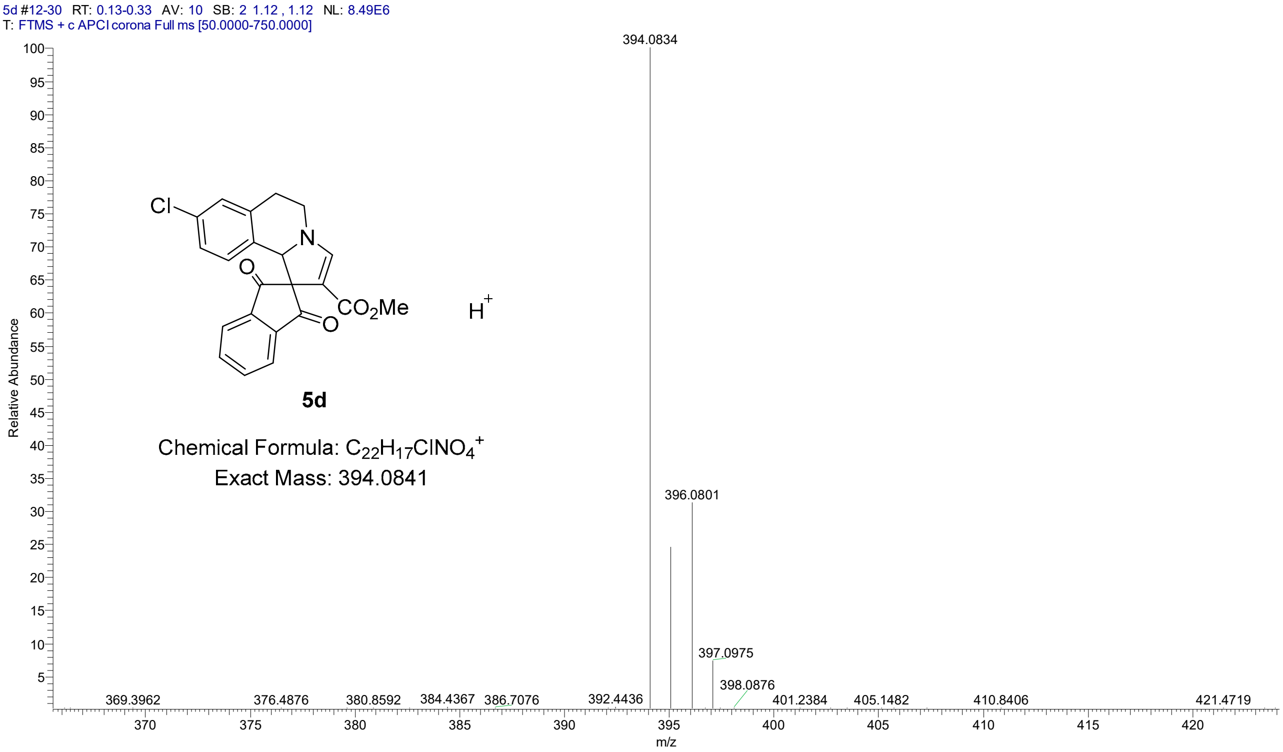

$[M + H]^+$ calcd for $C_{22}H_{17}ClNO_4$ 394.0841, found 394.0834.

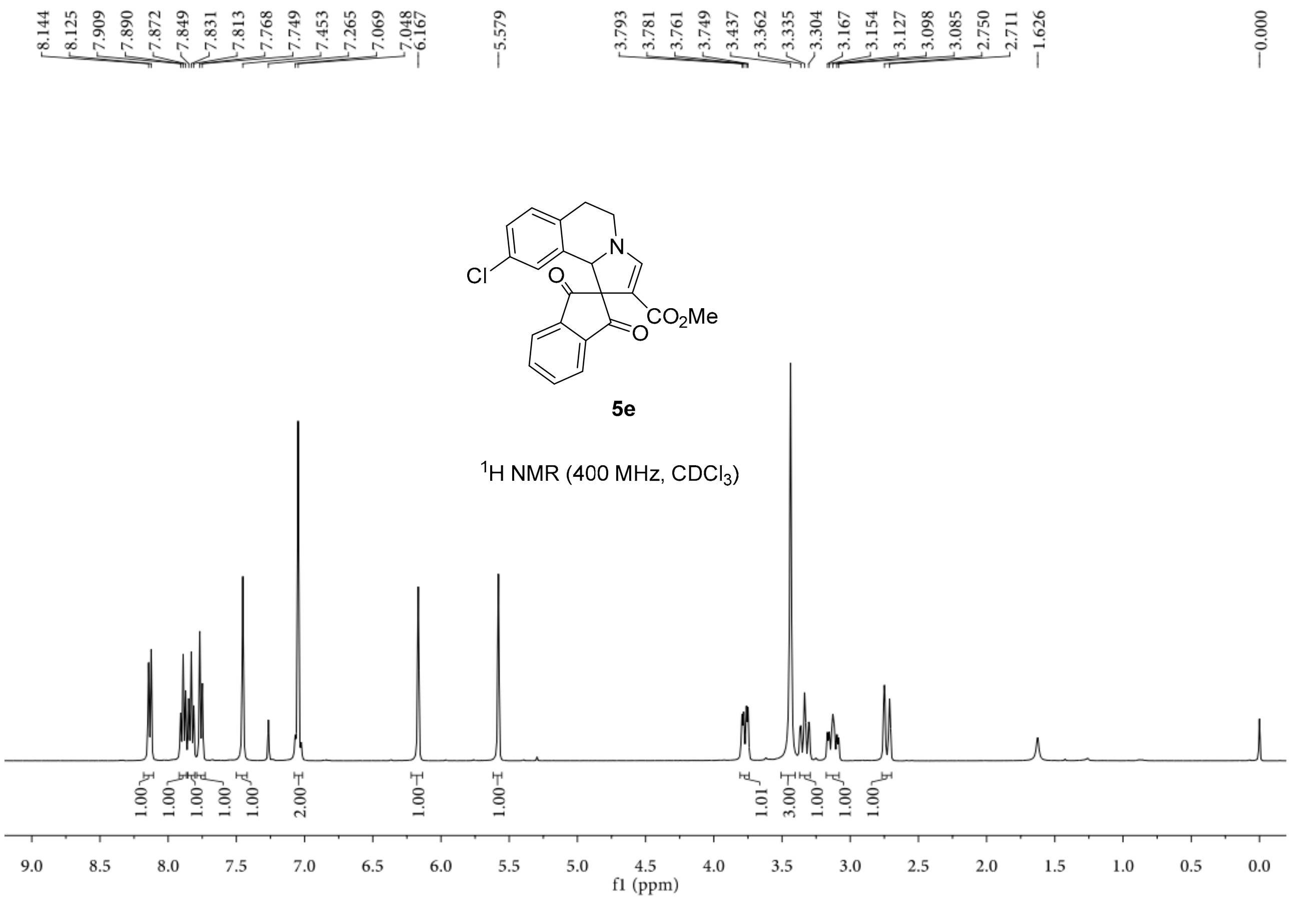
8.144
8.125
7.909
7.890
7.872
7.849
7.831
7.813
7.768
7.749
7.453
7.265
7.069
7.048
6.167
5.579
3.793
3.781
3.761
3.749
3.437
3.362
3.335
3.304
3.167
3.154
3.127
3.098
3.085
2.750
2.711
1.626
0.000
Cl
N
O
O
CO2Me
5e
1H NMR (400 MHz, CDCl3)
1.00
1.00
1.00
1.00
1.00
2.00
1.00
1.00
1.01
3.00
1.00
1.00
1.00
f1 (ppm)

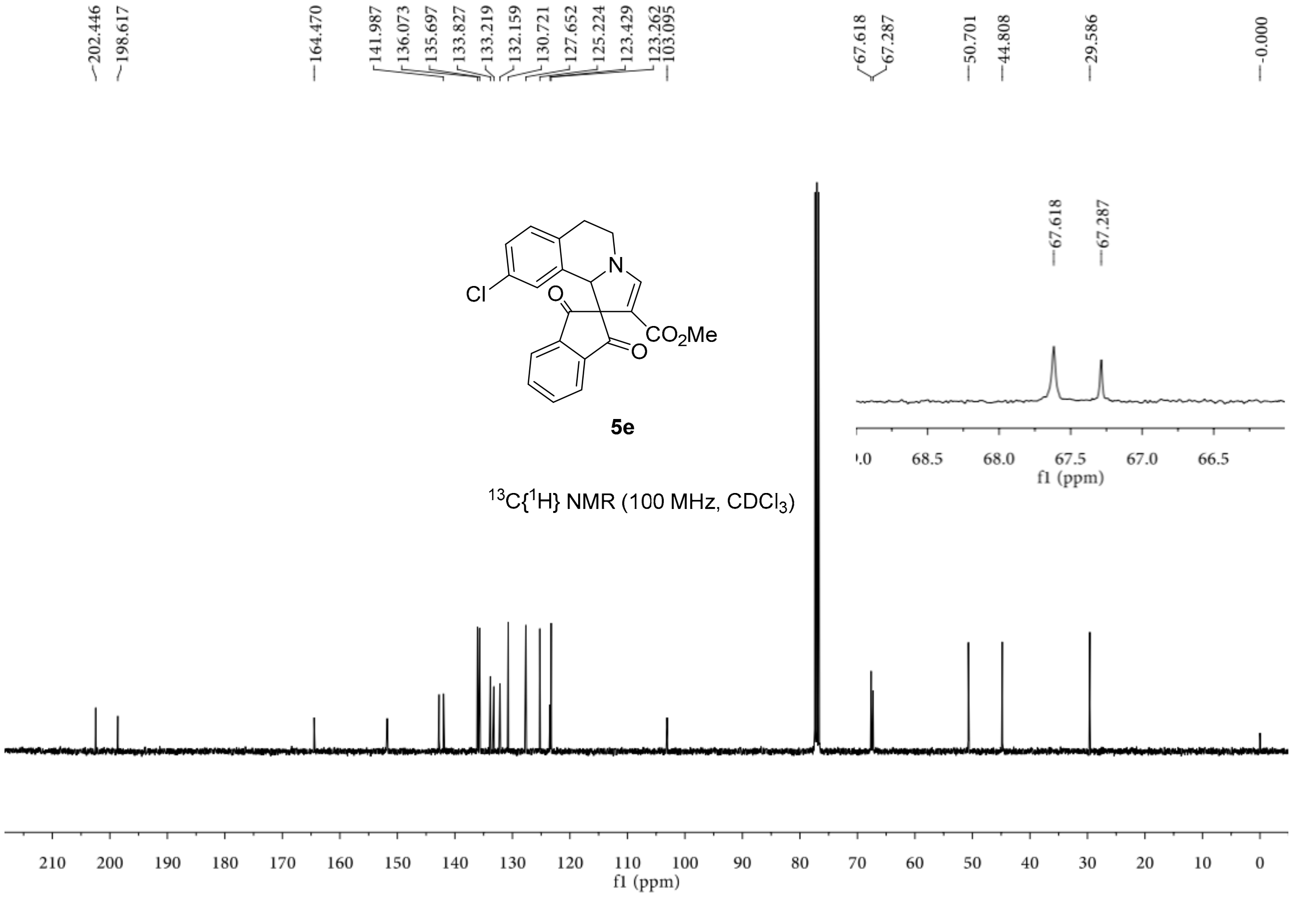
202.446
198.617
164.470
141.987
136.073
135.697
133.827
133.219
132.159
130.721
127.652
125.224
123.429
123.262
103.095
67.618
67.287
50.701
44.808
29.586
-0.000
Cl
N
O
O
CO2Me
5e
13C{1H} NMR (100 MHz, CDCl3)
f1 (ppm)

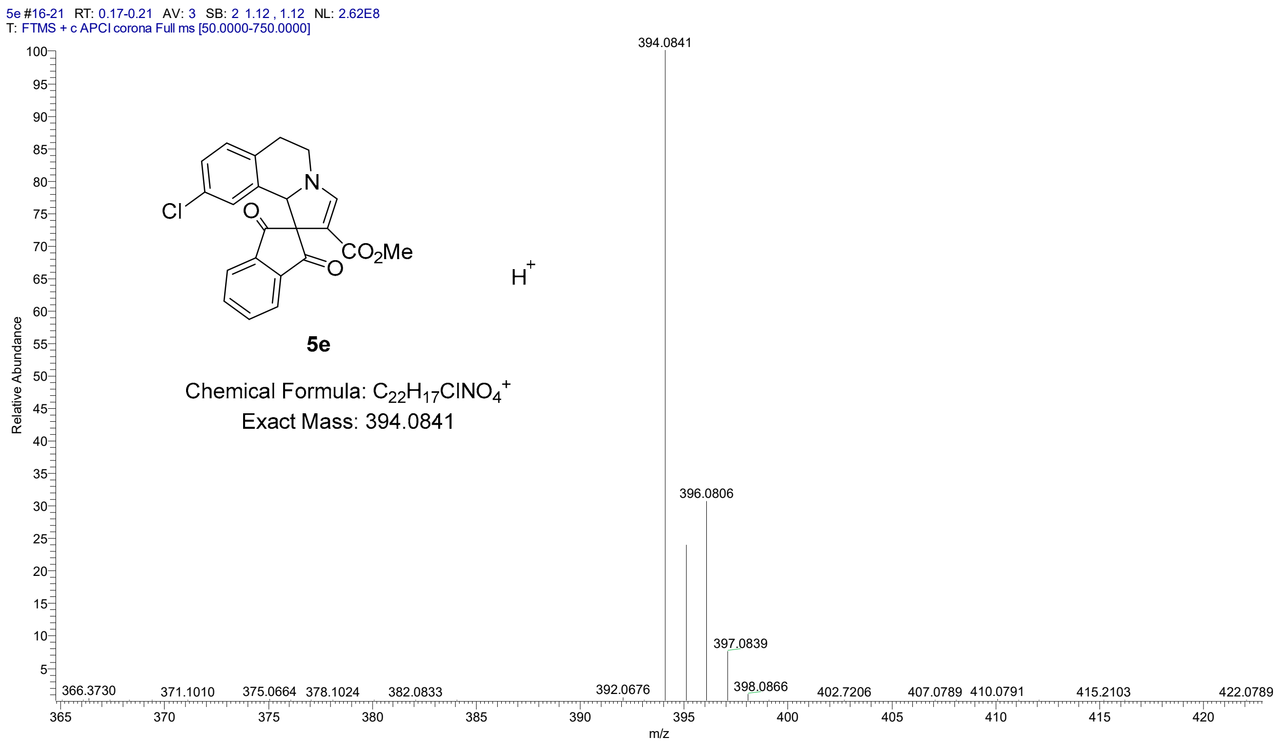

$[M + H]^+$ calcd for $C_{22}H_{17}ClNO_4$ 394.0841, found 394.0841.

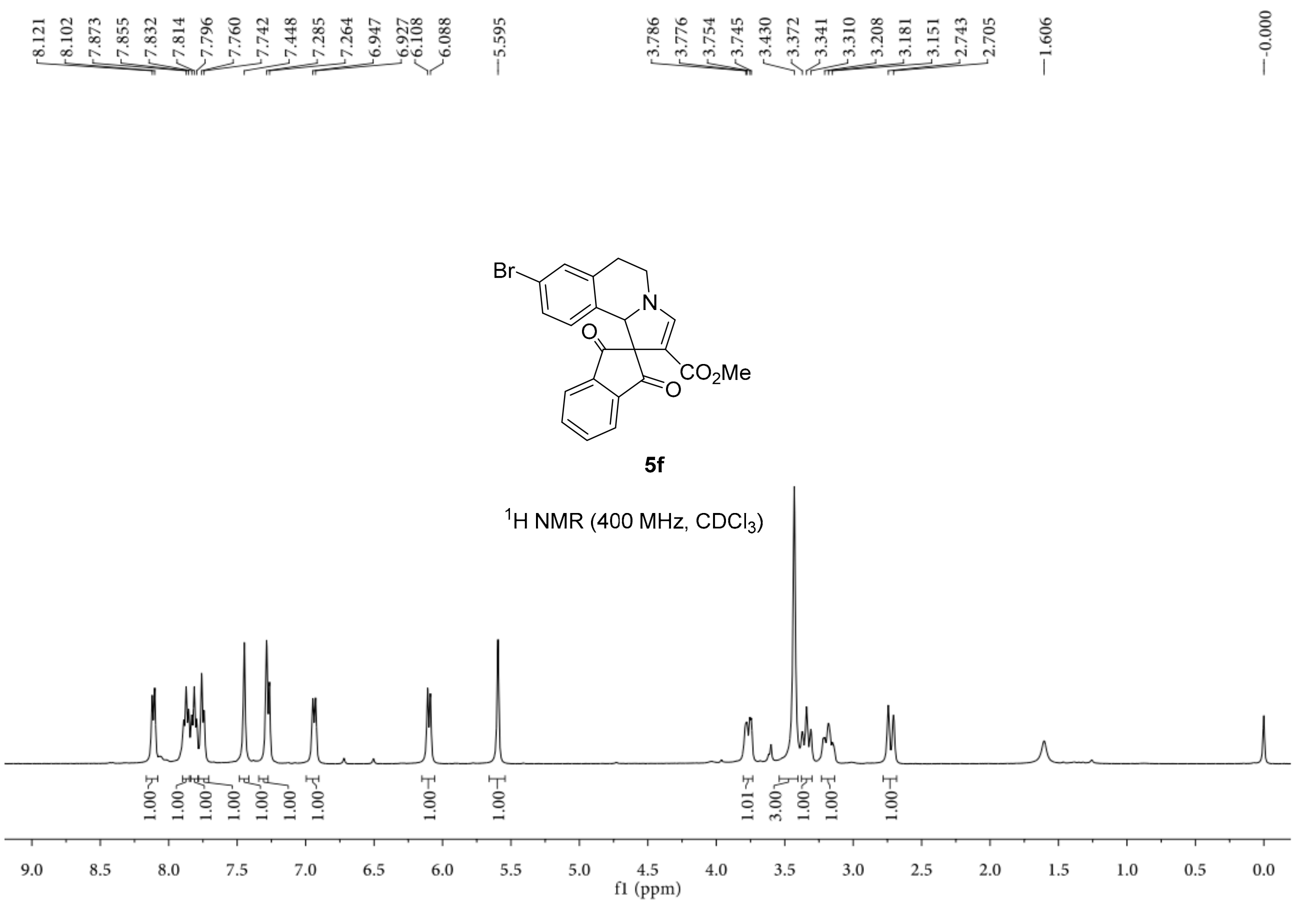
8.121
8.102
7.873
7.855
7.832
7.814
7.796
7.760
7.742
7.448
7.285
7.264
6.947
6.927
6.108
6.088
5.595
3.786
3.776
3.754
3.745
3.430
3.372
3.341
3.310
3.208
3.181
3.151
2.743
2.705
1.606
-0.000
Br
N
O
CO2Me
O
5f
1H NMR (400 MHz, CDCl3)
f1 (ppm)

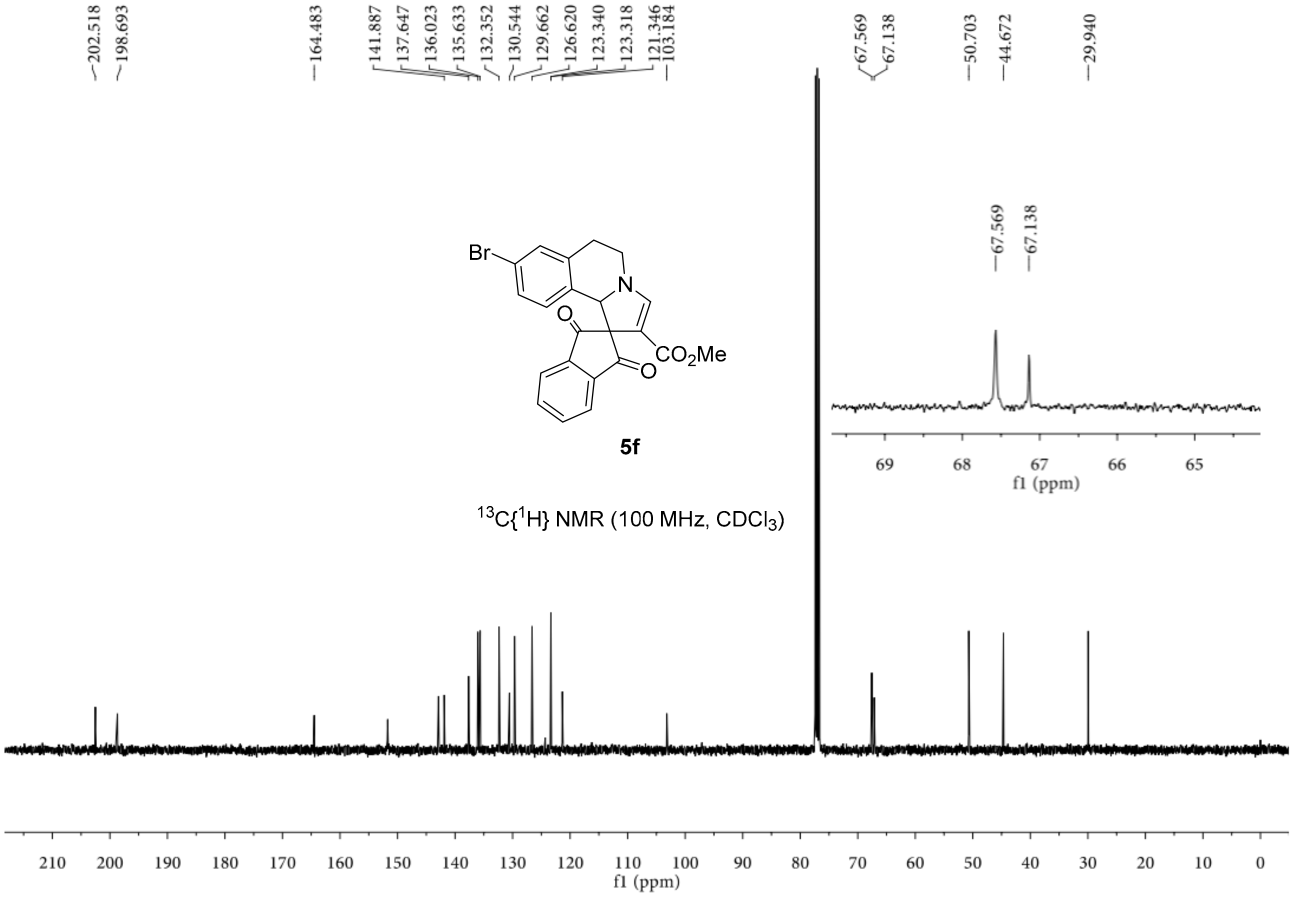
202.518
198.693
164.483
141.887
137.647
136.023
135.633
132.352
130.544
129.662
126.620
123.340
123.318
121.346
103.184
67.569
67.138
50.703
44.672
29.940
Br
N
O
CO2Me
O
5f
13C{1H} NMR (100 MHz, CDCl3)
f1 (ppm)

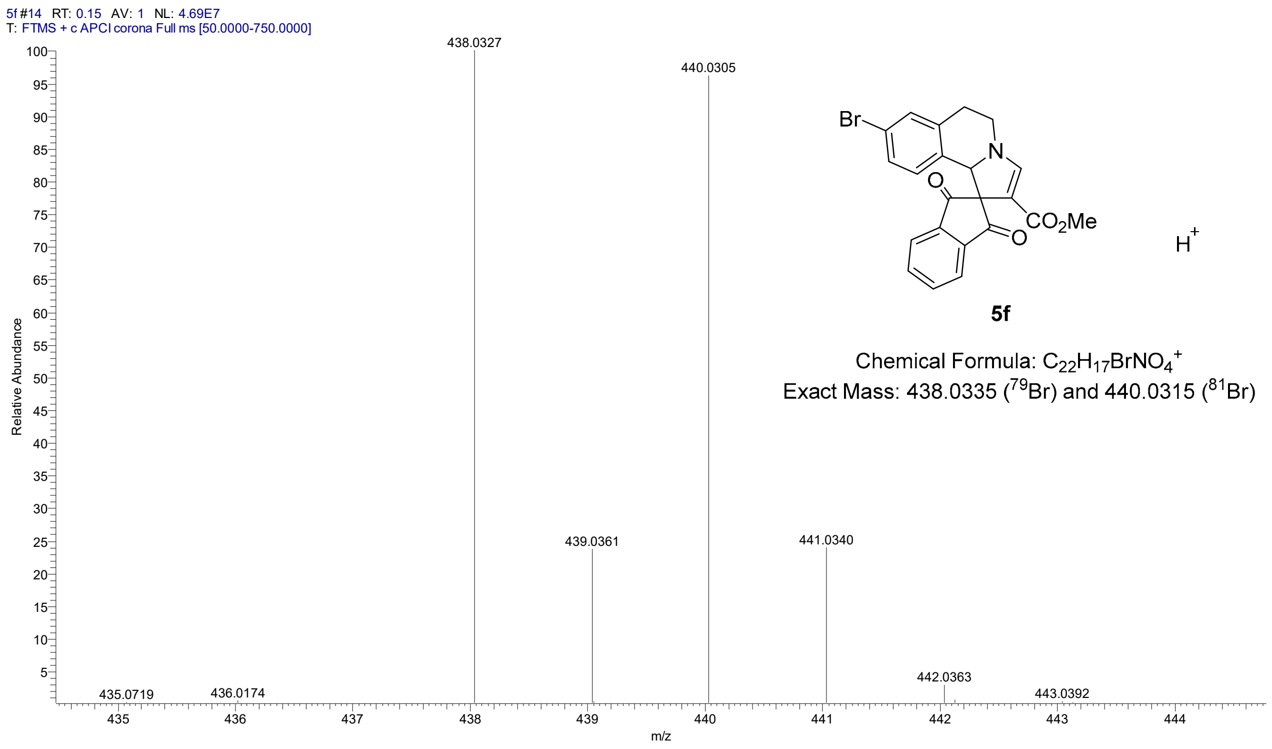

$[M + H]^+$ calcd for $C_{22}H_{17}BrNO_4$ 438.0335 (^{79}Br) and 440.0315 (^{81}Br), found 438.0327, 440.0305.

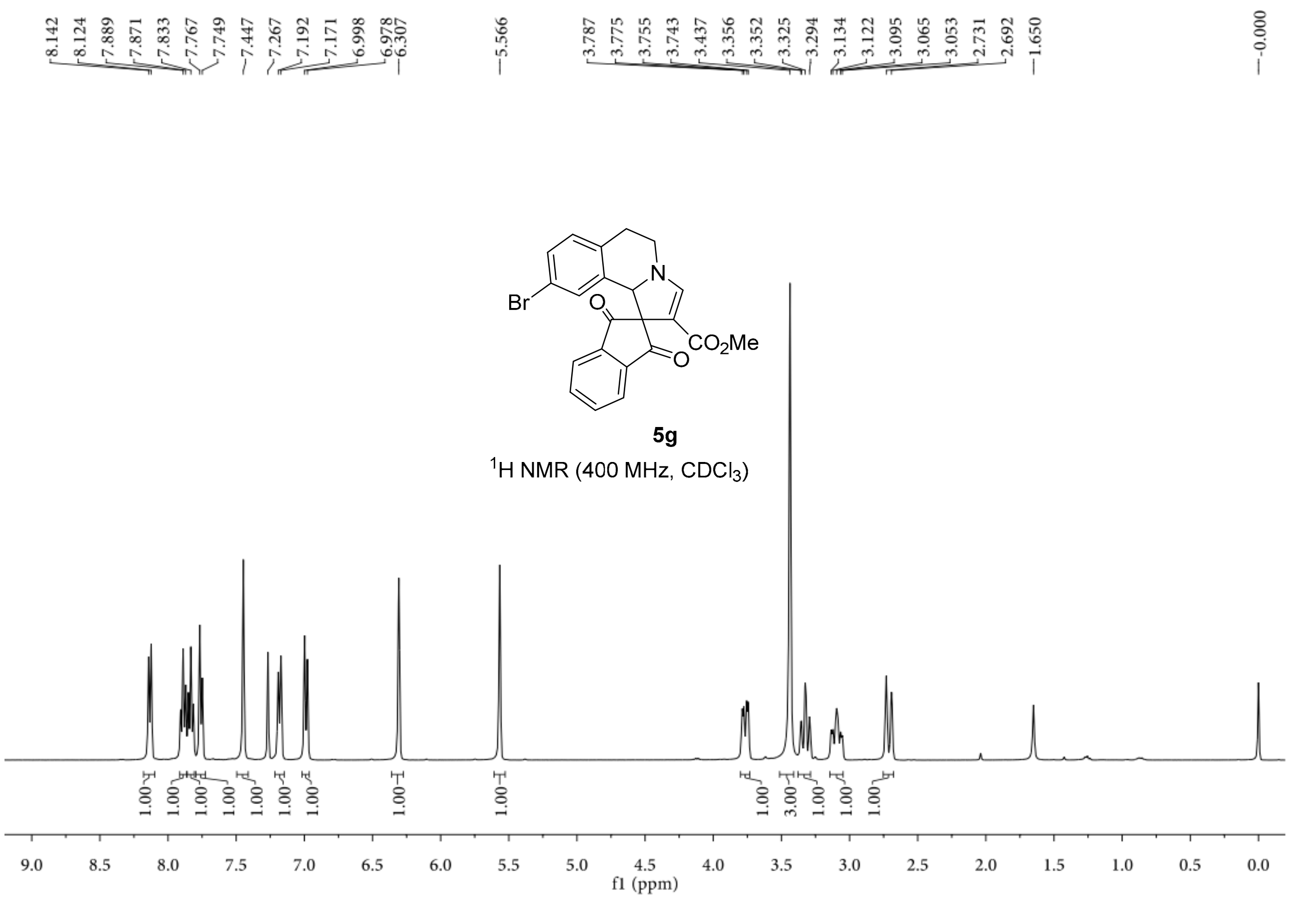
8.142
8.124
7.889
7.871
7.833
7.767
7.749
7.447
7.267
7.192
7.171
6.998
6.978
6.307
5.566
3.787
3.775
3.755
3.743
3.437
3.356
3.352
3.325
3.294
3.134
3.122
3.095
3.065
3.053
2.731
2.692
1.650
-0.000
Br
N
O
CO2Me
5g
1H NMR (400 MHz, CDCl3)
1.00
1.00
1.00
1.00
1.00
1.00
1.00
1.00
1.00
1.00
3.00
1.00
1.00
1.00
9.0 8.5 8.0 7.5 7.0 6.5 6.0 5.5 5.0 4.5 4.0 3.5 3.0 2.5 2.0 1.5 1.0 0.5 0.0
f1 (ppm)

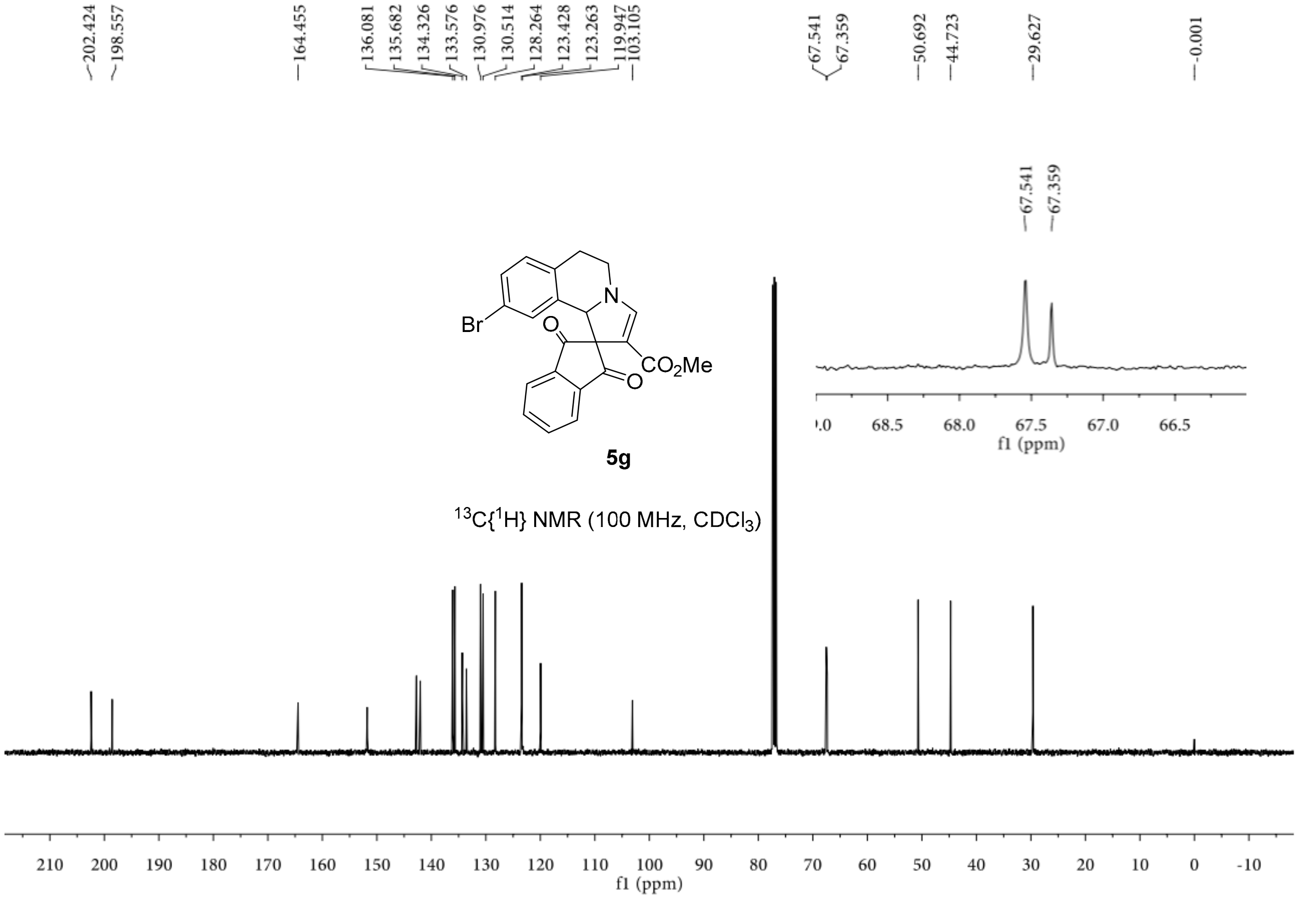
202.424
198.557
164.455
136.081
135.682
134.326
133.576
130.976
130.514
128.264
123.428
123.263
119.947
103.105
67.541
67.359
50.692
44.723
29.627
-0.001
Br
N
O
CO2Me
5g
13C{1H} NMR (100 MHz, CDCl3)
68.5 68.0 67.5 67.0 66.5
f1 (ppm)
210 200 190 180 170 160 150 140 130 120 110 100 90 80 70 60 50 40 30 20 10 0 -10
f1 (ppm)

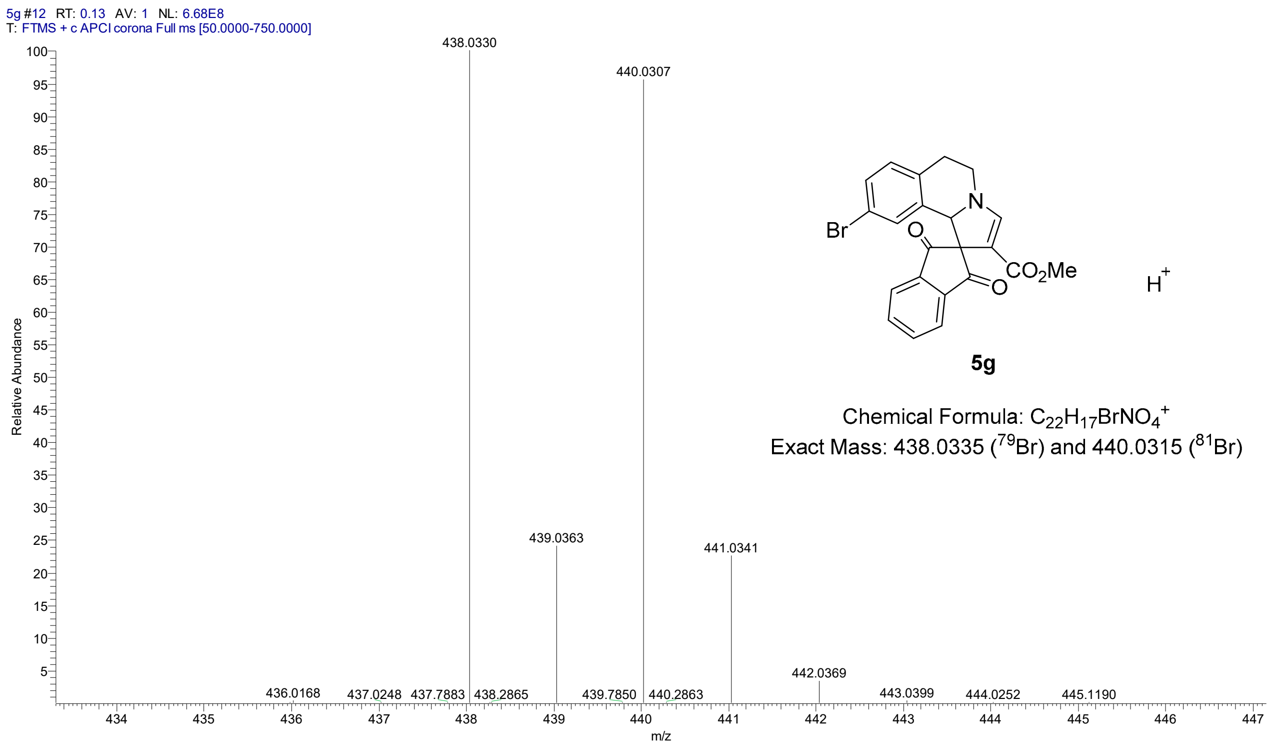

$[M + H]^+$ calcd for $C_{22}H_{17}BrNO_4$ 438.0335 (^{79}Br) and 440.0315 (^{81}Br), found 438.0330, 440.0307.

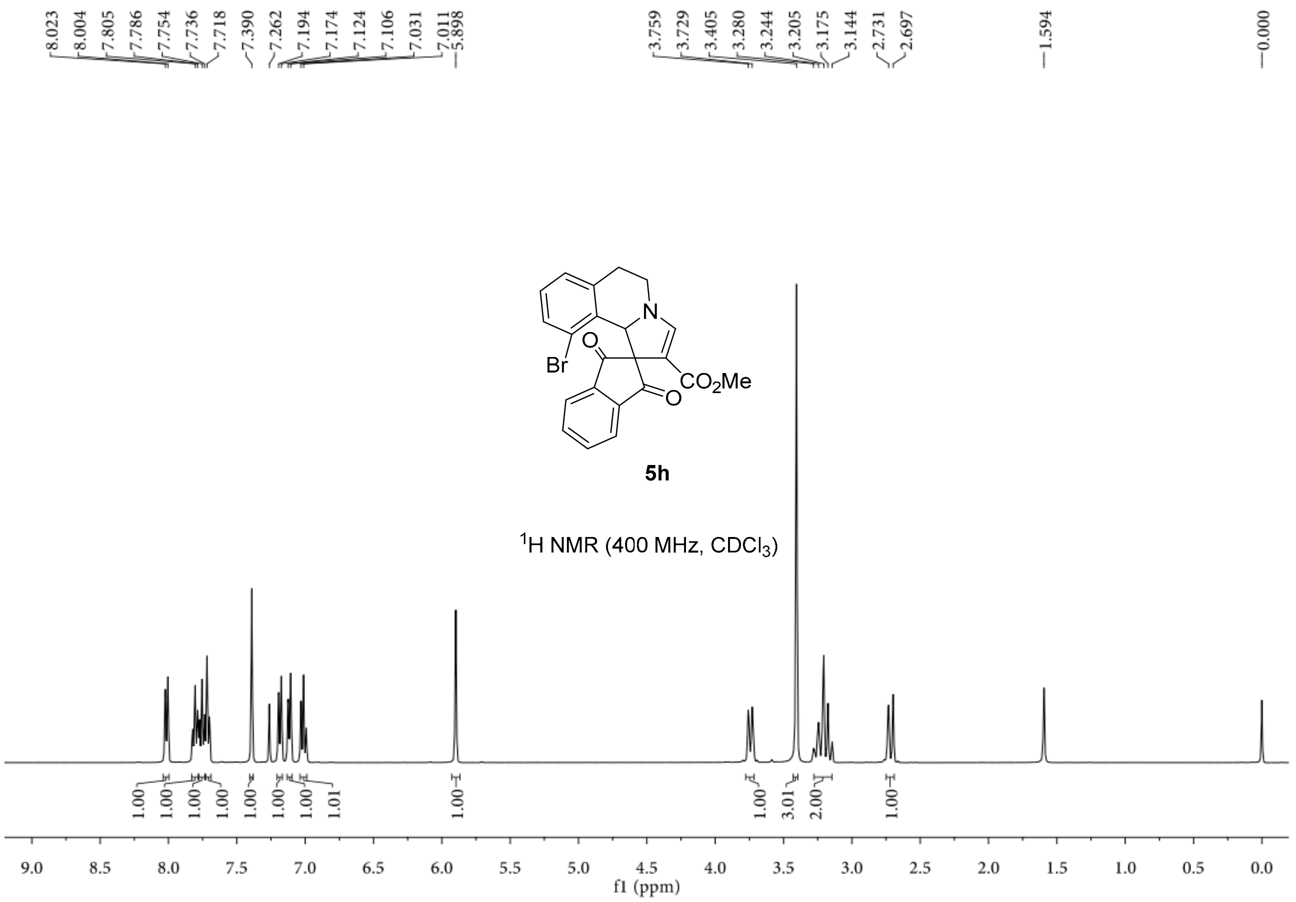
8.023
8.004
7.805
7.786
7.754
7.736
7.718
7.390
7.262
7.194
7.174
7.124
7.106
7.031
7.011
5.898
3.759
3.729
3.405
3.280
3.244
3.205
3.175
3.144
2.731
2.697
1.594
0.000
Br
N
O
O
CO_2Me
5h
^1H NMR (400 MHz, $CDCl_3$)
1.00
1.00
1.00
1.00
1.00
1.00
1.00
1.01
1.00
1.00
3.01
2.00
1.00
9.0
8.5
8.0
7.5
7.0
6.5
6.0
5.5
5.0
4.5
4.0
3.5
3.0
2.5
2.0
1.5
1.0
0.5
0.0
f1 (ppm)

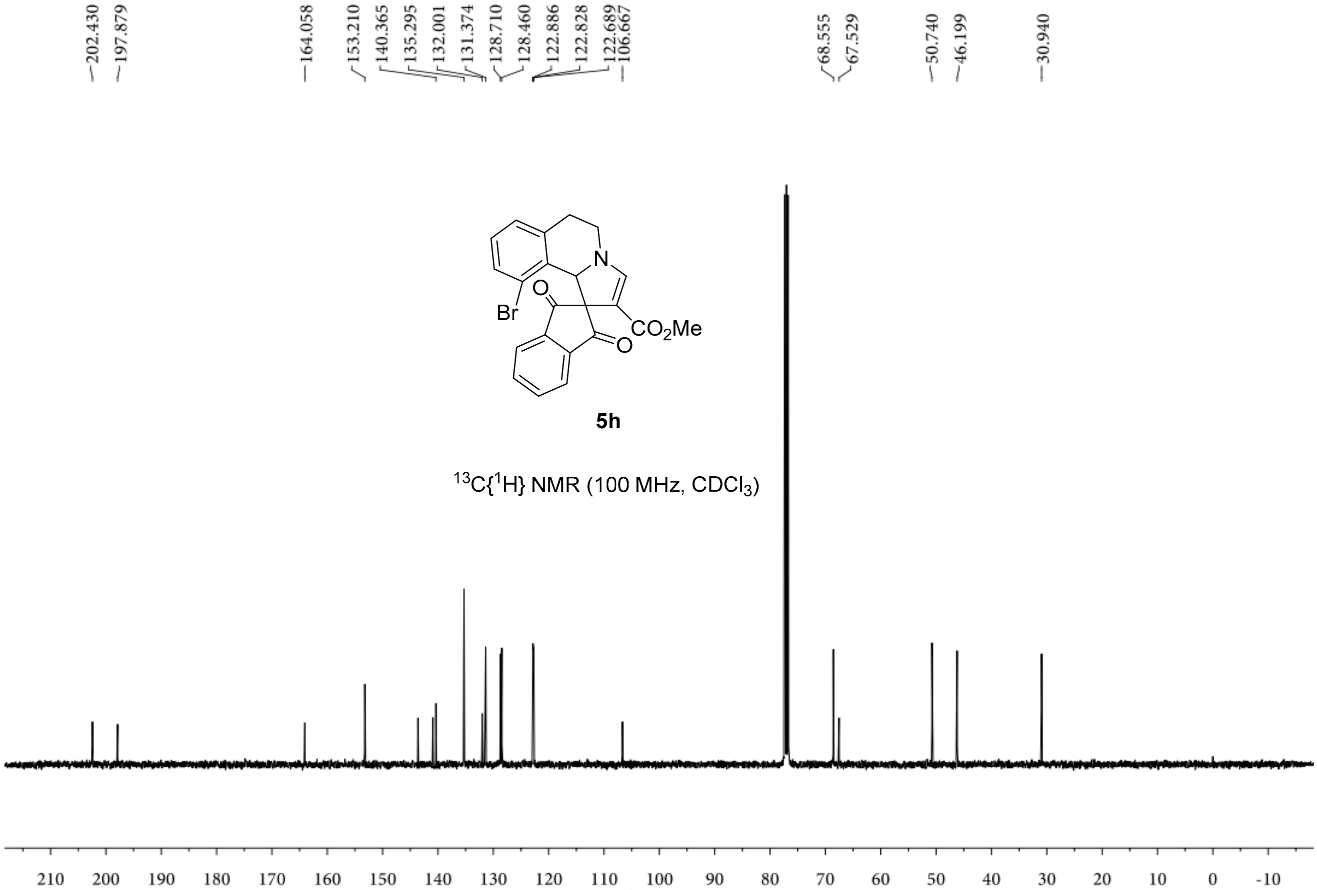
202.430
197.879
164.058
153.210
140.365
135.295
132.001
131.374
128.710
128.460
122.886
122.828
122.689
106.667
68.555
67.529
50.740
46.199
30.940
Br
N
O
O
CO_2Me
5h
$^{13}C\{^1H\}$ NMR (100 MHz, $CDCl_3$)
210
200
190
180
170
160
150
140
130
120
110
100
90
80
70
60
50
40
30
20
10
0
-10
f1 (ppm)

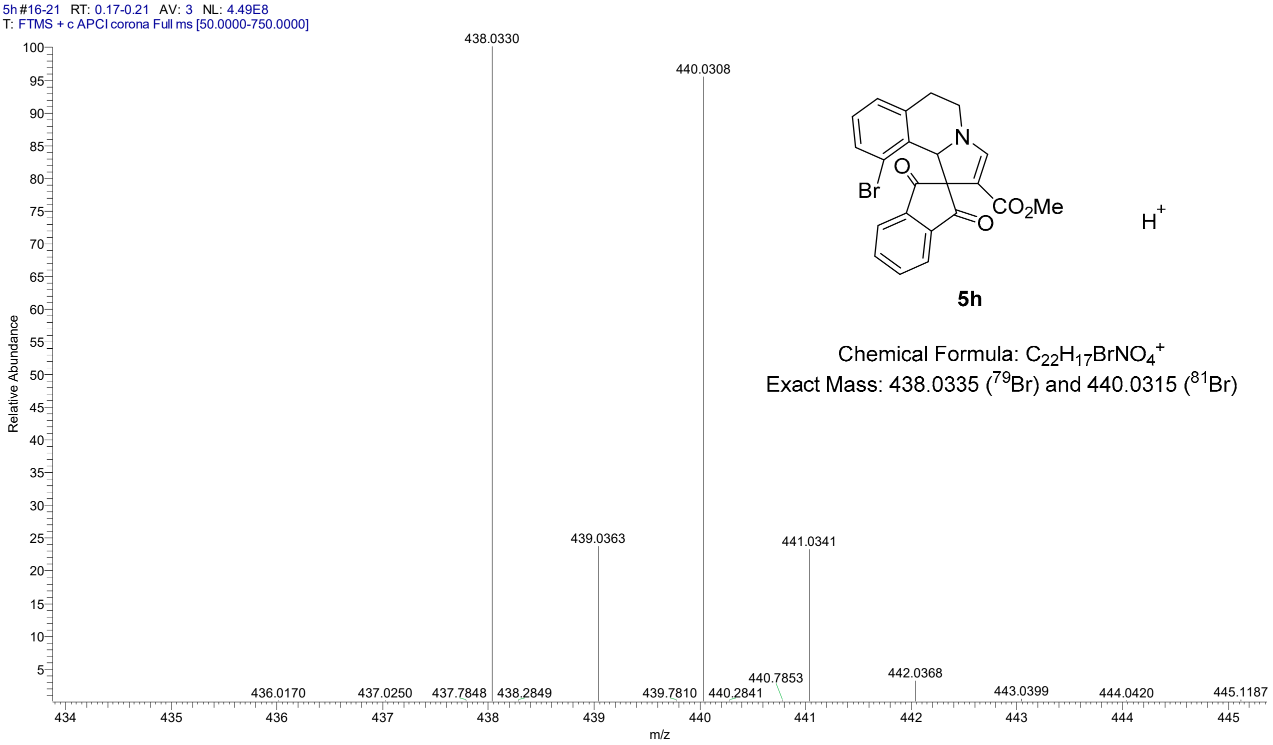

$[M + H]^+$ calcd for $C_{22}H_{17}BrNO_4$ 438.0335 (^{79}Br) and 440.0315 (^{81}Br), found 438.0330, 440.0308.

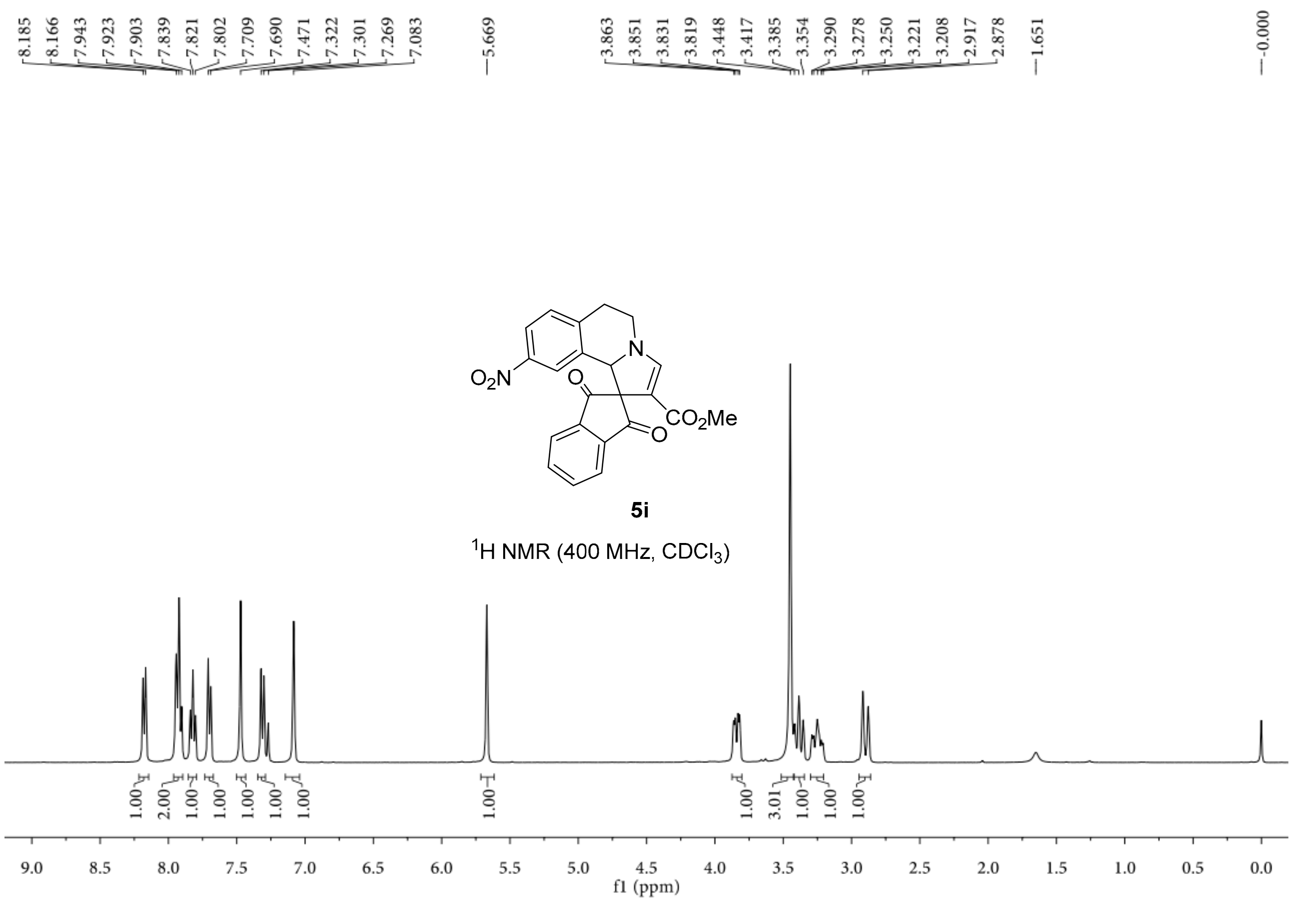
5i
^{1}H NMR (400 MHz, CDCl$_3$)

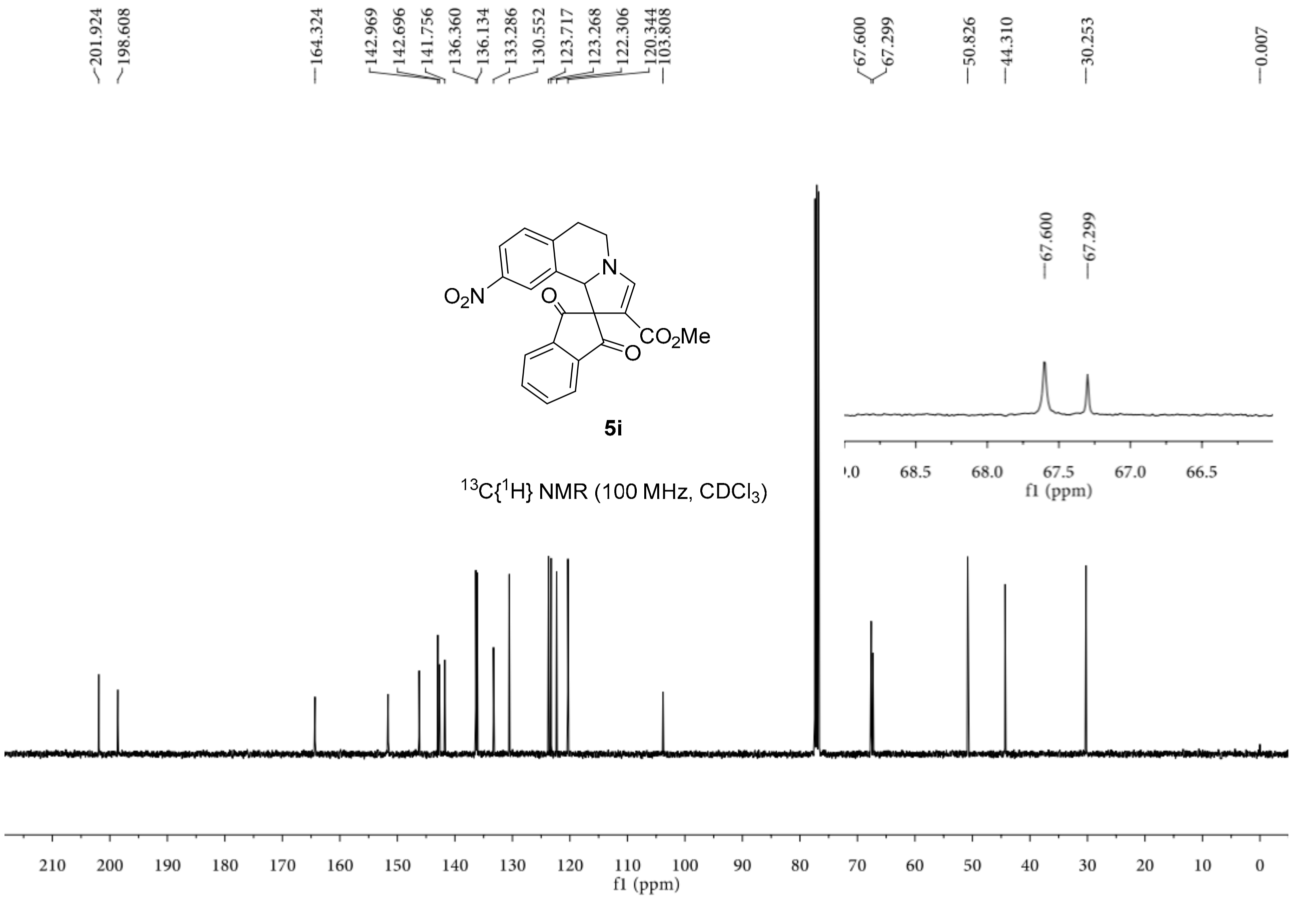
5i
^{13}C{^{1}H} NMR (100 MHz, CDCl$_3$)

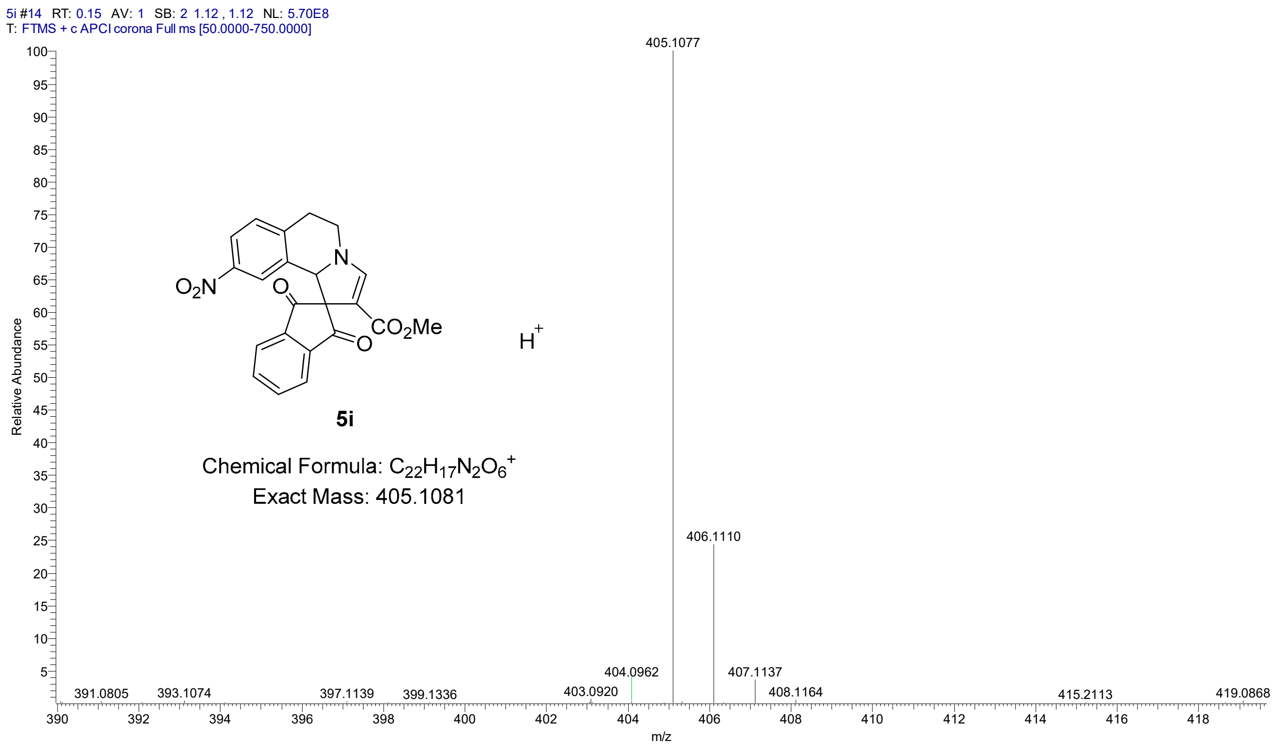

$[M + H]^+$ calcd for $C_{22}H_{17}N_2O_6$ 405.1081, found 405.1077.

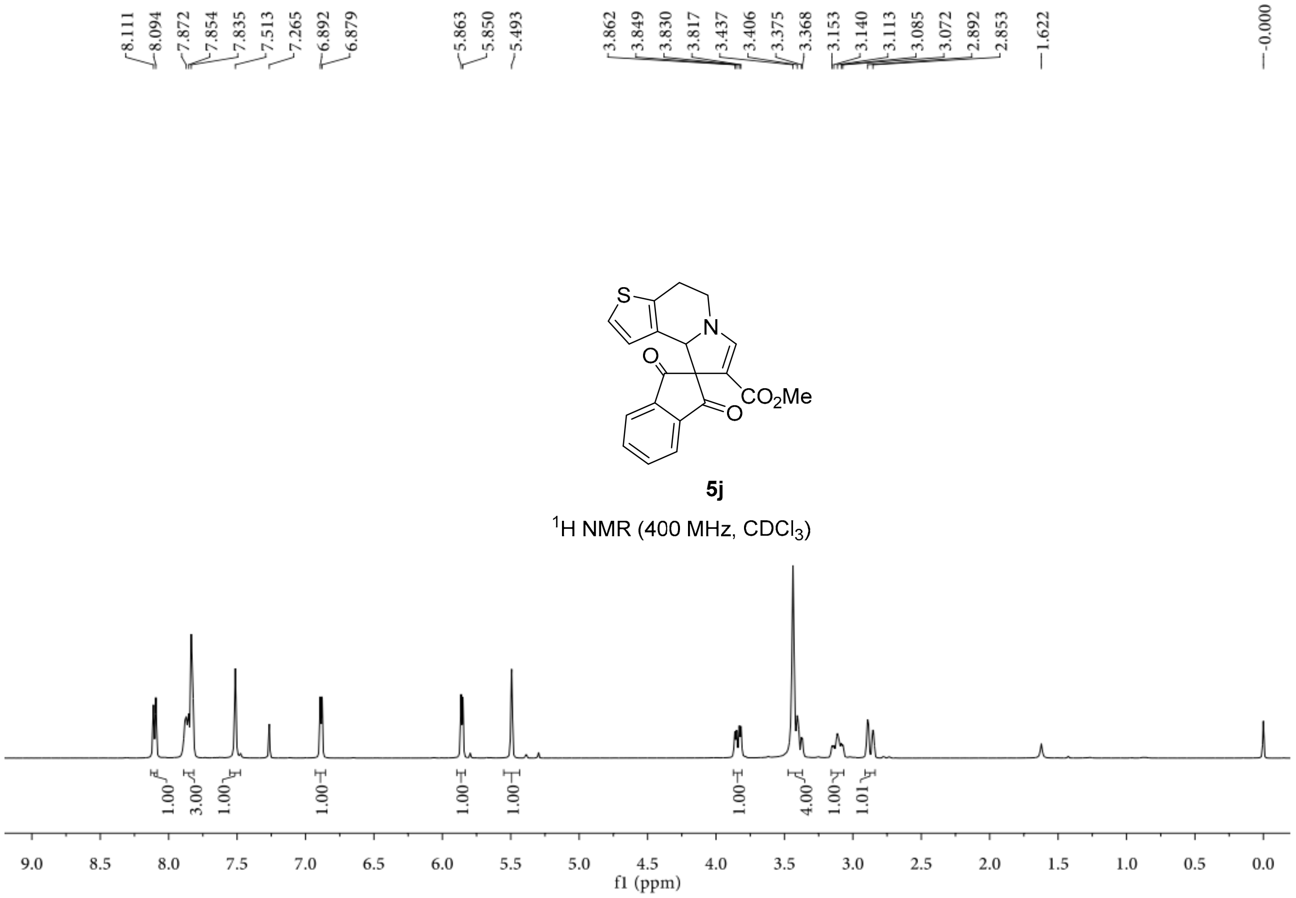

8.111
8.094
7.872
7.854
7.835
7.513
7.265
6.892
6.879
5.863
5.850
5.493
3.862
3.849
3.830
3.817
3.437
3.406
3.375
3.368
3.153
3.140
3.113
3.085
3.072
2.892
2.853
1.622
-0.000
S
N
O
O
CO2Me
5j
1H NMR (400 MHz, CDCl3)
1.00
3.00
1.00
1.00
1.00
1.00
1.00
4.00
1.00
1.01
f1 (ppm)

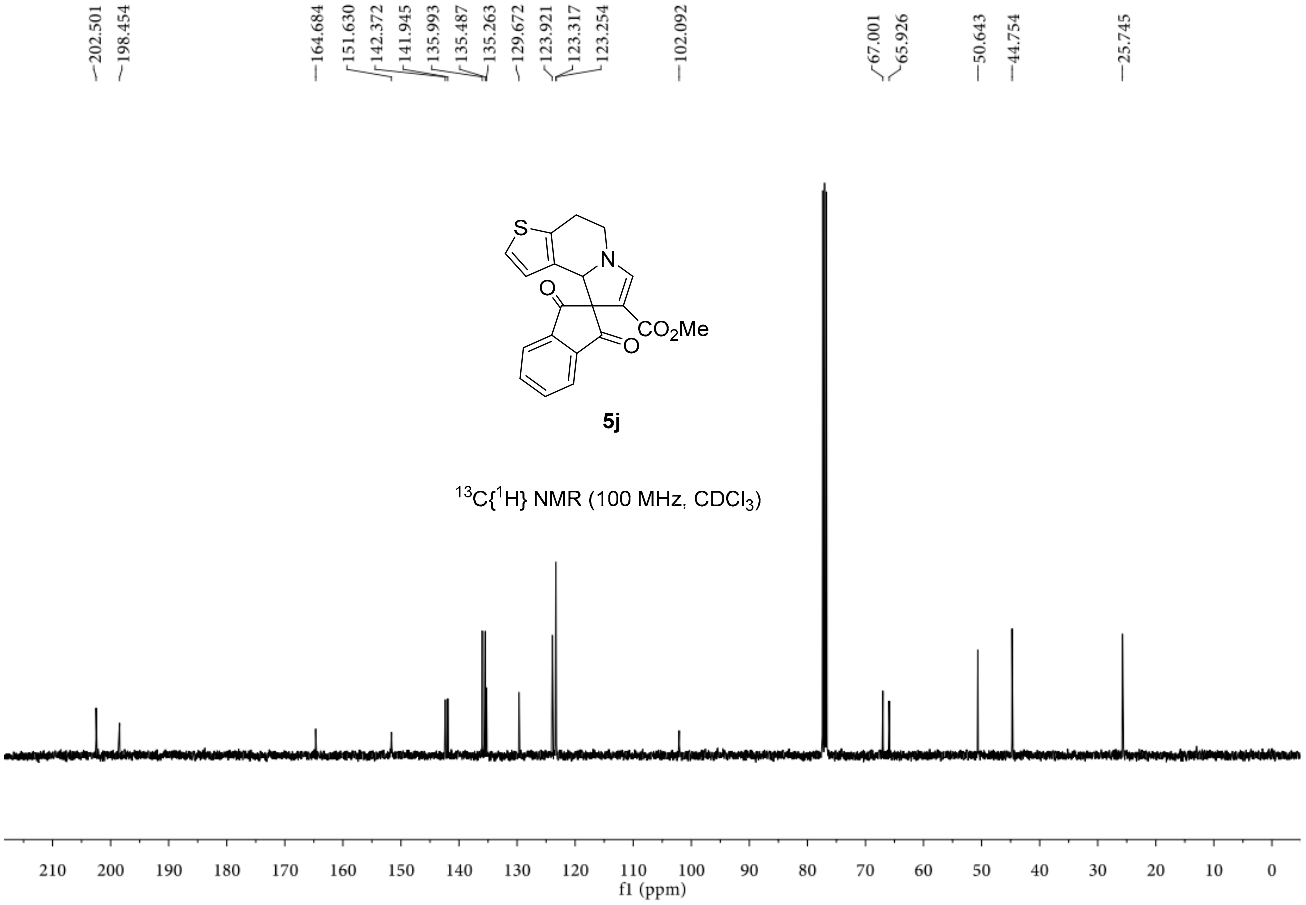

202.501
198.454
164.684
151.630
142.372
141.945
135.993
135.487
135.263
129.672
123.921
123.317
123.254
102.092
67.001
65.926
50.643
44.754
25.745
S
N
O
O
CO2Me
5j
13C{1H} NMR (100 MHz, CDCl3)
f1 (ppm)

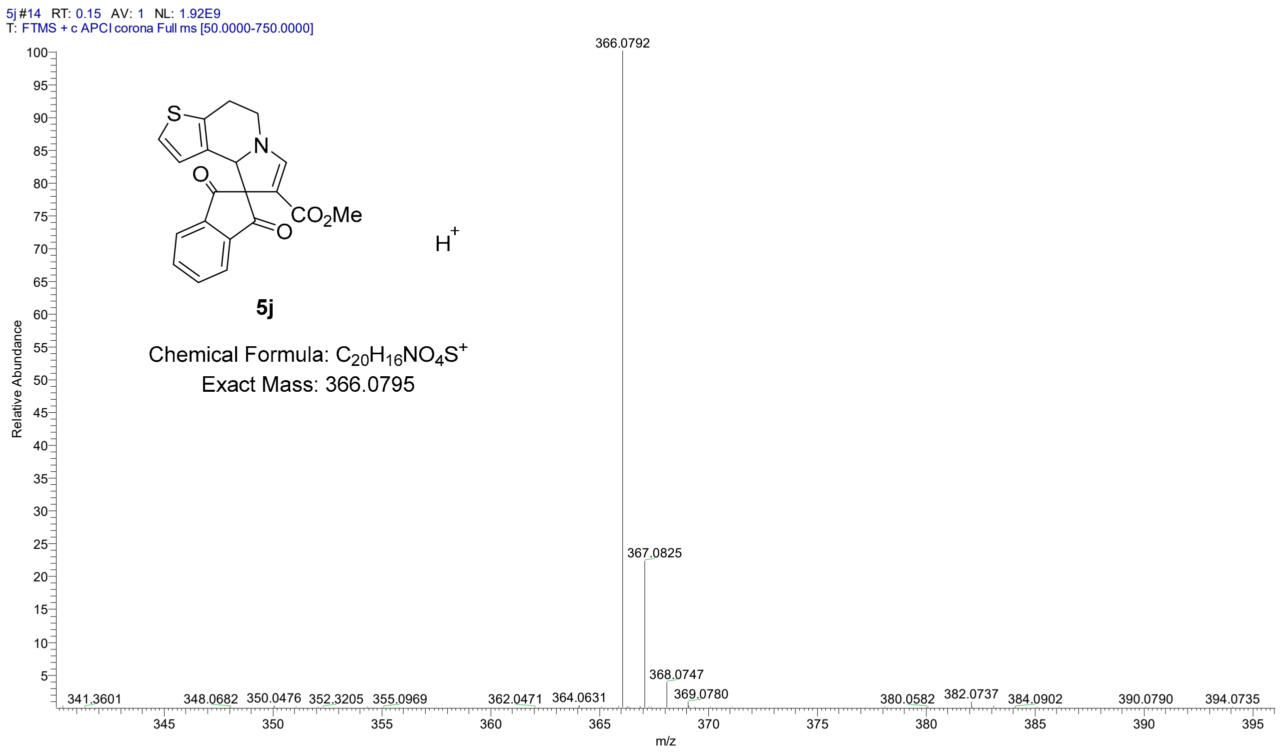

$[M + H]^+$ calcd for $C_{20}H_{16}NO_4S$ 366.0795, found 366.0792.

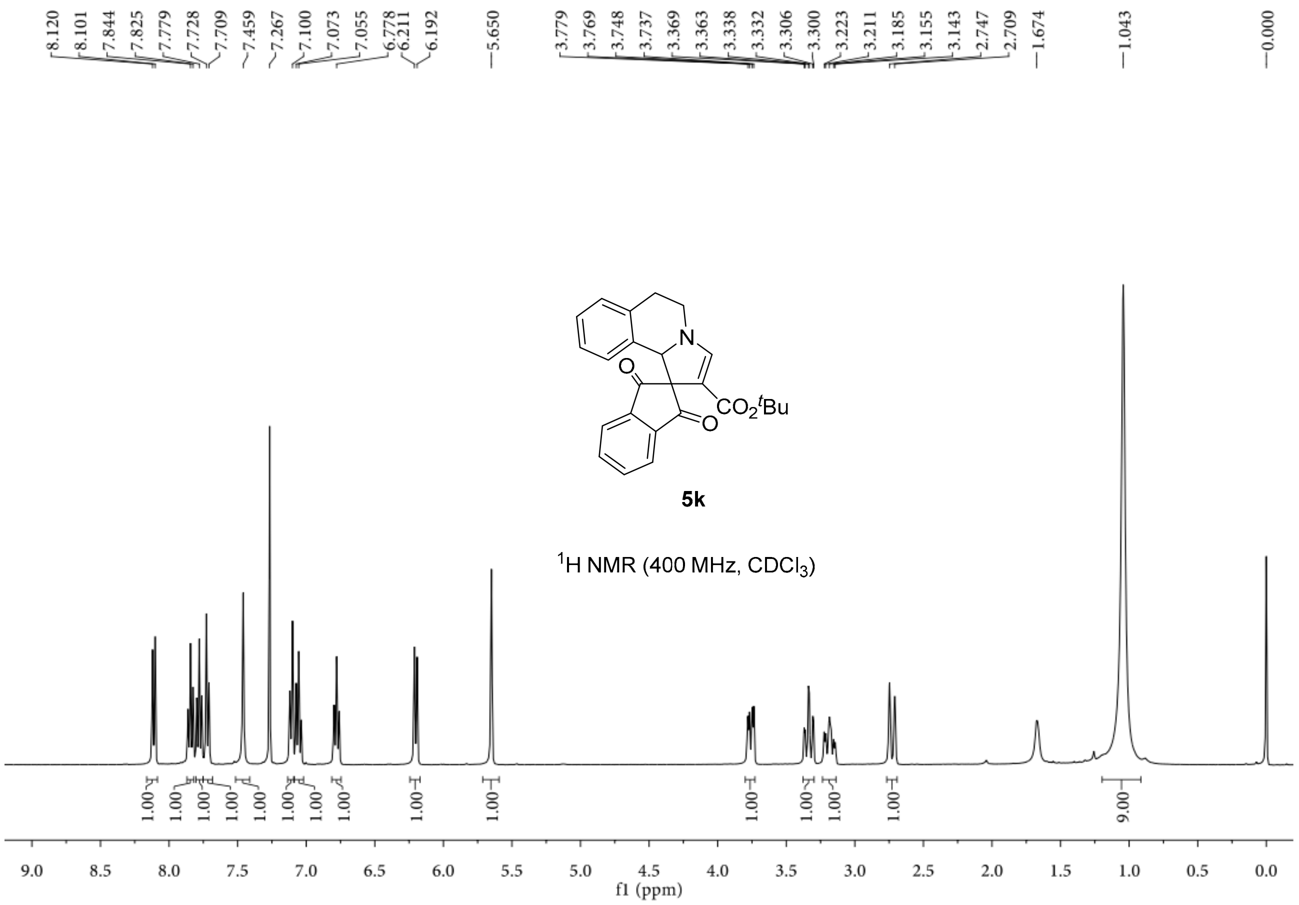
8.120 8.101 7.844 7.825 7.779 7.728 7.709 7.459 7.267 7.100 7.073 7.055 6.778 6.211 6.192 5.650 3.779 3.769 3.748 3.737 3.369 3.363 3.338 3.332 3.306 3.300 3.223 3.211 3.185 3.155 3.143 2.747 2.709 1.674 1.043 0.000
N
O
O
$CO_2{}^tBu$
5k
^1H NMR (400 MHz, $CDCl_3$)
1.00 1.00 1.00 1.00 1.00 1.00 1.00 1.00 1.00 1.00 1.00 1.00 1.00 9.00
9.0 8.5 8.0 7.5 7.0 6.5 6.0 5.5 5.0 4.5 4.0 3.5 3.0 2.5 2.0 1.5 1.0 0.5 0.0
f1 (ppm)

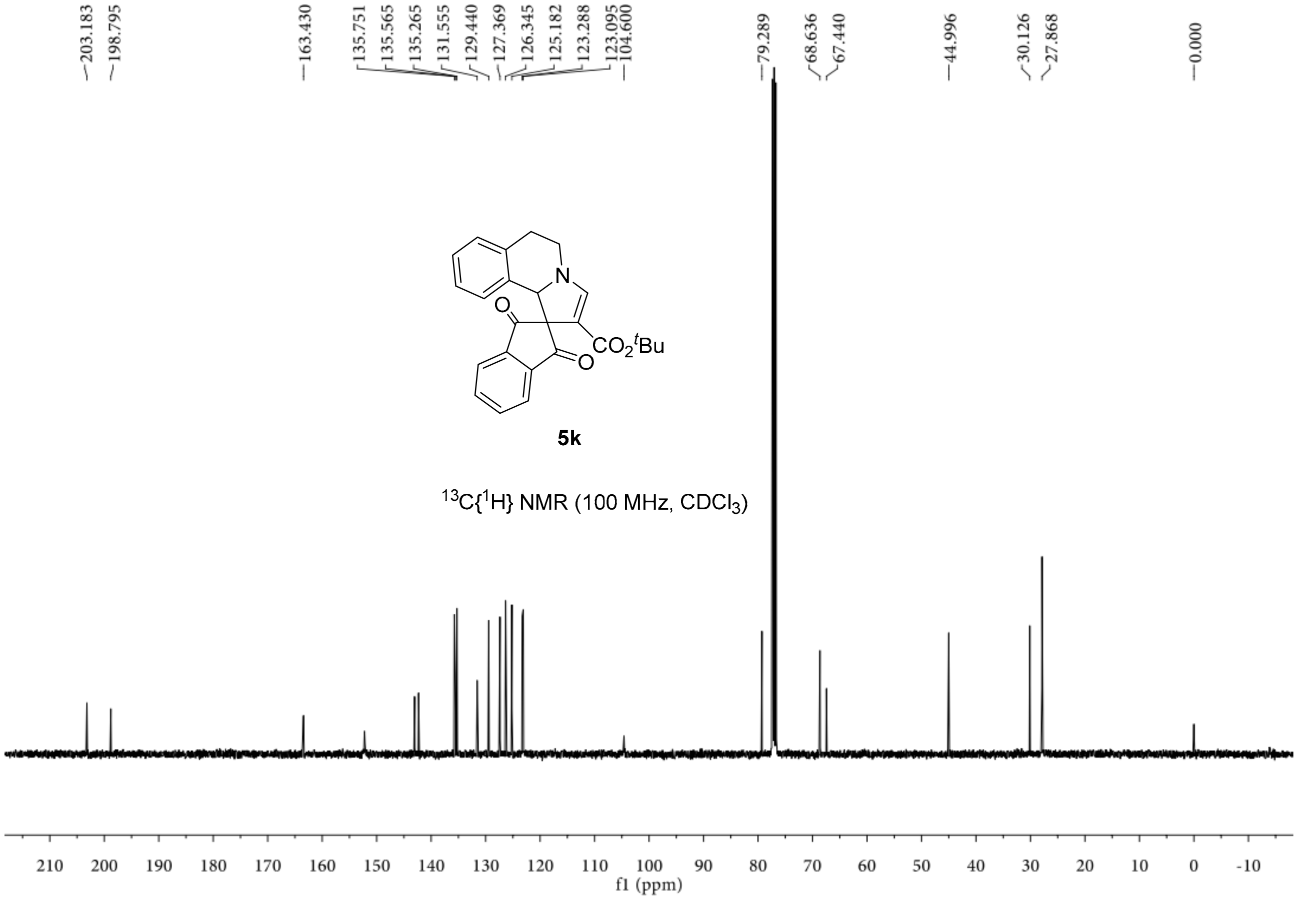
203.183 198.795 163.430 135.751 135.565 135.265 131.555 129.440 127.369 126.345 125.182 123.288 123.095 104.600 79.289 68.636 67.440 44.996 30.126 27.868 0.000
N
O
O
$CO_2{}^tBu$
5k
$^{13}C\{^1H\}$ NMR (100 MHz, $CDCl_3$)
210 200 190 180 170 160 150 140 130 120 110 100 90 80 70 60 50 40 30 20 10 0 -10
f1 (ppm)

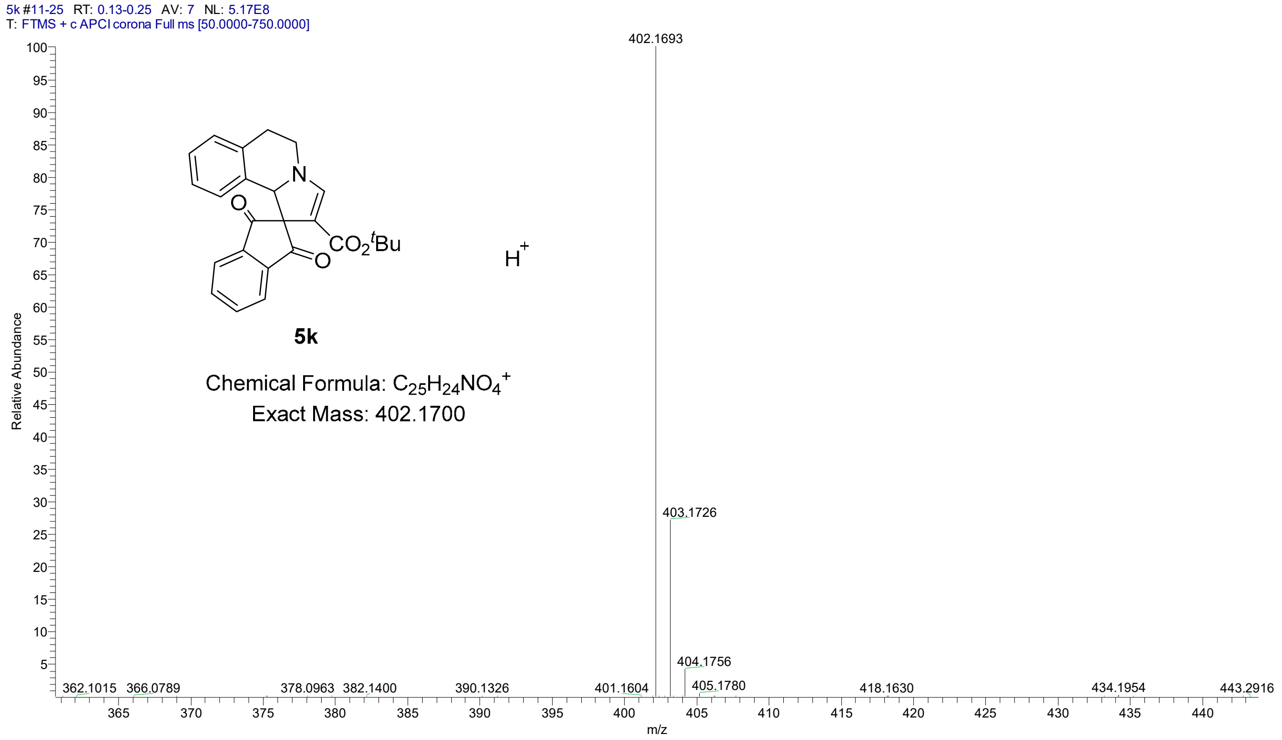

$[M + H]^+$ calcd for $C_{25}H_{24}NO_4$ 402.1700, found 402.1693.

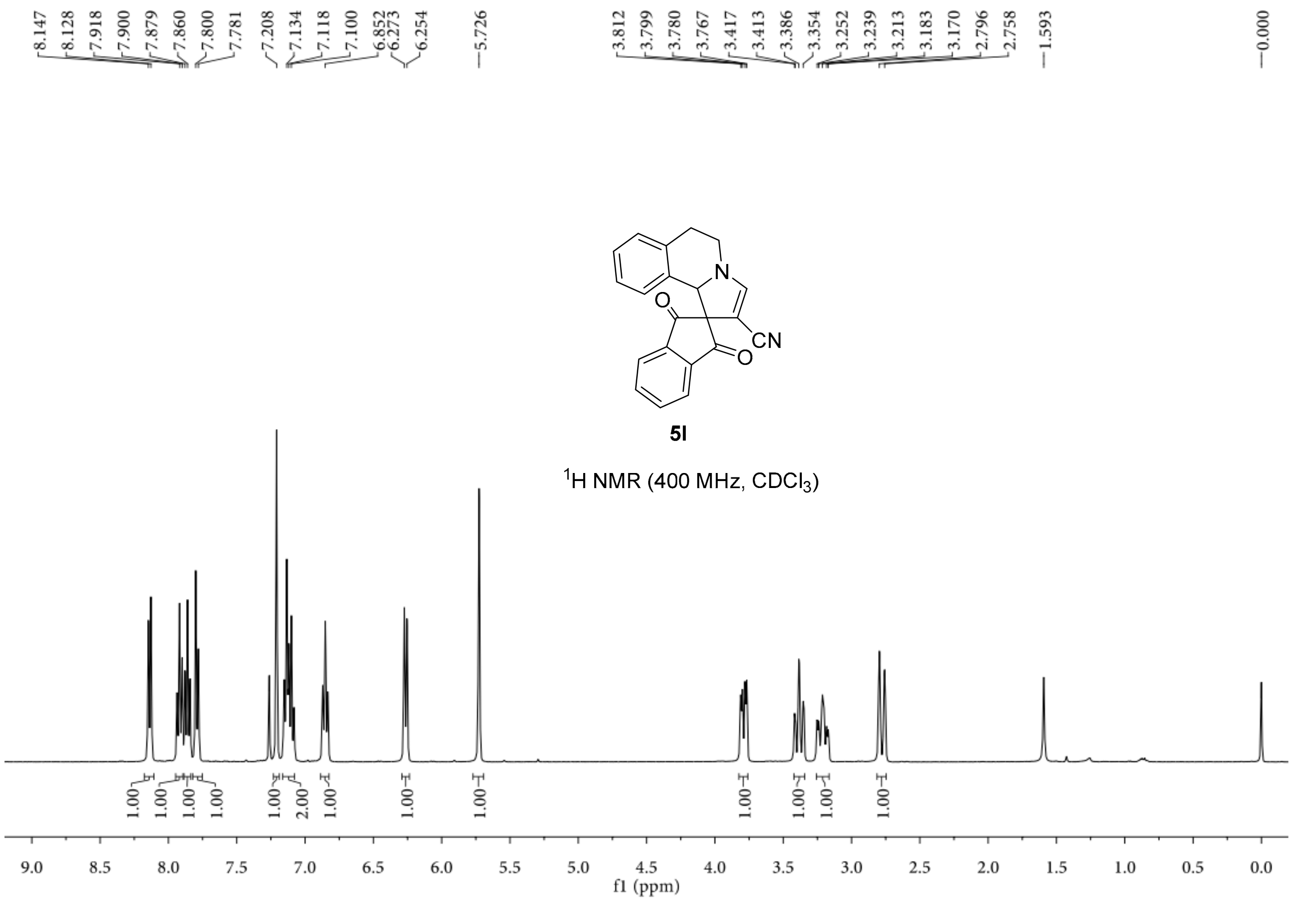

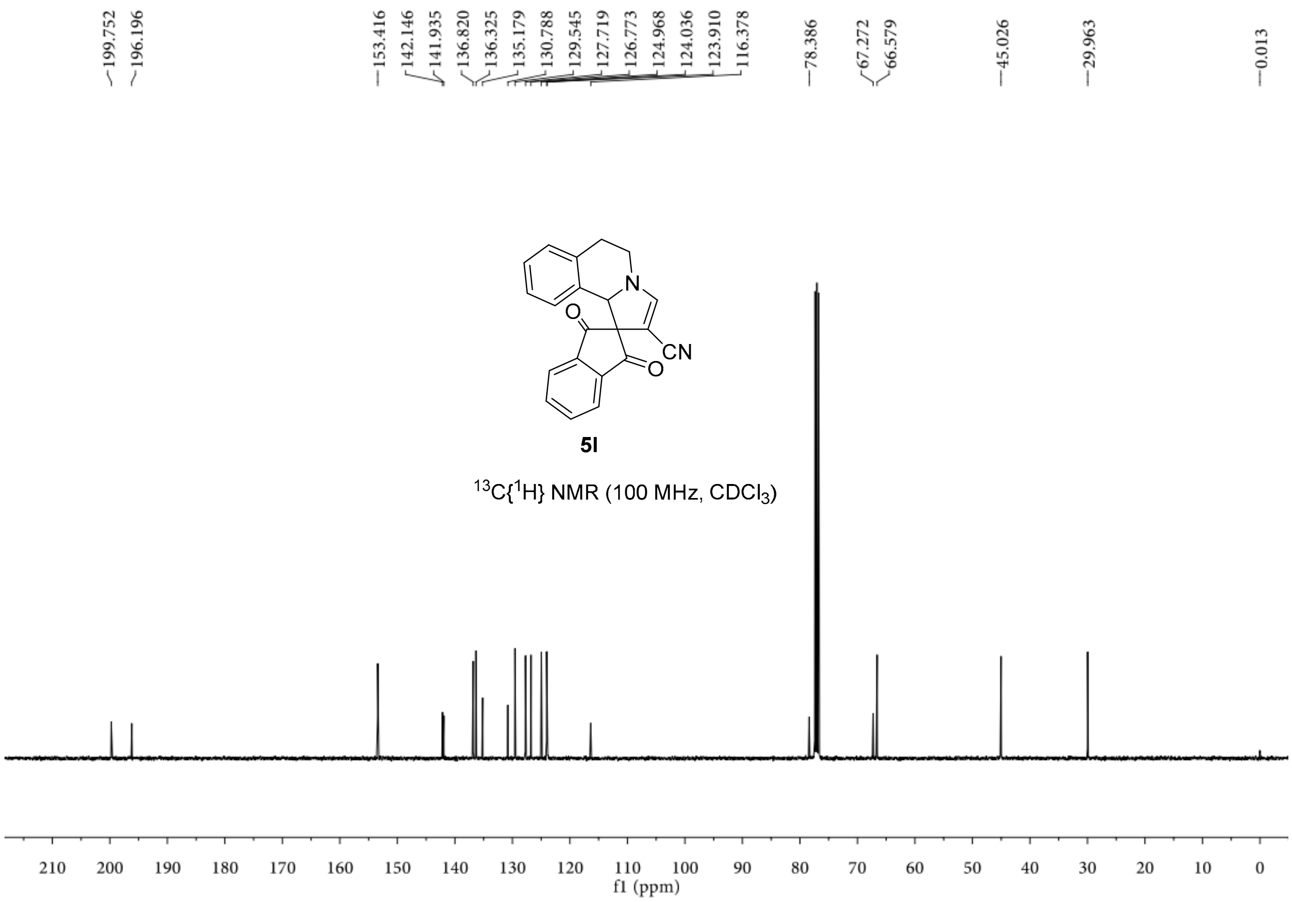

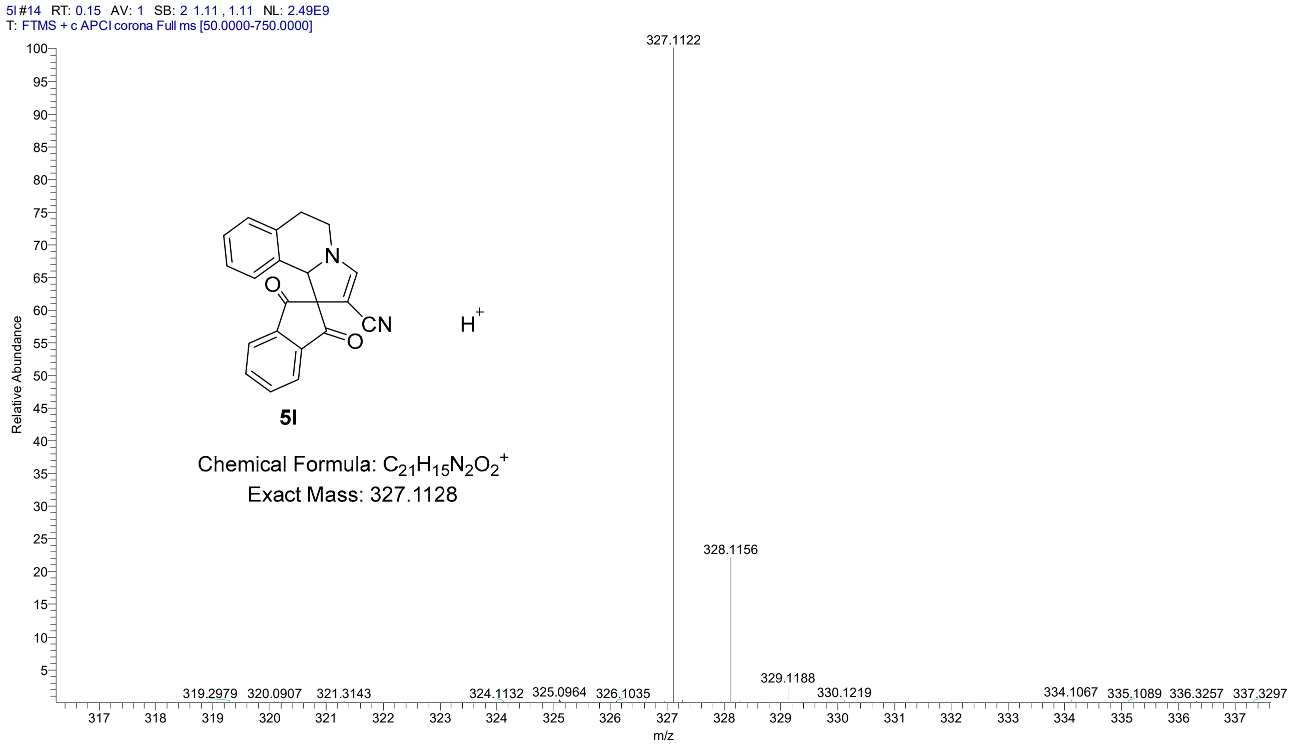

$[M + H]^+$ calcd for $C_{21}H_{15}N_2O_2$ 327.1128, found 327.1122.

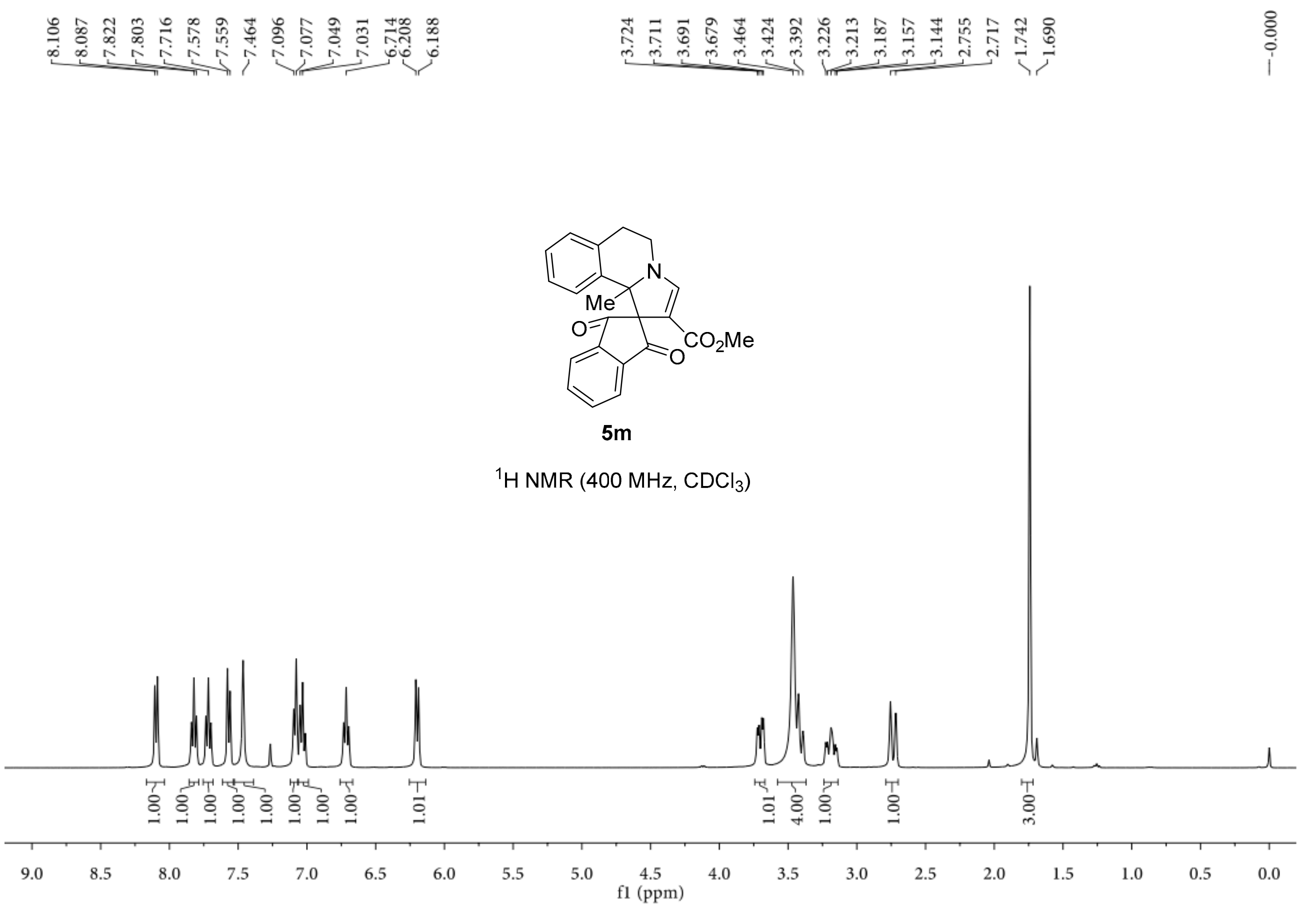
8.106 8.087 7.822 7.803 7.716 7.578 7.559 7.464 7.096 7.077 7.049 7.031 6.714 6.208 6.188
3.724 3.711 3.691 3.679 3.464 3.424 3.392 3.226 3.213 3.187 3.157 3.144 2.755 2.717 1.742 1.690
-0.000
Me
N
O
O
CO2Me
5m
1H NMR (400 MHz, CDCl3)
1.00 1.00 1.00 1.00 1.00 1.00 1.00 1.00 1.01
1.01 4.00 1.00 1.00 3.00
f1 (ppm)

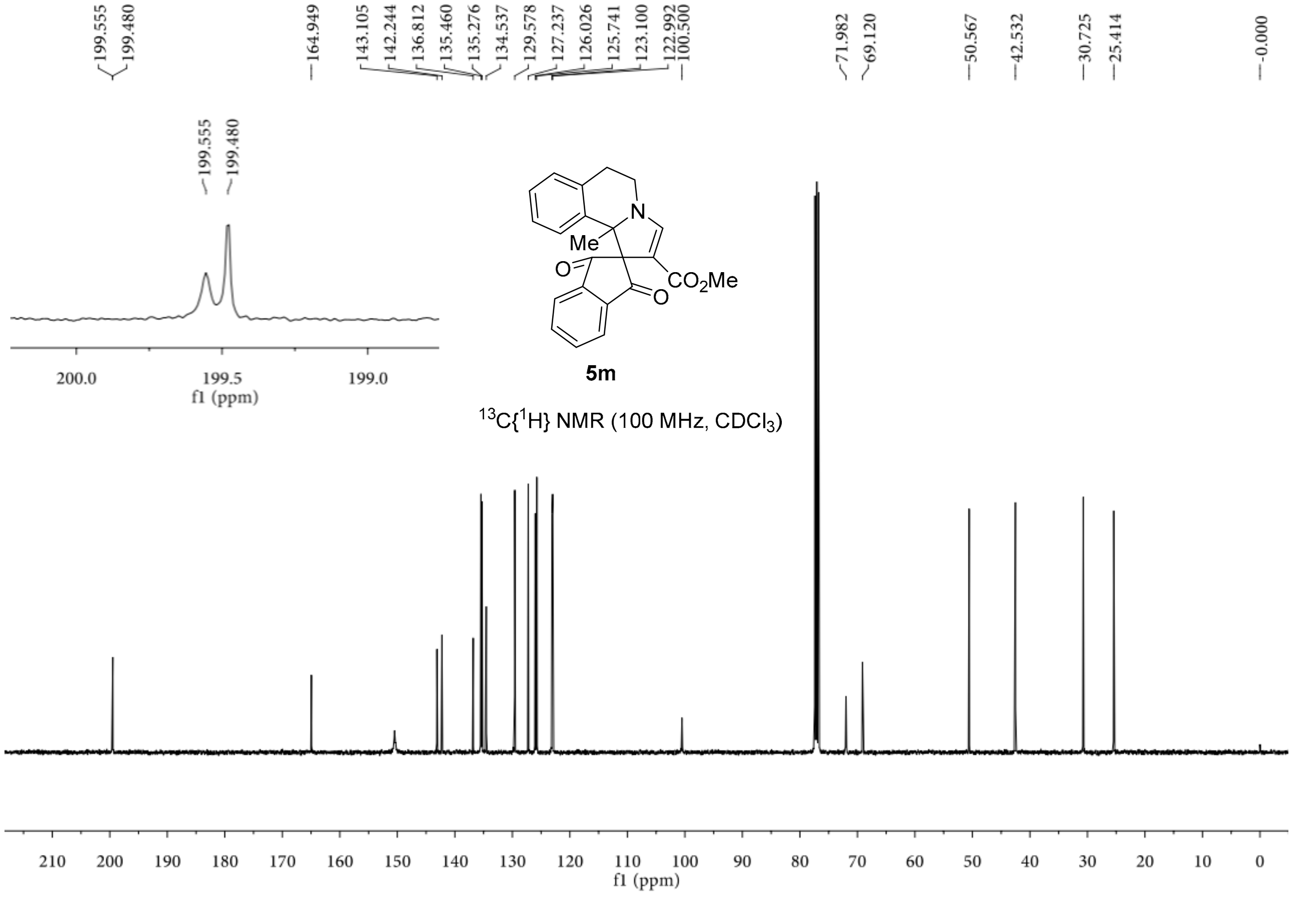
199.555 199.480
164.949
143.105 142.244 136.812 135.460 135.276 134.537 129.578 127.237 126.026 125.741 123.100 122.992 100.500
71.982 69.120
50.567
42.532
30.725 25.414
-0.000
199.555 199.480
f1 (ppm)
Me
N
O
O
CO2Me
5m
13C{1H} NMR (100 MHz, CDCl3)
f1 (ppm)

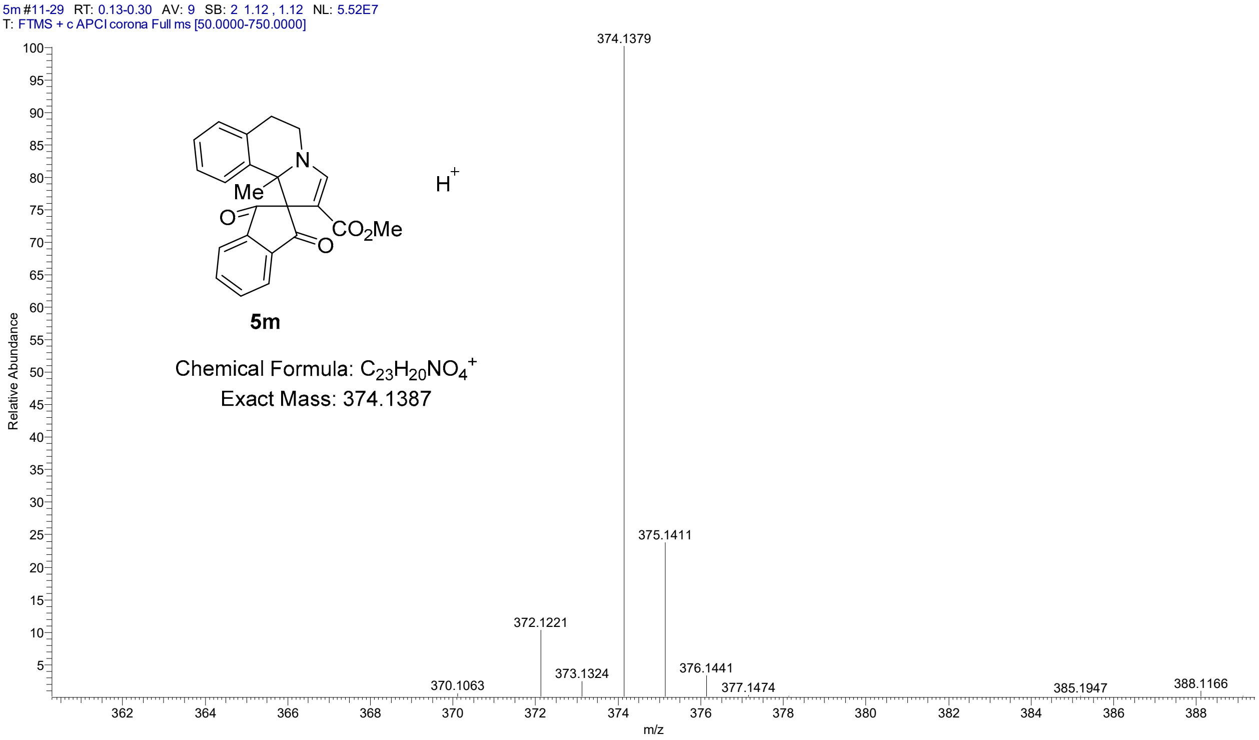

$[M + H]^+$ calcd for $C_{23}H_{20}NO_4$ 374.1387, found 374.1379.
